# A Parameterized Model of Amylopectin Synthesis Provides Key Insights into the Synthesis of Granular Starch

**DOI:** 10.1371/journal.pone.0065768

**Published:** 2013-06-07

**Authors:** Alex Chi Wu, Matthew K. Morell, Robert G. Gilbert

**Affiliations:** 1 Tongji School of Pharmacy, Huazhong University of Science and Technology, Wuhan, Hubei Province, China; 2 Centre for Nutrition and Food Sciences, Queensland Alliance for Agricultural and Food Innovation, The University of Queensland, Brisbane, Queensland, Australia; 3 Food Futures National Research Flagship, CSIRO, Canberra, Australian Capital Territory, Australia; 4 Plant Industry, CSIRO, Canberra, Australian Capital Territory, Australia; Tata Institute of Fundamental Research, India

## Abstract

A core set of genes involved in starch synthesis has been defined by genetic studies, but the complexity of starch biosynthesis has frustrated attempts to elucidate the precise functional roles of the enzymes encoded. The chain-length distribution (CLD) of amylopectin in cereal endosperm is modeled here on the basis that the CLD is produced by concerted actions of three enzyme types: starch synthases, branching and debranching enzymes, including their respective isoforms. The model, together with fitting to experiment, provides four key insights. (1) To generate crystalline starch, defined restrictions on particular ratios of enzymatic activities apply. (2) An independent confirmation of the conclusion, previously reached solely from genetic studies, of the absolute requirement for debranching enzyme in crystalline amylopectin synthesis. (3) The model provides a mechanistic basis for understanding how successive arrays of crystalline lamellae are formed, based on the identification of two independent types of long amylopectin chains, one type remaining in the amorphous lamella, while the other propagates into, and is integral to the formation of, an adjacent crystalline lamella. (4) The model provides a means by which a small number of key parameters defining the core enzymatic activities can be derived from the amylopectin CLD, providing the basis for focusing studies on the enzymatic requirements for generating starches of a particular structure. The modeling approach provides both a new tool to accelerate efforts to understand granular starch biosynthesis and a basis for focusing efforts to manipulate starch structure and functionality using a series of testable predictions based on a robust mechanistic framework.

## Introduction

Starch, a branched glucose homopolymer, contains two types of glucans: amylose (degree of polymerization (DP) of 100–10,000, largely unbranched) and amylopectin (larger, highly branched, and typically constituting ∼75 wt% of the total starch). Short chains with DP 30 on amylopectin molecules form double-stranded *α*-helices in the native starch, which align to facilitate crystallization to give crystalline lamella. Some amylopectin chains grow longer (up to DP ∼ 100) and serve as the crystalline-lamella connecting chains. Crystalline lamellae alternate with amorphous lamellae, with a periodicity of ∼9 nm [1], giving the semi-crystalline structure of amylopectin within granular starch. Growth of starch granules generally proceeds radially outwards from the core, giving concentric layers of semi-crystalline growth rings, which alternate with amorphous growth rings. The semi-crystalline structure formed by amylopectin branches is of biological and economic importance, as this structure allows plants to store carbon at high density (∼1.6 g cm^–3^), in an osmotically inert form. Several models have been proposed to describe the formation of amylopectin clusters. Ball et al. described a “discontinuous synthesis model” [2]. When chains reach minimum size to allow branching, intensive branching follows. Trimming by debranching enzymes proceeds, leaving only tightly spaced branches. The next amorphous lamella is generated and then allows for another turn of discontinuous synthesis. A “two-step branching and improper branch clearing model” proposed by Nakamura [3] gives the following explanation for the formation of amylopectin clusters. Glucan chains are suggested to transfer from clusters of branches in a nascent crystalline lamella and cause branching in the adjacent amorphous lamella; further elongation of these chains are suggested to enable SBEs to act on them to produce branches in the new cluster so as to form the next crystalline layer. However, because of the complexity of the starch synthesis mechanism, some fundamental aspects of the synthesis of the amylopectin crystalline array remain to be elucidated.

Starch biosynthesis in storage tissues, in particular in cereal endosperms, involves the concerted actions of various types of biosynthetic enzymes, which include multiple isoforms [4], [5], [6]. However, the modes of enzymatic actions, which affect the rate at which the enzymes operate, are yet to be rigorously defined. For example: (1) the phosphorylation status of the enzymes is not yet known or described; (2) no account has been taken of possible conformation changes that may occur as part of enzyme complexation [5].

A general consensus as to the core enzymatic machinery involved is that glucan chains formed by the transfer of the glucosyl moiety of ADP-glucose to the non-reducing end of a pre-existing glucans via *α*-(1→4) glycosidic linkages by four types of soluble starch synthases (SSI–SSIV), and granule-bound starch synthase (GBSSI in endosperm, GBSSII in non-endosperm tissues). Transfer from UDP-glucose is also possible, albeit at low rates [5]. Soluble starch synthases are considered to be primarily involved in amylopectin synthesis and granule bound starch synthases in amylose synthesis. Mutants lacking SSI show deficiencies in short chains with *X* (the mathematical symbol for DP) between 6–12 (e.g. [7]). Lack of SSII is associated with a deficiency of chains with 12≤ *X* <30 [8] while SSIII may play a role in elongating longer chains (*X* ≥30) [9]. In *Arabidopsis,* SSIV has been shown to be associated with starch granule initiation [10], [11]; however, its function has not been determined in cereal endosperms. Biochemical studies show the biosynthetic enzymes have substrate specificity: for example, SSI is said to elongate very short glucan branches (4≤ *X* ≤7) and the products are subsequently elongated by SSII [5].

**Figure 1:**
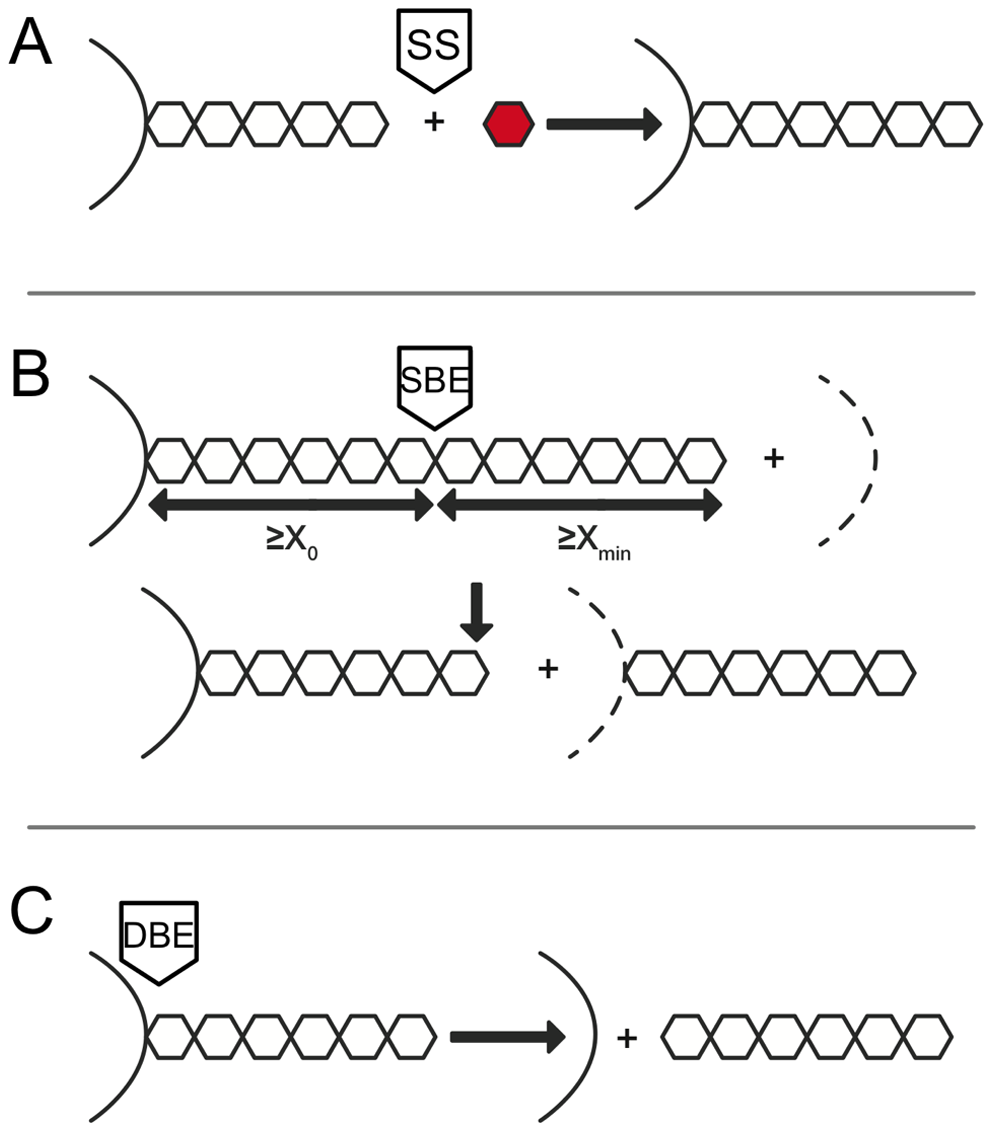
The mechanism of actions of three core enzymes in starch biosynthetic considered in the model. Starch synthases (SSs) transfer ADP-glucose (red hexagon) to the non-reducing end of a pre-existing *α*-(1⟶4)-linked glucan. Starch branching enzymes (SBEs) transfer the cleaved chain onto a random position–which may include the original branch–via an *α*-(1⟶6) link. The length of the transferred portion by SBEs has to be ≥ *X*_min_ and that of the remaining stub must be ≥ *X*_0_. Debranching enzymes (DBEs) hydrolyze *α*-(1⟶6) linkages, thereby removing the whole chain. These enzymatic schemes are focused on the form of glucans in terms of the CLD rather than the actual products. Together, one of each of SS, SBE and DBE, regardless of the actual isoforms, make up an enzyme set.

Starch branching enzymes (SBEs) create new glucan branches, involving cleavage of an *α*-(1→4) linkage and transfer of the released reducing end to a glucose residue on either the original or another glucan chain via an *α*-(1→6) linkage. There are two types of SBEs: SBEI and SBEII. In monocots, two classes of SBEII are present: SBEIIa and SBEIIb (e.g. [12], [13]). Biochemical studies show SBEI preferentially transfers longer chains from long chains while SBEII tends to transfer shorter chains from more highly branched substrates [14]. There are two restrictions in SBEs (Figure 1): the moiety that is transferred must be equal or longer than a minimum *X*, *X*_min_, which is ∼6–7 glucose residues for the SBEII type (e.g. [15]), while the remaining moiety must be of a *X* not less than a minimum value *X*_0_, which is ∼6 [16]. Together, the actions of SSs and SBEs give rise to branched glucans.

The Hizukuri model [17] of amylopectin is based on the non-random clustering of *α*-(1→6) branch points in the amylopectin molecule; a significant body of evidence suggests that this clustering is essential to allow the alignment of external chains of amylopectin in order to form double helices and then crystalline lamellae. The roles of debranching enzymes (DBEs, specifically the isoamylases) have been suggested to include removal of the “improperly positioned” branches: branches that because of their position prevent local crystallization [2], [3], [18], [19], [20]. It is assumed that once a branch is removed by DBEs, it is no longer part of the growing glucan molecule, and instead is degraded and the glucan reutilized. In vitro, the unrestricted action of isoamylases generates linear glucans, which together form the chain-length distribution (CLD). Hence the CLD does not contain direct information about the non-random clustering of branches on amylopectin molecules.

Other enzymes have also been proposed to play roles in the biosynthesis of starch; however, these roles are not consistent across tissues and species and so are not considered part of the core enzymatic machinery. For example, it is proposed that disproportionation enzyme (D-enzyme) is directly involved in amylopectin synthesis in *Chlamydomonas reinhardtii* [21]. However, inactivation of D-enzyme in *Arabidopsis thaliana* leaves does not affect starch synthesis or amylopectin CLD significantly [22]. It has been shown that a D-enzyme, with similar enzymatic activity to that in *Chlamydomonas*, is also present in wheat endosperms; however, no information on the role of D-enzyme in cereal starches is available [23].

SS, SBE and DBE are the three core enzyme classes that are important in utilizing ADP-glucose to synthesize starch in cereal endosperms. Here, adopting our earlier model [24], a theoretical “enzyme set” is defined as a group of three enzymes, which includes one of each of SS, SBE and DBE, regardless of the actual isoforms. For example, the SS in different enzyme sets could be forms of a particular isoforms of SSI, SSII, SSIII and SSIV that have different activity depending on their complexation state, post-translational modification, or other temporally or physically distinguished regulatory effects. The terminology used for the model is that the SS belonging to *enzyme set (i)* is denoted SS(i). In turn, SS(ii) is used to indicate the SS in enzyme set (ii) and so on. This also applies to *X*_0_ and *X*_min_ (e.g. *X*_0(i)_ is a minimal constraint on SBE(i)). No direct relationship is assumed between the enzyme sets defined for the purposes of this model, and members of gene families defined by genetic studies. For example, SSI, SSIIa, SSIIIa and SSIV in the cereal endosperm do not equate to SS(i), SS(ii), SS(iii) and SS(iv). Such associations will be the focus of future research.

The CLD of granular starch is referred to as the lamellar CLD. It is denoted here *N*_de_(*X*): the relative number distribution of glucan chains with DP of *X* (the subscript “de” denoting the fact that these glucan chains are obtained from unrestricted debranching of starch). The lamellar CLD in the order of up to *X* ∼ 100 is predominantly amylopectin CLD; amylose chains are significantly longer. The lamellar CLD is currently best obtained using fluorophore-assisted carbohydrate electrophoresis (FACE) [25], [26]; less precise data can also be obtained using high-performance anion exchange chromatography (HPAEC) or by size-exclusion chromatography (SEC) [27], [28].

**Figure 2:**
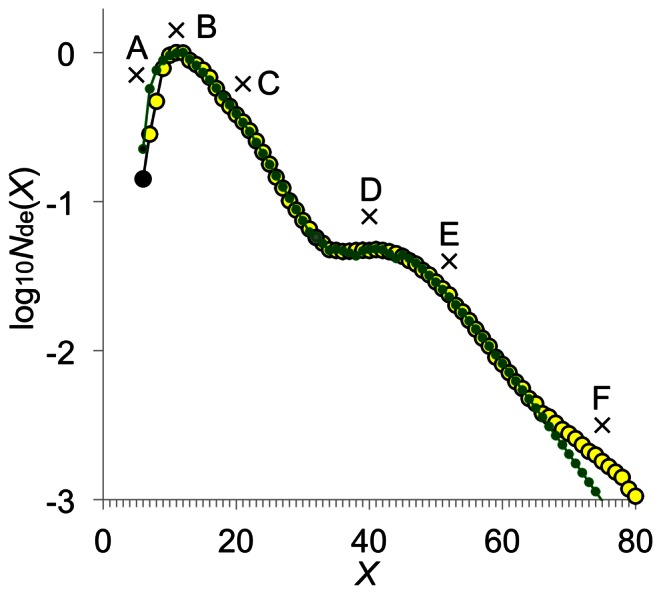
Rice (cv Nipponbare) amylopectin CLD fitted with the substrate-competing model. The amylopectin CLD was obtained by FACE [32]. *X* is the number of glucose residues on glucans released by unrestricted debranching of the starch. The black- and gray-filled circles are *X* = 6 and 32, respectively. The fitting to the overall experimental CLD (yellow filled circles) is given as green filled circles. The range of fitting *X* up to ≈ 60–70 is considered. Fitting procedures are given the Model section. Crosses mark the features of the CLD.

The amylopectin CLD in cereal endosperms generally shows a number of distinct features (Figure 2). These features come from particular enzyme sets and restrictions on the biosynthetic enzymes (e.g. the two minimum chain length requirements for SBEs, *X*_min_ and *X*_0_), which are summarized as follows. Feature A is due to one of the SBE minimum chain length requirements, *X*_0(i)_ for SBE(i). Depending on the values of *X*_0(i)_ and *X*_min(ii)_, Feature A can appear as a maximum, shoulder or as part of the global maximum (Feature B). This is explored in a later section. Feature B is the global maximum which appears between *X*_min(i)_ and *X*_0(i)_+*X*_min(i)_. Feature C is a small bump arising from the SBE(ii) restrictions of *X*_0(ii)_ and *X*_min(ii)_ in the same way as *X*_0(i)_ and *X*_min(i)_. Features A, B, and C are ascribed to enzyme sets (i) and (ii). These enzyme sets synthesize chains confined to single lamellae (SL), wherein these chains are arranged in crystalline lamellae. The SL chains dominate the range 6≤ *X* ≲ 30. Long SL chains protruding the SL range enter the contiguous amorphous lamella, here named the trans-lamella (TL) range. These long chains are crystalline-lamella connecting chains [17] and span to an adjacent SL range which is subsequent to the TL range. Features D and E are analogous to Features B and C, except that they are in the TL range. Analogous to Features A, B and C, Features D and E are ascribed to enzyme sets (iii) and (iv). The TL equivalent of Feature A is not apparent. Feature F indicates chains that span beyond two adjacent SL ranges which are separated by a TL range in between. Examination of whether the equivalents of Features A, B, and C are distinguishable in the Feature F range requires a larger CLD range and even more accurate data than currently available. The higher *X* range measured by FACE is prone to error because of current technical limitations; neither HPAEC nor SEC can resolve fine features in this range.

The amylopectin CLDs in the endosperms of maize [29], rice [30] and barley [31] do not change significantly throughout grain development. The same phenomenon is also observed in *Arabidopsis* leaves, regardless of the diurnal nature of leaf physiology [19]. These show that amylopectin CLDs can be approximated as being in a steady state. A steady-state CLD should not be confused with an unchanged total amount of starch per unit volume. It is the *relative* abundance of chains between different *X* (i.e., the CLD) that does not change in time, while the *total* concentration of chains can increase during grain development, or in leaves in the photoperiod.

It will be shown, by modeling the CLD of amylopectin in cereal endosperms, that in order for crystalline starch to form in cereal endosperms such as rice, restrictions apply on the ratios of certain enzymatic activities of SSs, SBEs and DBEs. DBE will be seen to be essential for crystalline amylopectin synthesis. The model also provides a mechanistic basis for understanding how successive arrays of crystalline lamellae are formed. Further, the model allows the amylopectin CLD in cereal endosperms to be quantitatively represented by a small number of parameters: ratios of enzymatic activities and the two minimum SBE constraints (i.e. *X*_0_ and *X*_min_). This parameterization of CLDs provides both a useful tool to represent CLDs, for example to explore statistically meaningful structure-property relations, and also a new tool for understanding the biosynthesis of granular starches.

## Results

### Single-lamella (SL) Chains

The SL CLD is governed by the actions of enzyme sets (i) and (ii). Two alternative models for this are presented here, with small but significant differences between them. One, termed the “substrate-competing model”, is consistent with the assumption that enzyme sets (i) and (ii) act on the same pool of substrate(s), perhaps as a result of enzyme complexes (see Model section for the evolution equation which describes the SL chains, Eqn 1). The alternative is termed the “independent substrates model”, when enzyme sets (i) and (ii) predominantly act on distinct populations of substrate(s), perhaps as a result of substrate specificities. The substrate-competing model is new; the independent substrate model is an extension of our earlier treatment [24]. Evidence will be provided to demonstrate that which of the two alternative models is applicable in a given case depends on the particular plant species; it is also possible that differing models may apply in different organs, under different environmental conditions, or during different stages of organ development.

The SL CLD can only reach a steady state with restricted values of certain SL kinetic parameters, as detailed in the Model section. These SL kinetic parameters for the SL enzyme sets (i) and (ii) in the substrate-competing model are denoted *β*_(i)_, *X*_0(i)_, *X*_min(i)_, *β*_(ii)_, *X*_0(ii)_ and *X*_min(ii)_. The quantities with the symbol *β* are ratios of enzyme activities, defined in Eqns 2–3. The actual values of the fitted parameters for the amylopectin CLD (Figure 2) of a rice variety (cv Nipponbare) are discussed in a later section. For the independent-substrate model, there is one additional parameter, which is the relative contributions of sets (i) and (ii), denoted *h*_(ii/i)_.

**Figure 3:**
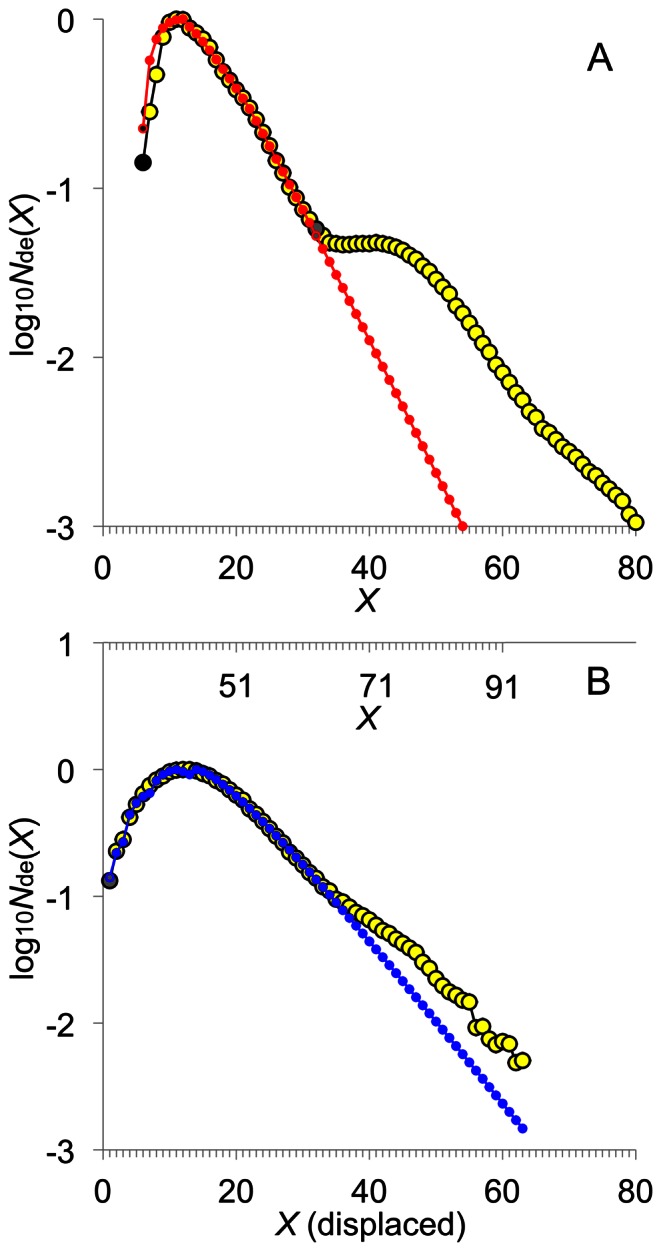
Components of the CLD, given in Figure 2, fitted with the model. (A) Fitting (red filled circles) to single-lamella (SL) chains with the substrate-competing model. *X* = 32 marks the apparent length of chain needed for the chains to enter the contiguous amorphous lamella (Figure 5). Where the slope changes significantly in Feature F gives the range of presumed three-lamellae-spanning chains. (B) Fitting (blue filled circles) to the type-2 TL chains with the substarte-competing model, which is the difference between the experimental and the calculated SL CLD in (A) and displaced (details in the Model section). The actual *X* is shown on the upper abscissa of (B).

The substrate-competing model quantitatively reproduces Features A, B and C in the SL range of the rice amylopectin CLD (Figure 3A): (1) the shape and location of Features A and B. Feature A appears as part of the curve for the global maximum (Feature B). Feature B, the global maximum occurs between *X*_0(i)_ and *X*_0(i)_+*X*_min(i)_ in the present case; (2) the shape and location of Feature C, which corresponds to *X*_0(ii)_ and *X*_min(ii)_; and (3) the steepness of the nearly linear slope of log *N*_de_(*X*). Although enzymes sets (i) and (ii) both contribute significantly over the whole SL range, set (i) is largely responsible for the shape for the global maximum and the nearly linear slope, and enzyme set (ii) is largely responsible for the bump (Feature C) on the nearly linear slope.

As stated, the substrate-competing model provides an excellent fit to the rice SL CLD. The independent substrate model can also fit the SL range of the rice amylopectin CLD (Figure S1A), although the fit is not as good (see Discussion).

The substrate-competing model cannot quantitatively reproduce the prominent Feature C in the CLD of the *Triticeae* tribe, such as wheat (Figure S5A). A good fit is obtained by treating enzyme sets (i) and (ii) as not competing for the same substrate, which means obeying a separate evolution equation in the form of Eqn 1 for the CLD for the two additional enzyme sets. Having a given range (i.e. SL or TL ranges) of the CLD with contributions from enzymes sets which do not compete for the same substrates is the “independent substrate model”. This model fits the wheat data (Figure S2A). This difference in rice and wheat may lie within the mode of enzymatic actions in different species, as hypothesized earlier.

### Chains Located in more than a Single Lamella

The calculated SL CLD for rice (Figure 3A) also indicates that there are long SL chains (*X* ≥32 for the present results). These long SL chains, which would protrude out of single lamellae, are denoted *Type–1 trans-lamella (TL) chains*. They contribute to a portion of the chains in the high *X* range (i.e. 32≤ *X* <60–70). While the fit for shorter SL chains is excellent, the calculated SL CLD at high *X* does not resemble the experimental one. The *X* at which the calculated CLD significantly deviates from the experimental CLD is taken to define the start of the trans-lamella (TL) range (Figure 3A; 32≤ *X* <60–70) where the chains are no longer quantitatively described by the SL enzyme sets alone. The CLD in the high *X* range must come from contributions from additional enzyme sets. It could be supposed that the additional enzyme sets also act in a substrate-competing manner with the SL enzyme sets. However, this is not the case: incorporating the contributions from additional enzyme sets in Eqn 1 is unable to reproduce the experimental CLD for *X* ≥32 (Text S2 and Figure S3).

The only way that the CLD can be quantitatively modeled for both rice and wheat for *X* ≥32 is if there are additional enzyme sets operating, not competing for the same substrate(s), but independently from the SL enzyme sets: the independent substrate model. The additional enzyme sets are termed the TL enzyme sets: these enzyme sets (iii) and (iv) for rice are again treated with the substrate-competing model as for the SL enzyme sets. The calculated CLD from the TL enzyme sets are denoted *Type–2 TL chains.*

The TL enzyme sets give the TL kinetic parameters denoted *β*_(iii)_, *X*_0(iii)_, *X*_min(iii)_, *β*_(iv)_, *X*_0(iv)_ and *X*_min(iv)_. The ratios of enzymatic activities for enzyme set (iii) and (iv) are defined analogously to Eqns 2–3. These parameters have the same steady-state restrictions to those for the SL kinetics parameters (see Model section). The calculated type-2 TL CLD is fitted to the corresponding experimental CLD (Figure 3B), obtained as the difference between the experimental CLD and the calculated SL CLD (detailed in the Model section). The fitted type-2 TL CLD quantitatively reproduces the observed features: Features D and E. The Feature A-equivalent feature, not apparent in the overall experimental CLD (Figure 2), is revealed in the type-2 TL CLD obtained by this subtraction. A significant change in the near-linear slope is seen at *X* = 60–70. Chains longer than this are presumed to span three lamellae and to be governed by additional independent enzyme sets. Accurate data on these chains cannot be obtained with current techniques, and thus are not treated here.

In the substrate-competing model, the SL and TL enzyme sets are independent, and thus a parameter *h*_(iii/i)_, defined in the Model section, is required to specify the relative abundance of the CLDs generated from both kinetics; this parameter is obtained by fitting.

In the independent substrate model, the kinetics of each enzyme set is independent of the others. Therefore, the relative abundance of the CLDs from enzyme sets (ii), (iii) and (iv) are ratios in relation to that of enzyme set (i) and are specified by the parameters *h*_(ii/i)_, *h*_(iii/i)_ and *h*_(iv/i)_, respectively. The quantity *h*_(ii/i)_ was denoted *a*_2_/*a*_1_ in our previous paper [24]; the current terminology is more appropriate.

The overall calculated CLD in the substrate-competing model is obtained by summing the calculated CLDs from both the SL and the TL enzyme sets. Note that it is the *N*_de_(*X*) (not log *N*_de_(*X*)) that are summed, although the data are best presented as log *N*_de_(*X*). This overall calculated CLD accurately fits rice amylopectin CLDs (Figure 2).

### Fitting Model Parameters

**Figure 4:**
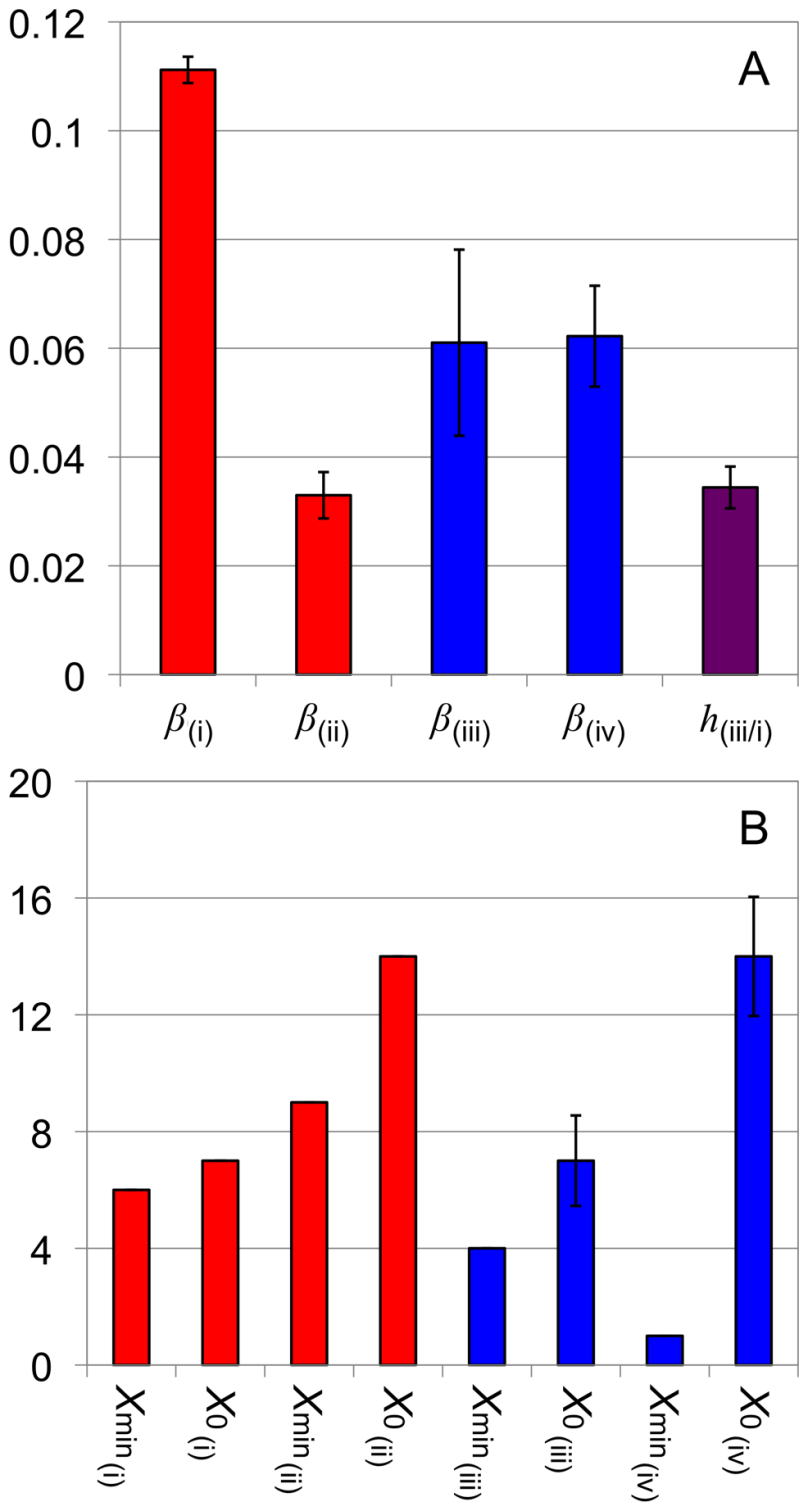
Fitted parameters for six replicates of the CLD described in Figure 2. (A) Averages and ± standard deviations (thin bars) of *β* (the relative branching activity from an enzyme set to that of the total propagation from set (i) and (ii) in the SL range, or (iii) and (iv) in the TL range) from different enzyme sets; *h*_(iii/i)_ is the ratio of the maximum of type-2 TL CLD to that of the SL CLD. (B) The modes (highest repeating values) of *X*_0_ and *X*_min_ (which are discrete variables) from different enzyme sets. Where the thin bars are not seen means the standard deviation is small. The red and blue colors correspond to the calculated SL and type-2 TL CLD in Figure 2.

Non-linear least-squares fitting with the substrate-competing model was performed on the CLD of amylopectin from six replicates of a rice variety, Nipponbare [32]; results are in Figure 4. Despite the number of parameters involved in these fittings, the standard deviation in the fitted values of these parameters from the replicates is quite small, provided that the CLD data are sufficiently precise, as is the SL range in the present case. Replicate data beyond the SL range (*X* ≥32) show more scatter, as reflected in the range of fitted values (e.g. Figure 4, *β*_(iii)_ and *β*_(iv)_). (It is noted that the fitted parameters from the substrate-competing model have no simple relationship to those from the independent-substrate model; Figure S4).

## Discussion

### Amylopectin CLD Synthesis is in a Steady State which Requires the Involvement of Debranching Enzymes

Amylopectin CLD is governed by at least four enzyme sets. In rice, the SL enzyme sets (i) and (ii), giving the SL CLD, appear to be substrate-competing; TL enzyme sets (iii) and (iv), giving the type-2 TL CLD, also appear to be substrate-competing. However, the SL enzyme sets are independent of the TL enzyme sets (see Results). In wheat, enzyme sets (i) and (ii) appear to be described by the independent substrate model while the rest are within the substrate-competing model, the same as in rice.

The modes of actions of the four enzyme sets quantitatively reproduce Features A–E in the rice amylopectin CLD (Figure 2). This methodology has also been applied with equal success to data for many other species and varieties (e.g. Figure S5B–D). This implies that there is no need to invoke further enzyme classes (i.e. other than SSs, SBEs and DBEs). Calculations show that Feature A is dependent on the values of *X*_0(i)_ and *X*_min(ii)_. It appears as a local maximum when the difference between *X*_0_ and *X*_min_ is great (Figure S6). The local maximum merges with the global maximum (Feature B) when the values of *X*_0(i)_ and *X*_min(i)_ are close (Figure S6). Feature A cannot be distinguished in rice amylopectin CLD (Figure 2), which is reflected in the fitted values of *X*_0(i)_ and *X*_min(ii)_ being close (Figure 4). Potato amylopectin CLD (Figure S5B) shows Feature A as a shoulder, and the difference in the fitted vaues of *X*_0(i)_ and *X*_min(ii)_ is greater (Figure S5). These results are consistent with the *in vitro* characteristics of the SBEs from both rice and potato. Rice SBEs generate a single peak in the product CLDs after reaction with linear glucans, while multiple peaks are observed with potato SBEs [14], [33]. The difference in the *X*_0_ and *X*_min_ is the cause of this behavior of peaks in the CLDs.

Even though either the substrate-competing or the independent substrate models yield good fits to rice amylopectin CLD (Figure 2 and S1), the substrate-competing model is favored because it is bound to produce a smooth Feature C, as seen in e.g. rice. The substrate-competing model also has one less parameter in the SL range (see Model section).

It is apparent that the type-2 TL chains come from independent TL enzyme sets. Within the TL range (*X* ≥32), however, a distinct Feature E from the TL enzyme sets (iii) and (iv) is not apparent (e.g. Figure 2, Figure S5 and Figure 3 in [24]). This is analogous to the smooth Feature C in rice. This favors the substrate-competing model for the type-2 TL CLD.

The substrate-competing and independent-substrate models are slightly different cases, and may be applicable to different ranges of CLDs and in different plant species. This suggests the following effects which have not been seen before. (1) Depending on the plant species, enzyme set (i) and (ii) may be described by either the substrate-competing or independent-substrate model. (2) A significant portion of the chains spanning beyond the SL range, the type-2 TL chains, arises from the TL kinetics, which are independent from the SL kinetics. The existence of these chains will be seen in a later section to suggest a new tool for biotechnology. (3) The enzyme sets (i.e (iii) and (iv)) governing the type-2 TL chains appears to be described by the substrate-competing model in all plant species studied.

It is also inferred from the good fit (Figure 2) that the contribution of primer glucans during the *de novo* synthesis of amylopectin molecules is insignificant for amylopectin CLD (i.e. **r** = 0 in Eqn 7), although the total mass of amylopectin changes in time, and this is governed by primer glucans.

**Figure 5:**
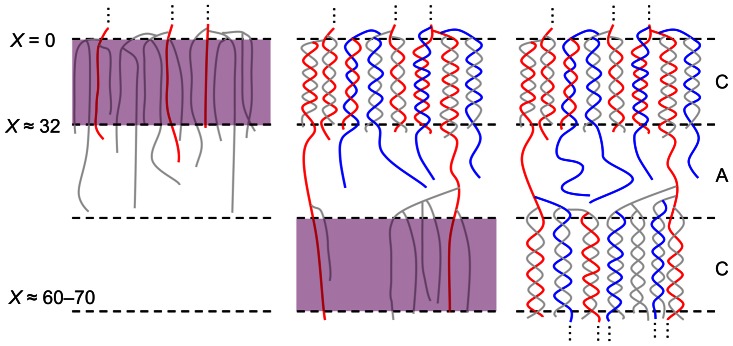
A proposed mechanism for the formation of the arrays of semi-crystalline lamellae in amylopectin. C and A indicate the crystalline and amorphous lamellae. The purple shaded regions indicate the nascent SL space where non-lamellar amylopectin chains, amorphous in nature, are being formed. Types of chains: (1) single-lamella (SL) chains that are (mostly) confined in crystalline lamellae (grey lines); (2) type-1 TL chains, crystalline-lamella-connecting chains, which span more than one crystalline lamellae (red lines); (3) type-2 TL chains, non-lamella-connecting chains, which protrude the SL space but remain in the amorphous lamellae (blue lines). The longest chain length confined to a crystalline lamella, predicted from Figure 2, is *X* ∼ 31 (i.e. chains ≥32 enters the contiguous amorphous lamella), while longer SL chains (red lines) with *X* ∼ 60–70 are sufficiently long to participate in crystalline formation in the subsequent crystalline lamella.

The amylopectin CLD is effectively described by the enzymatic actions (i.e. **N**_de_ = **N**_de.NL_ in Eqn 7) in a steady state (d**N**_de_/dt = 0 in Eqn 7), where crystallization does not influence d**N**_de_/d*t* (**F_cryst_** = 0 in Eqn 7). The mechanism for the steady-state amylopectin CLD formation is envisaged as follows. At the surface of a growing starch granule, the steady-state SL kinetics govern the amylopectin chains in the nascent SL space; these nascent chains are not yet arranged into a crystalline structure. Chains confined to the nascent SL space, amorphous in nature (Figure 5), are referred to as the non-lamellar chains. This is not to be confused with chains that reside in the amorphous lamellae in a grown starch granule. Crystallization of glucan chains is a series of complex physical processes. Deriving the exact expression for the rate of crystallization would require knowledge of both crystallization kinetics between the length and distance of the chains forming an *α* helix with another chain, and a knowledge of influences and interactions from other chains. Once crystallization occurs, the CLD is “frozen”: not susceptible to significant enzyme-induced changes. It is assumed that crystallization of the non-lamellar CLD proceeds in a non-selective manner with respect to the length of chains *X*. In other words, a chain of any length has the same chance of being frozen in the crystalline structure. It is argued that this freezing effect applies not only to the chains that are actually involved in *α*-helices, but also to the chains that reside in the amorphous lamellae. The latter are governed by the steady-state TL kinetics while a starch granule is growing, and become entrapped beneath layers of crystalline lamellae in a fully formed starch granule (Figure 5, where the amorphous lamella is sandwiched by two established crystalline lamella). Altogether, the crystallized chains and the frozen chains in the amorphous lamellae make up the steady-state amylopectin CLD in granular starch. This means the amylopectin CLD is simply that of the steady-state non-lamellar CLD and the steady-state TL CLD, which are governed by the enzymatic action, but then frozen irrespective of chain lengths in the lamella structure of starch granules. These conclusions are supported by the close fit of the model, which was developed for the CLD prior to crystallization, to amylopectin CLD in rice (Figure 2) and other species and varieties.

Not all possible steady-state CLDs may be crystallization-competent. For example, Figure 5 of Ref. [24] showed a steady-state CLD can be generated with a large value of *β* >1, but this yields a drastically different CLD to that observed in nature. We have performed fitting of a large number and variety of CLDs from the literature and always found *β* ≪ 1. The steady-state amylopectin CLD is a well established feature of granular starches. However, this may or may not apply to all stages of granule development or the growth of individual starch granules. For example, the amylopectin CLD at an early developmental stage of maize endosperm shows a different CLD to that of the later stages [34]. The amylopectin CLDs in A- and B-granules of wheat, which are synthesized at different developmental stages of the grain, are also different, but only slightly so [35]; amylopectin CLDs in developing barley grains do not show significant differences [31]. Amylopectin in the core of maize [36] and potato [37] starch granules contain more long chains compared to the periphery. Nevertheless, these discrepancies are minor in terms of the CLD ensemble from all of the starch granules developed at different time in cereal endosperms. Systematic study using the model to fit these differences in CLDs may provide insight into the biosynthetic pathway at different developmental stages of grains and different size granules.

The amylopectin CLD also varies with the starch-accumulating species, implying that there is a range of CLDs (almost certainly associated with an appropriate distribution of spacing between branch points) which are crystallization-competent. This may be related to the forms of crystallization contribution, **F**_cryst_, in a way which we do not yet understand.

Both SL and TL kinetic parameters have restrictions for a steady-state amylopectin CLD to form: the zero-eigenvalue requirement, as discussed in the Model section. The model implies that if either of SL or TL kinetic parameters do not obey these restrictions, the CLD will either proliferate indefinitely or disappear in time: a steady-state CLD will not be attained.

The steady-state condition provides a mathematical proof that precisely balanced ratios of the enzymatic activities are one of the requirements for the synthesis of an amylopectin CLD which is crystallization-competent. This is an independent confirmation of the conclusion, previously reached solely on the basis of genetic studies, of the absolute requirement for debranching enzyme in crystalline amylopectin synthesis (e.g. [2], [20], [38], [39], [40]). Loss of relative debranching activities will shift the parameters away from the steady state restrictions needed for crystallization-competent amylopectin CLD to form. We propose that this consequence of the model is entirely consistent with, and provides an explanation for, the experimental observation that in the complete absence of isoamylase-debranching enzyme activity, non-crystalline phytoglycogen is accumulated in the cereal endosperm [20], [38].

**Figure 6:**
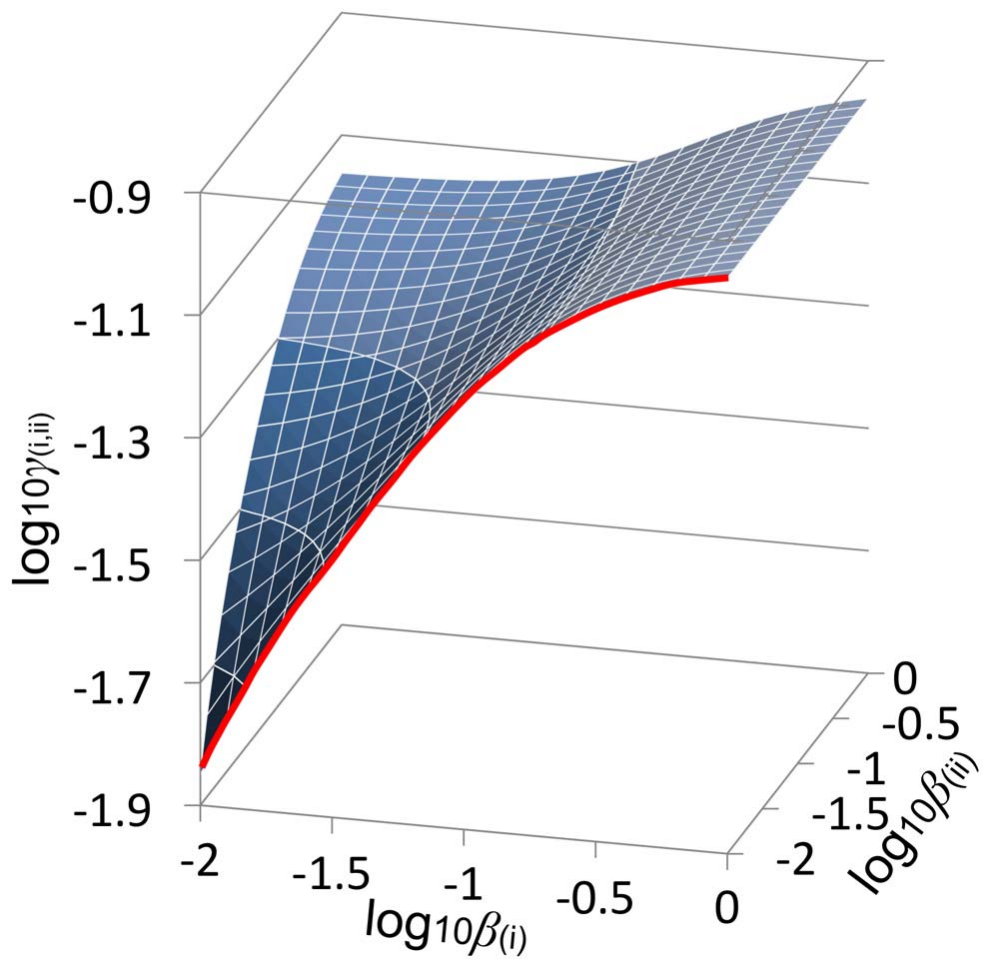
The steady-state surface, which describes the restrictions on the parameters for amylopectin CLD to form. Surface generated for *X*_0(i)_, *X*_min(i)_, *X*_0(ii)_ and *X*_min(ii)_ = 6, 7, 9 and 14 respectively. These values are the fitted parameters for Figure 2. The thick red line is an approximation to the steady-state line in our previous development (Figure 4 in [24]), where the exact steady-state line is when *β*_(ii)_ (as defined in Figure 4) = 0. *γ*_(i,ii)_ is the sum of relative debranching activities from set (i) and (ii) to that of propagation from set (i) and (ii).

It is apparent from the steady-state surface showing relations between possible ratios of enzymatic activities (see Model section and Figure 6) that there is a less steep change of the DBE ratio *γ*_(i,ii)_ with respect to the SBE ratio *β*_(ii)_ than with *β*_(i)_: the steady state is less sensitive to *β*_(ii)_. This is consistent with the fact that enzyme set (i), which creates most of the short chains in amylopectin, is important for crystalline formation. The weaker *β*_(ii)_ dependency may explain why the appearance of Feature C (Figure 2) varies appreciably among different starch-accumulating plants (e.g. rice [41]) and wheat (e.g. [42]), noting that enzyme sets (i) and (ii) may be either substrate-competing or independent. The variation is also seen within species (e.g. rice [41]). It is speculated that enzyme set (ii) (i.e. SS(ii), SBE(ii), DBE(ii)), rather than being genetically defined by particular isoform of enzymes, might be a biochemically defined machinery through processes such as phosphorylation, enzyme complexation, or other regulatory mechanism [5]. These processes are likely to be less consistent across species and varieties, and also subject to the influence of environmental factors.

### Inferences from the Fitted Parameters

One of the reductionist assumptions in the theoretical development is that enzymatic activities are represented by overall average rates (see the Model section). Now, chain-length-dependent activities certainly exist: Commuri and Keeling [43] have, for example, shown that SSI has different specific activity towards different chain lengths. However, there are insufficient data and as yet unresolved complexities in understanding the biosynthetic process. Examples of the concerns with respect to data availability are: (1) data are not available for all SS isoforms; (2) the relative activity of isoforms changes over time during organ development; (3) the available data are based on isolated enzymes in dilute solutions rather than at a soluble/insoluble interface zone; (4) the phosphorylation status of the enzymes is not yet known or described; (5) no account has yet been taken of possible conformation changes that may occur as part of enzyme complexation. Thus we consider that at this time it is more appropriate to use the reductionist assumption rather than incomplete data which may be subject to revision as the field matures. Once data to address the unknown parameters become available, extensions of the model can be made to include those. The fitted parameters, at present, may not reflect the true ratios of enzymatic activities; however, qualitative comparisons can be made between them.

The fitted parameters (Figure 4A) show the relative branching activities for the type-2 TL chains are lower than those of the SL. This is because long chains are relatively abundant in the type-2 TL CLD (Figure 3B). Lower relative branching activities means higher rate of chain elongation, and thus more long chains. The steady-state condition requires that lower *β* values in the TL kinetics must be accompanied by a lower relative debranching activity than in SL (Figure 6). It is reasonable to assume that this is because the branching points of the type-2 TL chains, embedded in the preceding crystalline lamella, are less accessible to DBEs.

The shortest *X* that SBEs can produce is *X* = 6, as determined by *Arabidopsis* leaves pulse-chased with ^14^CO_2_ [16]. Rice (e.g. Figure 2) and many other plant species (e.g. Figure S5) also show the shortest possible amylopectin chain in significant abundance is *X* = 6. This is consistent with the fitted value *X*_0(i)_ for rice (Figure 4B). There is no definite way of distinguishing if *X* = 6 from the references above represents that of *X*_0_ or *X*_min_ at this stage. Related experiments often examine the final CLD formed by subjecting glucan polymers to SBEs, and there is at present no unambiguous way to determine the transferred glucan minimum DP (*X*_min_) from that for the remaining glucan (*X*_0_).

The quantity giving the relative abundance of type-2 TL chains, *h*_(iii/i)_, is ∼10^–2^ for rice (Figure 4A), with small values also obtained for many other species (Fig. S5). This implies that the contribution from enzyme sets (iii) and (iv), giving the type-2 TL chains that reside in the amorphous lamellae, is relatively small compared to the SL chains. This may be because the majority of SL chains, when aligned into crystalline lamella, are less likely to be elongated further. It is also possible that the physical environment of the amorphous lamellae may obstruct growth of the type-2 TL chains due to steric hindrance. Further values for *h*_(iii/i)_ were found by treating the CLDs obtained in [25] with the model (Figure S5 bottom panel). The resulting values *h*_(iii/i)_ are correlated with the botanical backgrounds: higher for storage starches in dicots (B-type) than monocots (A-type). High-amylose maize (B-type) shows elevated abundance of type-2 TL chains compared to normal maize (A-type). Together, these show the variability of *h*_(iii/i)_, which may be associated with the types of crystal forms. A higher *h*_(iii/i)_ is found for potato starch (B-type), which is consistent with its amorphous lamellae being denser (filled with more glucan materials) compared to the A-type starches, a conclusion based on the electron density profiles of stacked lamellae [44].

### Formation of Successive Arrays of Crystalline Lamellae in the Semi-crystalline Layers of Starch Granules

Fitting various data sets with the substrate-competing model implies that long chains, 32≥ *X* 60–70 for the particular case fitted in detail here, comprise two types: types-1 and -2 TL chains. The former, being involved in crystalline lamellae, is governed by the SL enzyme sets (i.e. SS(i–ii), SBE(i–ii) and DBE(i–ii)). The only possible way for this to occur is that longer chains (*X* ≥32) originate from a crystalline lamella, protrude from that lamella, span across the contiguous amorphous lamella, and are then able to evolve further under SL kinetics and participate in crystalline formation in the subsequent crystalline lamella (Figure 5, red lines). The type-1 TL chains, connecting crystalline lamellae, correspond to the crystalline-lamellar connecting chains in the cluster model of amylopectin (e.g. [17]).

Type-2 TL chains are governed by the TL enzyme sets (i.e. SS(iii–iv), SBE(iii–iv) and DBE(iii–iv)) which are independent of the SL enzyme sets. They are likely to be produced when some of the long SL chains protrude from their parent crystalline lamella. It is envisaged that the crystalline lamella is established to a degree where the biosynthetic enzymes can no longer “sense” the glucans in the crystalline structure. It is then assumed that the protruding portion of these long SL chains behaves like short SL chains again. This assumption is taken into account in the model by displacing the starting *X* of the type-2 TL CLD to *X* = 1 when fitting (see Model section). The type-2 TL chains evolving under the TL enzyme sets are suggested to remain in the amorphous lamellae (Figure 5, blue lines), which can explain the different parameters in for the type-2 TL chains (Figure 4A). The existence of type-2 TL chains has not been distinguished previously in the cluster model of amylopectin. They are ∼ 2.2 times more numerous than the type-1 TL chains for the particular case fitted in detail here (the total number of chains of type-2 to type-1 TL chains for 32≥ *X* 60–70).

The formation of successive arrays of crystalline lamellae is envisaged as follows. Consider a randomly branched nascent SL space at the periphery of a growing starch granule. The CLD confined to this space is governed by the SL kinetics in a steady state. To facilitate crystallization, “improperly positioned” chains are removed by the action of DBEs. While chain removal is occurring, a steady-state CLD is always maintained because there are contributions from the SSs and SBEs with rates in a steady-state with that of the DBEs. For chains in the nascent SL space to crystallize, a steady-state CLD must be attained; other factors may include the branch spacing, which has been proposed to facilitate crystallization (e.g. [3], [19]). Crystallization freezes a distribution of chain lengths in the non-lamellar phase and in the amorphous lamellae beneath in a growing starch granule non-selectively (discussed earlier). This gives the steady-state amylopectin CLD in granular starch. Some longer SL chains (*X* ≥32) protrude their originating crystalline lamellae, are captured in the materializing subsequent crystalline lamella, and evolve to type-1 TL chains (Figure 5). The rest of the long SL chains remain in the contiguous amorphous lamella and evolve under TL kinetics to form the type-2 TL chains, which is a new type of branch both in the amylopectin cluster model [17] and in the “two-step branching and improper branch clearing model” proposed by Nakamura [3].

The semi-crystalline structure in starch granules is produced by a repetitive synthesis of the crystalline and amorphous lamellae as described above. This implies that amylopectin chains form semi-crystalline structures progressively in layers, through successive lamellar formation.

### A New Parameterization Tool for Structure-property Relations

This CLD model provides a new parameterization, which reduces the amylopectin CLD to a small number of meaningful biosynthetic-based parameters (Figure 4) for comparisons between species and mutants. The actual parameters are *β*_(i)_, *X*_0(i)_, *X*_min(i)_, *β*_(ii)_, *X*_0(ii)_, *X*_min(ii)_, *β*_(iii)_, *X*_0(iii)_, *X*_min(iii)_, *β*_(iv)_, *X*_0(iv)_, *X*_min(iv)_ and *h*_(iii/i)_ (in practice, e.g. [45], not all of these are needed). This is an alternative to the widely used empirical approaches, such as difference plots, for comparisons between different CLDs (e.g. [3], [19], [20], [35], [42]).

It has been found (e.g. [45]) that this new parameterization provides an improved tool for a statistical identification of structurally important characteristics of a starch with regard to properties such as crystallinity and digestibility. This parameterization is physically based, and is able to represent the entire CLD accurately in terms of a few parameters; it thus encompasses all information in empirical representations such as difference plots.

### Insight into Starch Biosynthesis for Plant Biotechnology

The significant implication for plant biotechnology from this work is that two types of TL chains are distinguished in the substrate-competing model as for rice and their relative abundance, *h*_(iii/i)_, may not be restricted by a steady-state condition as for the SL and TL kinetic parameters. It is not clear what controls *h*_(iii/i)_. It is possible that the variability of this quantity is associated with the nature of crystal forms as discussed earlier. This points to the need for understanding the factors influencing the synthesis of type-2 TL chains in the amorphous lamellae for developing starch with elevated abundance of long chains. There is evidence that slowly digestible starch is positively correlated with abundance in long and intermediate chains, which may have nutritional benefits [46], [47]. Of course, this may not be the only mechanism for quality foods: e.g., resistant starch, which has considerable health benefits, can be obtained from different starches and structures and processing treatments [48].

The modeling approach set out in this paper has the potential to aid in the understanding of starch structures and their manipulations.

## Model

The current paper gives three major advances on the earlier model which improve the model starch biosynthesis. (1) The present model takes into account de novo initiation of amylopectin molecules from glucan primers, synthesis of branched molecules and alignment of chains into crystalline structure. (2) The model takes into account, if appropriate for the plant species under study, that SL chains can be governed by the substrate-competing actions of enzyme set (i) and (ii) (i.e. SS(i–ii), SBE(i–ii), DBE(i–ii)). For substrate-competing enzyme sets, the rate equations include all the contributions from both enzyme sets in an overall time evolution equation (e.g. Eqn 1). This means the substrates are susceptible to either of the enzyme sets. As a result of this treatment, the relative abundance of the CLDs, governed by enzyme set (i) and (ii), does not need to be specified. This reduces the model parameters by one. The substrate-competing model is an alternative to our previous independent substrate model [24] where the evolution equation for each enzyme set was treated independently and the relative abundance of the CLDs from enzyme sets and (ii) in relation to the CLD from enzyme sets (i) was required. (3) Michaelis-Menten kinetics are incorporated (Text S1).

The concentration (e.g. mol dm^–3^) of the non-lamellar CLD in the nascent SL space, assumed to be on the outermost surface of a starch granule, at time *t* is denoted 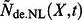. The non-lamellar CLD describes the chains that are not yet arranged in crystalline lamellae in starch granules, as opposed to lamellar CLD. The time evolution of the SL CLD, is given in Eqn 1 and derived in Text S1 (Eqn S1–S14).
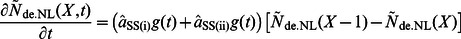


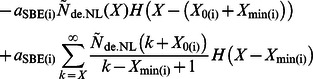


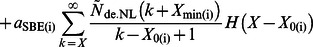


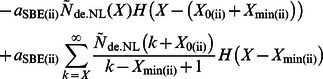


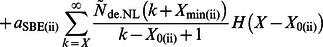


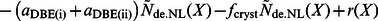
(1)


The rate of ADP-glucose addition is (*â*_SS(i)_ *g*(*t*)+*â*_SS(ii)_ *g*(*t*)) multiplied by the concentration of available non-reducing ends; *g*(*t*) is the ADP-glucose concentration (e.g. with units mol dm^–3^). The rate *â*_SS(i)_ is the time-independent part of the overall rate of ADP-glucose addition through SS(i) during elongation of glucan chains, with units of dm^3^ mol^–1^ s^–1^; *a*_SBE(i)_ is the rate at which branching proceeds by SBE(i) in enzyme set (i), with units of *s* ^–1^; *a*_DBE(i)_ is the rate for debranching with the same units as *a*_SBE(i)_. The rate of glucan materials incorporated into the overall CLD during the *de novo* synthesis of amylopectin is *r*(*X*), with units of mol dm^–3^ s^–1^. *f*_cryst_ is the rate (units of s^–1^) at which the non-lamellar chains with DP *X* crystallize driven by physical rather than enzymatic processes. *H*(*y*) is a step function: *H*(*y*) = 0 for *y* <0, = 1 for *y* ≥0, and appears because of various constraints (i.e. *X*_0_ and *X*_min_) on SBEs (Figure 1 and Eqns S4–6). All enzymatic activities are assumed time- and chain-length independent except for the time-dependent ADP-glucose concentration *g*(*t*) and the effects of *X*_0_, *X*_min_ for SBEs. Activities are overall average rate parameters (e.g. *â*_SS(i)_). These average rate parameters specifically include the Michaelis-Menten rate coefficients (Text S1).

The rate *â*_SS(i)_ *g*(*t*)+*â*_SS(ii)_ *g*(*t*) is factored out from the right-hand side of Eqn 1 in terms of the quantities:
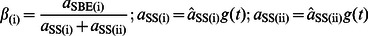
(2)

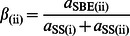
(3)

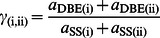
(4)


Define a vector 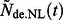 whose elements comprise the 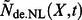 and, likewise, a vector **r** for *r*(*X*). Eqn 1 is converted to matrix notation in Eqn 5.
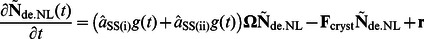
(5)


Here the matrix **Ω** has elements comprising the rate of change of the vector 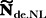 as given in the right hand side of Eqn 1; these elements are the contributions of enzyme set (i) and (ii). **F**_cryst_ is a diagonal matrix giving the crystallization contribution.

### Derivation of the Steady-state Solution

The concentration of the lamellar CLD, 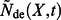, is determined by the rate of crystallization of 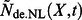. Once the chains are arranged into lamellar structure in starch granules it is assumed that they are “frozen” and thus not susceptible to significant enzyme-induced changes [49]. Thus the time evolution of the absolute concentration of the lamellar CLD is given by:
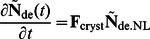
(6)


The lamellar CLD is approximated as independent of time as discussed in the Introduction. *N*_de_(*X*) (the relative number of chains) is related to 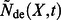 (the actual concentration of chains) by:
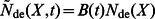
where *B*(*t*) is related to the overall rate of synthesis/degradation of starch in time.

The rate of change of the SL lamellar CLD is then, in matrix notation:
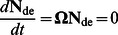
(7)


Eqn 7 is derived from Eqn 5, which describes the evolution of the SL CLD, by considering that the rate of change of the SL CLD in cereal endosperms, governed by the two SL enzyme sets (i.e. SS(i–ii), SBE(i–ii) and DBE(i–ii)), is at a steady state (i.e. the right hand side of Eqn 5 = 0); putting **r** = 0; **F**_cryst_ = 0; **N**_de_ = **N**_de.NL_; and dividing Eqn 5 by *â*_SS(i)_ *g*(*t*)+*â*_SS(ii)_ *g*(*t*). These assumptions, necessary for solving for **N**_de_, explained in the discussion, give a mechanism for the attainment of the steady-state lamellar CLD.

Eqn 7, a system of infinite linear equations, has the solution (see e.g. [24]) (Eqn 8):
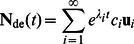
(8)

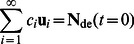
(9)where λ*_i_* is the *i* ^th^ eigenvalue of **Ω** and **u** *_i_* is the corresponding eigenvector; *c_i_* is obtained by solving the appropriate relation (Eqn 9) as a system of simultaneous equations. **N**_de_(*t* = 0) is a vector whose elements comprise all the elements of *N*_de_(*X*, *t* = 0), which give the lamellar CLD at the beginning of the period in question. Practically, *i* is truncated at a sufficiently large value (∼110) so that **N**_de_(*t*) converges to within a desirable tolerance.

The solution of Eqn 8 is dictated by the SL kinetic parameters: *β*_(i)_, *X*_0(i)_, *X*_min(i)_, *β*_(ii)_, *X*_0(ii)_, *X*_min(ii)_ and *γ*_(i,ii)_. The only way that a unique steady-state CLD is obtained at long times is if one eigenvalue is exactly zero and all others are negative. If one or more eigenvalues are positive, the CLD grows indefinitely, while the contributions from the negative eigenvalues disappear in a short induction time, leaving that for the zero eigenvalue. The steady-state CLD is then given by the eigenvector corresponding to the zero eigenvalue.

The steady-state SL CLD restricted to only a particular set of SL parameters which are conveniently presented by a *steady-state surface* for a given set of values of *X*_0(i)_, *X*_min(i)_, *X*_0(ii)_ and *X*_min(ii)_ (Figure 6). This surface gives *γ*_(i,ii)_ as a function of *β*_(i)_ and *β*_(ii)_, which then excludes the need to have *γ*_(i,ii)_ as an independent parameter: *γ*_(i,ii)_ is a function of *β*_(i)_, *β*_(ii)_, *X*_0(i)_, *X*_min(i)_, *X*_0(ii)_ and *X*_min(ii)_. The surface depends on the values of the various *X*_0_ and *X*_min_, but the basic shape of the surface is retained. The steady-state surface is analogous to the “steady-state line” depicted in Figure 4 of our previous paper [24], but with one extra dimension: *β*_(ii)_. The slice of the surface at *β*_(ii)_ = 0 gives the previous steady-state line. Any point on the steady-state surface gives a set of eigenvalues (Eqn 8) comprising exactly one zero eigenvalue and the rest negative.

### Method of Fitting the Model to Experimental CLDs

A freeware Fortran program for least-squares fitting both the substrate-competing and independent substrate models to amylopectin CLD in cereal endosperms such as rice, implementing the mathematical treatment development given above, is available for download (Text S3, S4 and [50]). A detailed step-by-step guide and examples of fitting (Figure S7, S8, S9, S10, S11) are also provided.

Fitting is carried out by non-linear least squares fitting of Eqn 8 to the SL range of the experimental CLD with this Fortran program. As apparent from Figure 3A, SL chains alone do not reproduce the Features D and E, as discussed in the Results section. The calculated SL CLD is subtracted from the experimental CLD and the difference, referred to as the type-2 TL CLD. In order to treat the type–2 TL CLD the starting *X* of the type-2 TL CLD (*X* = 32 in rice amylopectin CLD (Figure 3A)) is displaced to *X* = 1 and then fitted as described for the SL CLD. The implication of the displacement treatment is given in the Discussion. It is assumed that the type-2 TL chains are governed by two TL enzyme sets (i.e. SS(iii–iv), SBE(iii–iv) and DBE(iii–iv)) acting in a substrate-competing manner, analogous to enzyme sets (i) and (ii), in a steady state.

The ratio of the maximum of the type-2 TL CLD to that of the SL CLD in the substrate-competing model is given by the quantity *h*_(iii/i)_. This is found by subtracting the calculated SL CLD, with a maximum of *h*_(i)_, from the experimental CLD and dividing the maximum of the calculated type-2 TL CLD, with a maximum of *h*_(iii)_, by *h*_(i)_. For convenience the SL CLD is always normalized to a maximum of 1 (i.e. *h*_(i)_ = 1). The ratio of *h*_(iii)_ to this *h*_(i)_ is referred to as *h*_(iii/i)_.

The overall fitting is obtained by adding the calculated SL CLD (normalized to a maximum of 1) and the calculated type-2 TL CLD (removing the displacement in *X*) normalized to a maximum of *h*_(iii/i)_. This is given by:
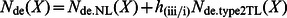



## Supporting Information

Figure S1 **Rice amylopectin CLD fitted with the independent substrate model.** This figure is analogous to Figures 2 and 3 combined in the article. Experimental CLD is taken from Figure 2. Yellow circles: experiment.
(TIF)

Figure S2 **Wheat amylopectin CLD (yellow circles) fitted with the independent substrate model.** This figure is analogous to Figures 2 and 3 combined in the article. Yellow circles: experiment. The fitting is similar to, but better than, that used for the same data in our earlier work [24].
(TIF)

Figure S3 **Calculated CLD (red circles) when three enzyme sets act in a substrate-competing manner.** Rice amylopectin CLD (yellow circles; experiment) is from Figure 2. Black-, gray- and white-filled shapes indicate *X* = 6, 30, and 67, respectively. Experiment shows a pronounced shoulder/maximum around *X* = 40 (indicated by the top arrow) while the calculated CLD shows a barely visible feature (indicated by the lower arrow). *X*_min(iii)_ and *X*_0(iii)_ are 40 and 4, respectively. A value of 0.054 is used for *β*_(iii)_, which is the average *β* for the trans-lamella kinetics (Figure 4; *β*_(iii)_ and *β*_(iv)_).
(TIF)

Figure S4 **Parameters yielded by the substrate-competing (black bars) and independent substrate model (gray bars).** These parameters are for fitting the single-lamella range of the CLD in Figure S3. In the substrate-competing model, *β* is the branching activity from an enzyme set divide by that of the total propagation from set (i) and (ii). In the independent model, *β* is the branching activity divided by propagation activity from only one enzyme set. The value *h*_(ii/i)_ is not applicable in the substrate-competing model.
(TIF)

Figure S5 **CLD from various botanical backgrounds fitted with the substrate-competing model.** Calculated CLD (green circles) fitted to the experimental amylopectin CLD of (A) wheat, (B) potato, (C) normal maize, and (D) high amylose maize. (E) shows the fitted *h*_(iii/i)_ values from (A)–(D). The amylopectin CLD, obtained by FACE, was digitized from ref. [25]. *X*_0(i)_ and *X*_min(i)_ values used for fittings are: (A) 2, 10; (B) 6, 10; (C) 6, 7; (D) 7, 9.
(TIF)

Figure S6 **Calculated CLDs (red circles) with a combination of different values of** ***X*** **_0(i)_ and** ***X*** **_min(i)_.** Rice amylopectin CLD (experimental; yellow squares, data from Figure 2) is given as a reference for the calculated CLDs. A combination of a range of *X*_0(i)_ and *X*_min(i)_ values are used to generate the calculated CLDs: *X*_0(i)_ of 3, 4, 5, and 6 (rows: top to bottom); *X*_min(i)_ of 7, 8, 9 and, 10 (columns: left to right). *X*_0(ii)_ and *X*_min(ii)_ of 9 and 14 is used throughout and does not influence the global maximum significantly. Black- and gray-filled shapes are *X* = 6 and 30, respectively.
(TIF)

Figure S7 **Description of the features in rice amylopectin CLD (yellow circles).** Experimental CLD is reproduced from Figure 2. *X* stands for DP. Crosses mark the features of the CLD. Feature A is due to *X*_0(i)_ of SBE(i). Feature B is the maximum which appears between *X*_0(i)_ and *X*_0(i)_+*X*_min(i)_ of SBE(i). Feature C is a small bump arising from the *X*_0(i)_ and *X*_min(ii)_ restriction on SBE(ii) in the same way as *X*_0(i)_ and *X*_min(i)_. Features A, B, and C, ascribed to enzyme sets (i) and (ii), are for chains confined to single lamellae (SL), where the chains pack together forming crystalline lamellae. The SL chains dominate the range 6≤ *X* ≲ 30. Chains protruding the SL range enter the immediate amorphous lamella, here termed the trans-lamella (TL) range. Features D and E are analogous to Features B and C, except that they are in the TL range (ascribed to enzyme sets (iii) and (iv)). The TL equivalent of Feature A is not apparent. Feature F indicates chains that span beyond a SL and TL. Being able to distinguish the equivalents of Features A, B, and C in the Feature F range requires a larger CLD range and more accurate data than are usually available.
(TIF)

Figure S8 **Preliminary fitting (green circles) to the CLD described in Figure S7 (yellow circles).** The fitting is generated with some initial guesses of the fitting parameters.
(TIF)

Figure S9 **Optimized fitting to the single-lamella range (DP≤30) of the CLD in Figure S8.**
(TIF)

Figure S10 **Optimized fitting to the trans-lamella range (DP>30) of the CLD in Figure S9.**
(TIF)

Figure S11 **Optimized fitting (green circles) to a typical amylopectin CLD (yellow circles) obtained by SEC.**
(TIF)

Text S1 **Derivation of the time-evolution equation for the solution of CLD.**
(PDF)

Text S2 **Type-2 TL chains are independent from the SL chains.**
(PDF)

Text S3 **Program “APCLDFIT” manual.**
(PDF)

Text S4 **APCLDFIT code.**
(PDF)
