# Supplementary figures and images for: A Parameterized Model of Amylopectin Synthesis Provides Key Insights into the Synthesis of Granular Starch
Source: PLoS One. 2013 Jun 7;8(6):e65768. doi: 10.1371/journal.pone.0065768 (PMC3676345; doi:10.1371/journal.pone.0065768)

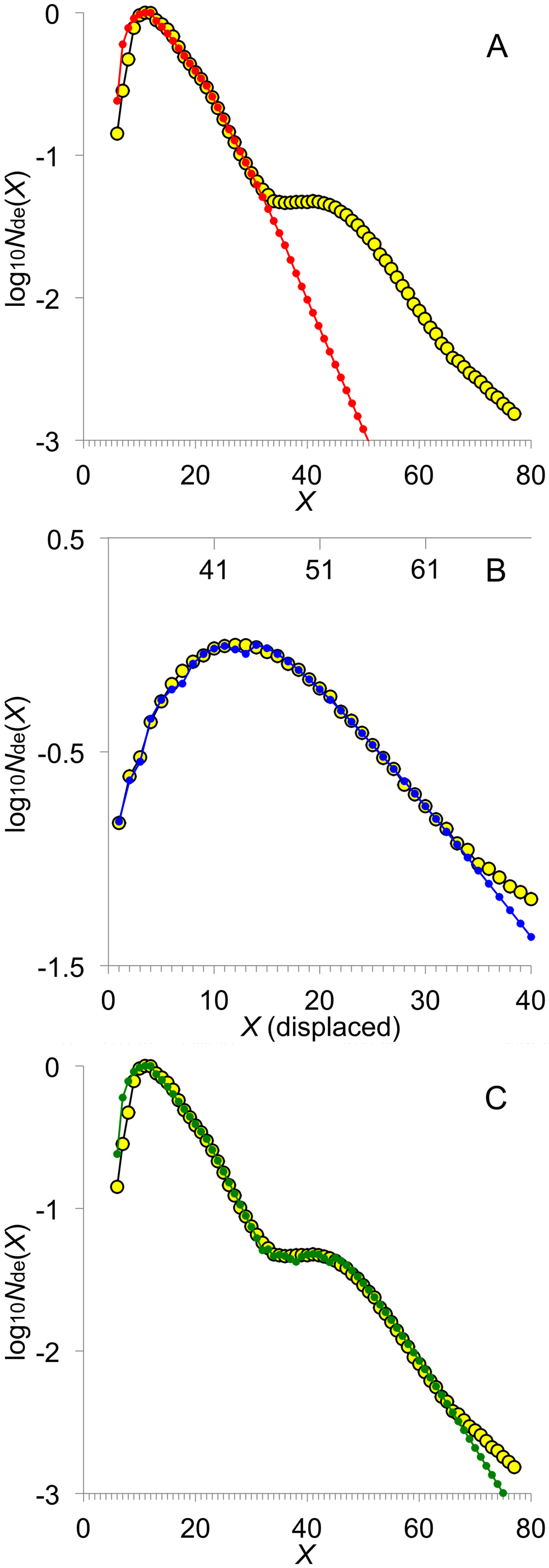

Supplement: Figure S1 — Rice amylopectin CLD fitted with the independent substrate model. This figure is analogous to Figures 2 and 3 combined in the article. Experimental CLD is taken from Figure 2. Yellow circles: experiment. (TIF) [file pone.0065768.s001.tif]

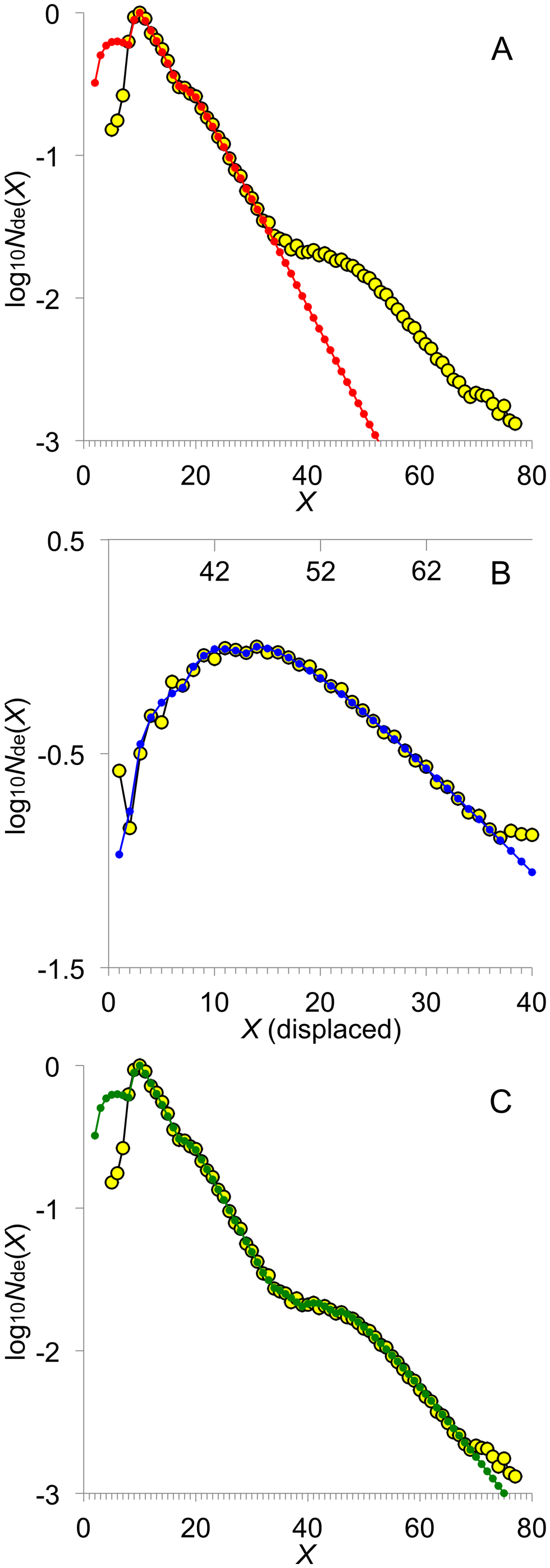

Supplement: Figure S2 — Wheat amylopectin CLD (yellow circles) fitted with the independent substrate model. This figure is analogous to Figures 2 and 3 combined in the article. Yellow circles: experiment. The fitting is similar to, but better than, that used for the same data in our earlier work [24]. (TIF) [file pone.0065768.s002.tif]

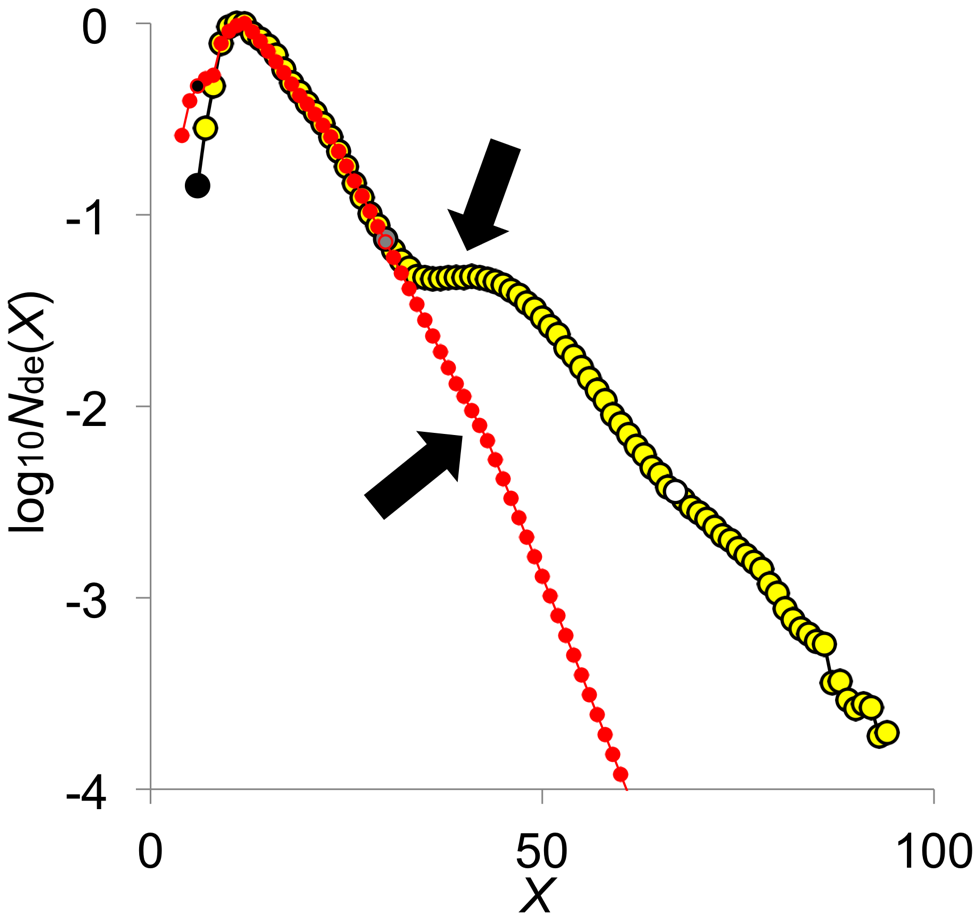

Supplement: Figure S3 — Calculated CLD (red circles) when three enzyme sets act in a substrate-competing manner. Rice amylopectin CLD (yellow circles; experiment) is from Figure 2. Black-, gray- and white-filled shapes indicate X = 6, 30, and 67, respectively. Experiment shows a pronounced shoulder/maximum around X = 40 (indicated by the top arrow) while the calculated CLD shows a barely visible feature (indicated by the lower arrow). X min(iii) and X 0(iii) are 40 and 4, respectively. A value of 0.054 is used for β (iii), which is the average β for the trans-lamella kinetics (Figure 4; β (iii) and β (iv)). (TIF) [file pone.0065768.s003.tif]

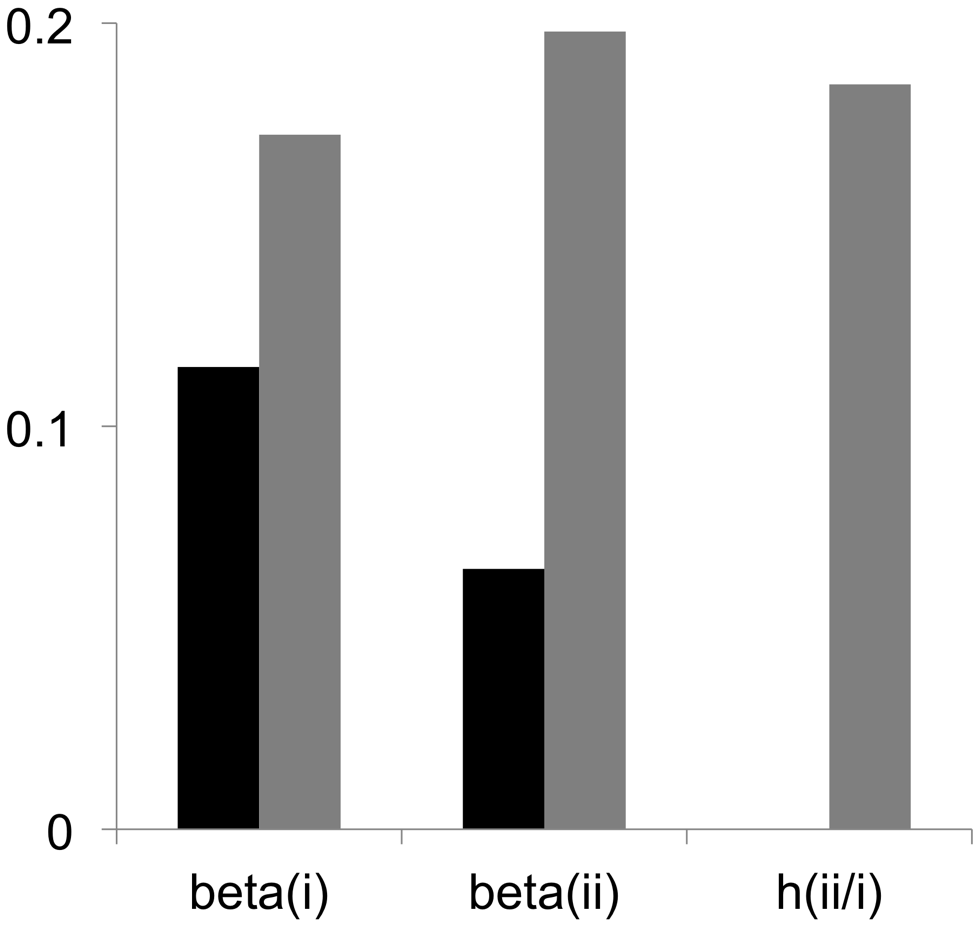

Supplement: Figure S4 — Parameters yielded by the substrate-competing (black bars) and independent substrate model (gray bars). These parameters are for fitting the single-lamella range of the CLD in Figure S3. In the substrate-competing model, β is the branching activity from an enzyme set divide by that of the total propagation from set (i) and (ii). In the independent model, β is the branching activity divided by propagation activity from only one enzyme set. The value h (ii/i) is not applicable in the substrate-competing model. (TIF) [file pone.0065768.s004.tif]

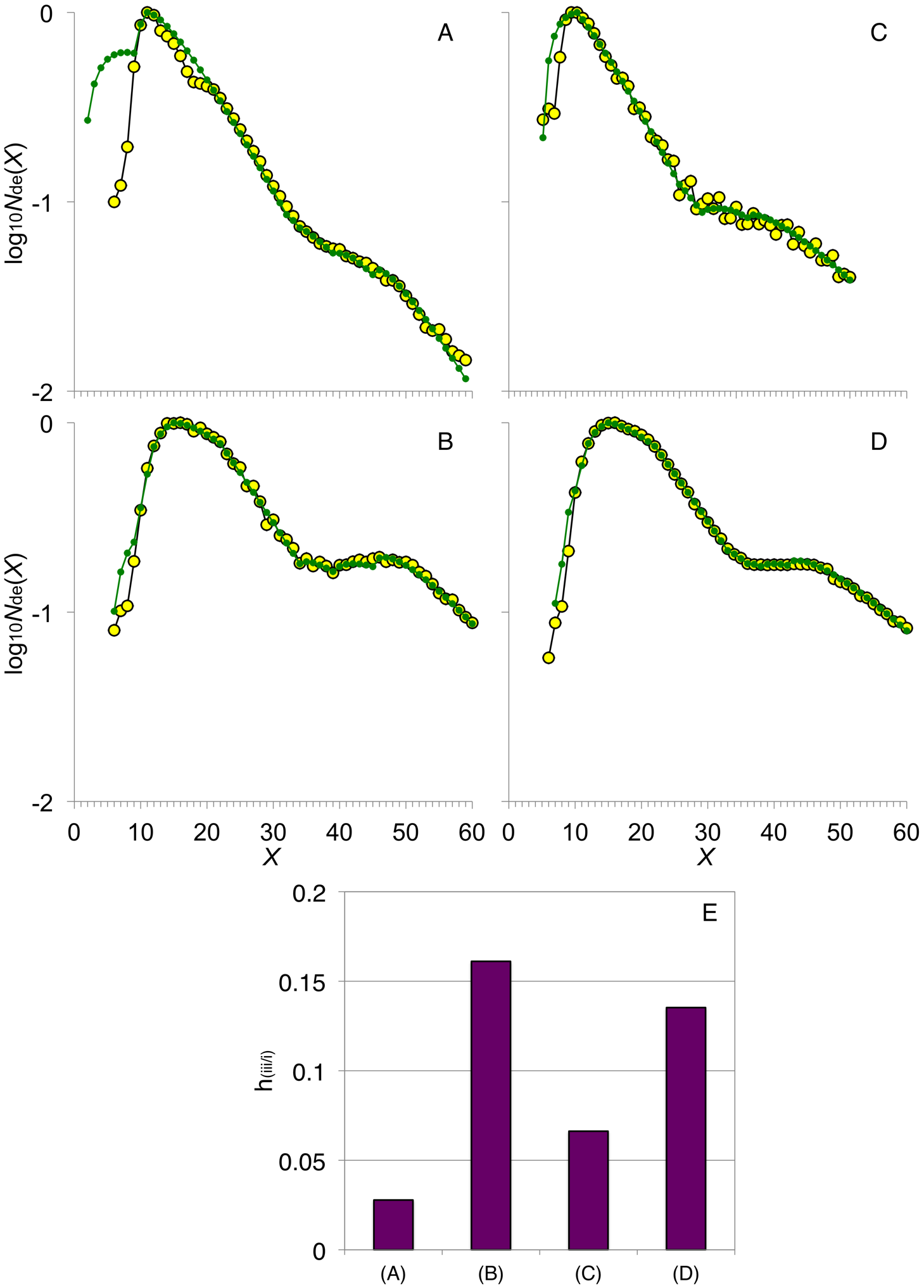

Supplement: Figure S5 — CLD from various botanical backgrounds fitted with the substrate-competing model. Calculated CLD (green circles) fitted to the experimental amylopectin CLD of (A) wheat, (B) potato, (C) normal maize, and (D) high amylose maize. (E) shows the fitted h (iii/i) values from (A)–(D). The amylopectin CLD, obtained by FACE, was digitized from ref. [25]. X 0(i) and X min(i) values used for fittings are: (A) 2, 10; (B) 6, 10; (C) 6, 7; (D) 7, 9. (TIF) [file pone.0065768.s005.tif]

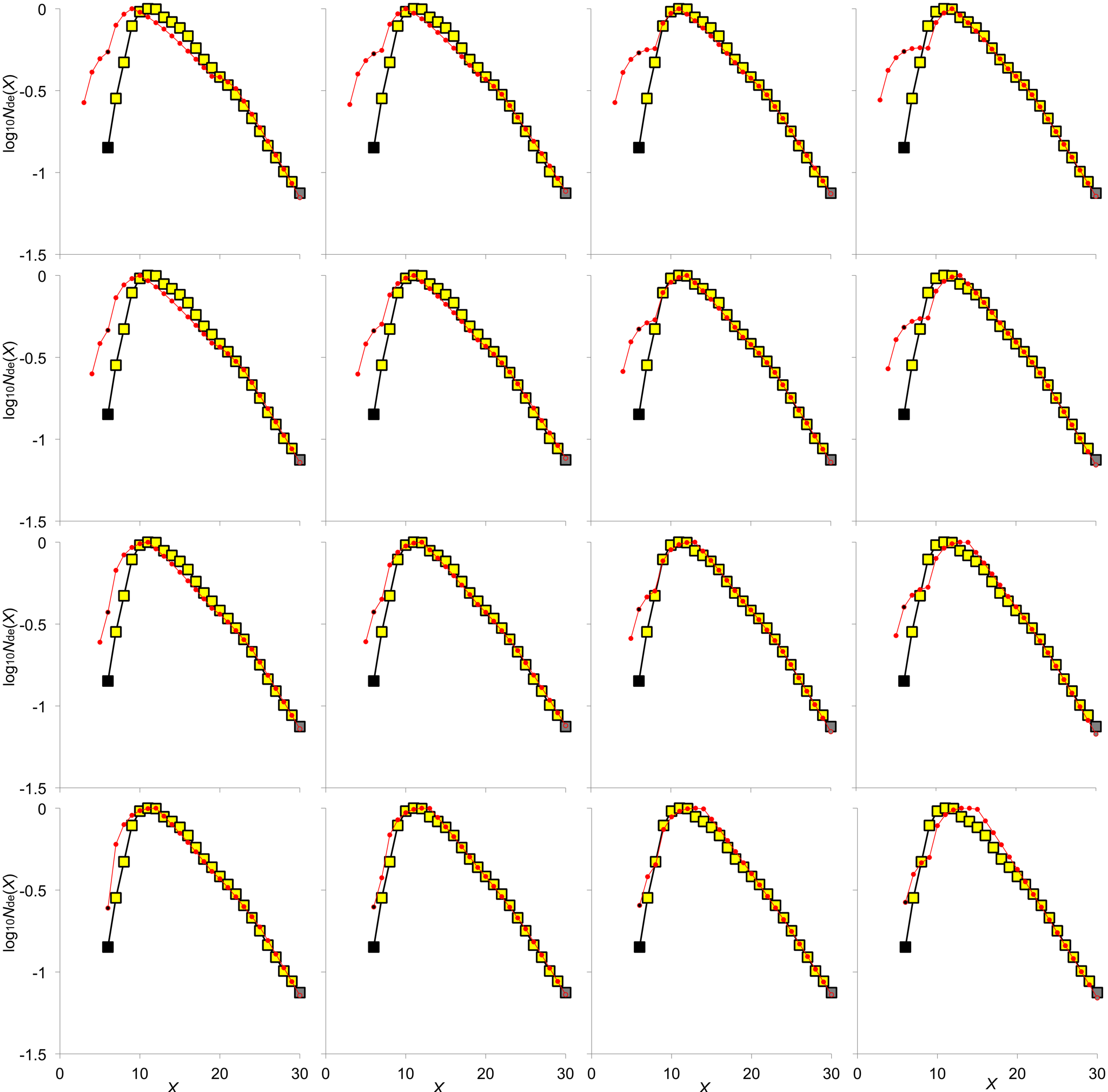

Supplement: Figure S6 — Calculated CLDs (red circles) with a combination of different values of X 0(i) and X min(i). Rice amylopectin CLD (experimental; yellow squares, data from Figure 2) is given as a reference for the calculated CLDs. A combination of a range of X 0(i) and X min(i) values are used to generate the calculated CLDs: X 0(i) of 3, 4, 5, and 6 (rows: top to bottom); X min(i) of 7, 8, 9 and, 10 (columns: left to right). X 0(ii) and X min(ii) of 9 and 14 is used throughout and does not influence the global maximum significantly. Black- and gray-filled shapes are X = 6 and 30, respectively. (TIF) [file pone.0065768.s006.tif]

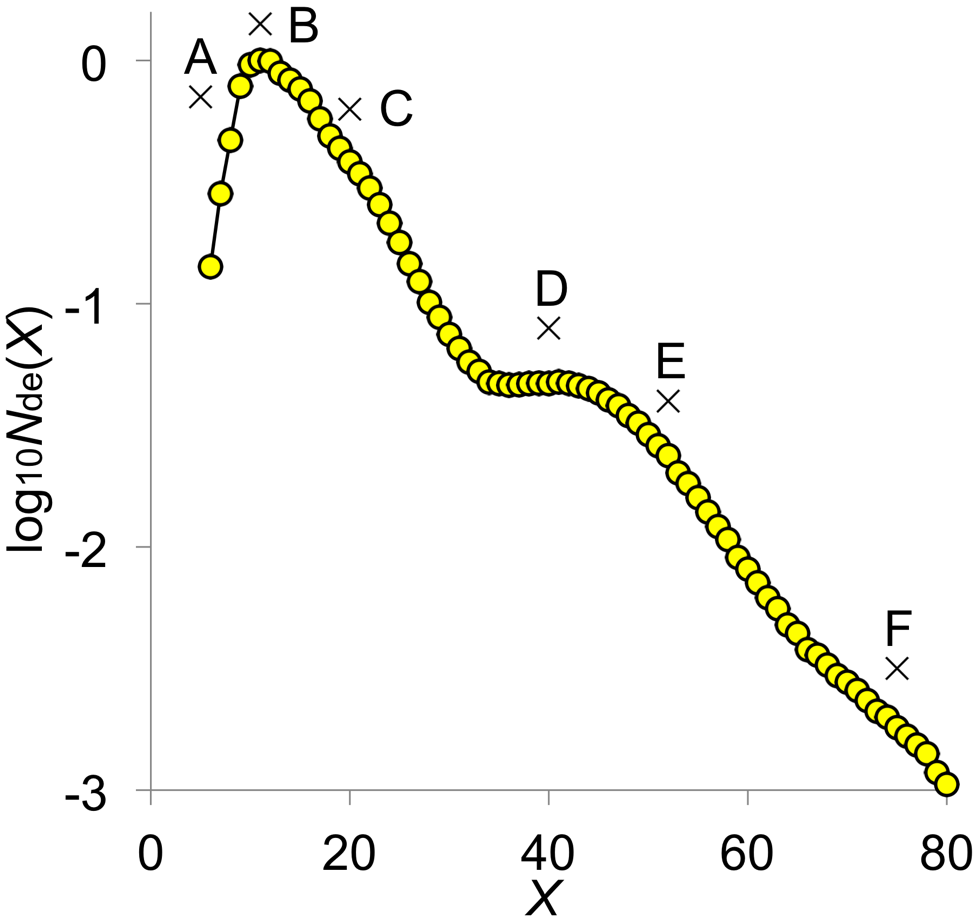

Supplement: Figure S7 — Description of the features in rice amylopectin CLD (yellow circles). Experimental CLD is reproduced from Figure 2. X stands for DP. Crosses mark the features of the CLD. Feature A is due to X 0(i) of SBE(i). Feature B is the maximum which appears between X 0(i) and X 0(i)+X min(i) of SBE(i). Feature C is a small bump arising from the X 0(i) and X min(ii) restriction on SBE(ii) in the same way as X 0(i) and X min(i). Features A, B, and C, ascribed to enzyme sets (i) and (ii), are for chains confined to single lamellae (SL), where the chains pack together forming crystalline lamellae. The SL chains dominate the range 6≤ X ≲ 30. Chains protruding the SL range enter the immediate amorphous lamella, here termed the trans-lamella (TL) range. Features D and E are analogous to Features B and C, except that they are in the TL range (ascribed to enzyme sets (iii) and (iv)). The TL equivalent of Feature A is not apparent. Feature F indicates chains that span beyond a SL and TL. Being able to distinguish the equivalents of Features A, B, and C in the Feature F range requires a larger CLD range and more accurate data than are usually available. (TIF) [file pone.0065768.s007.tif]

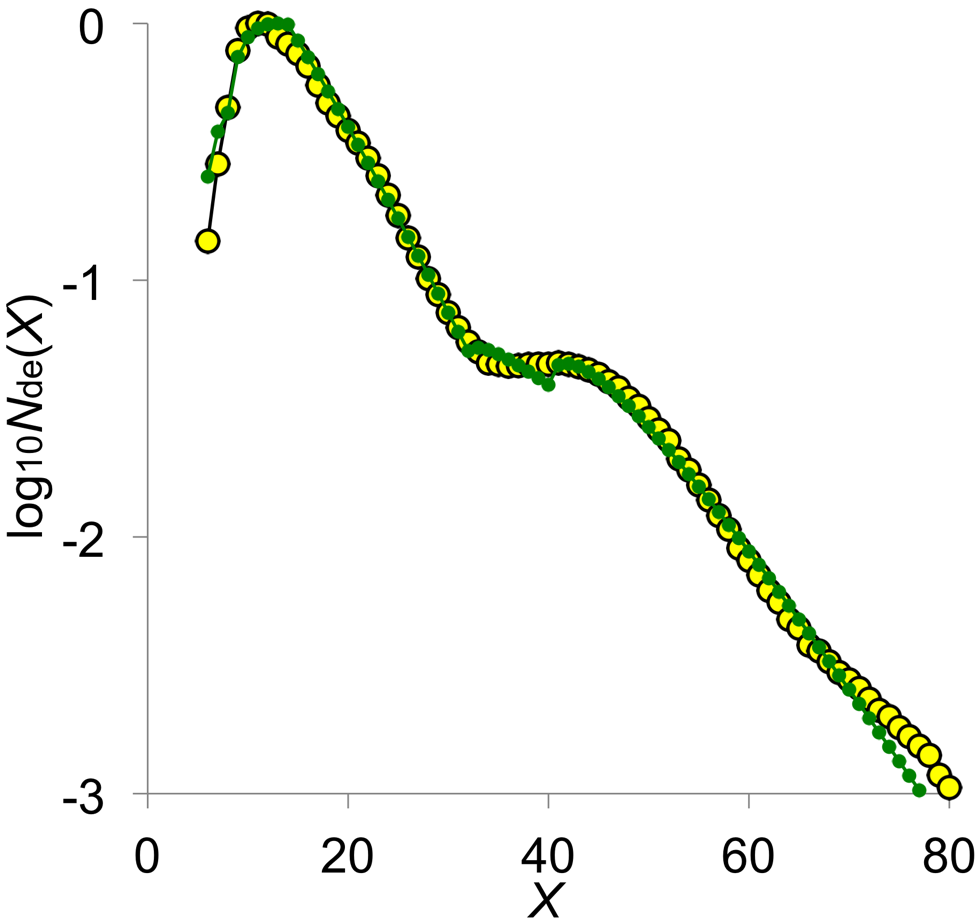

Supplement: Figure S8 — Preliminary fitting (green circles) to the CLD described in Figure S7 (yellow circles). The fitting is generated with some initial guesses of the fitting parameters. (TIF) [file pone.0065768.s008.tif]

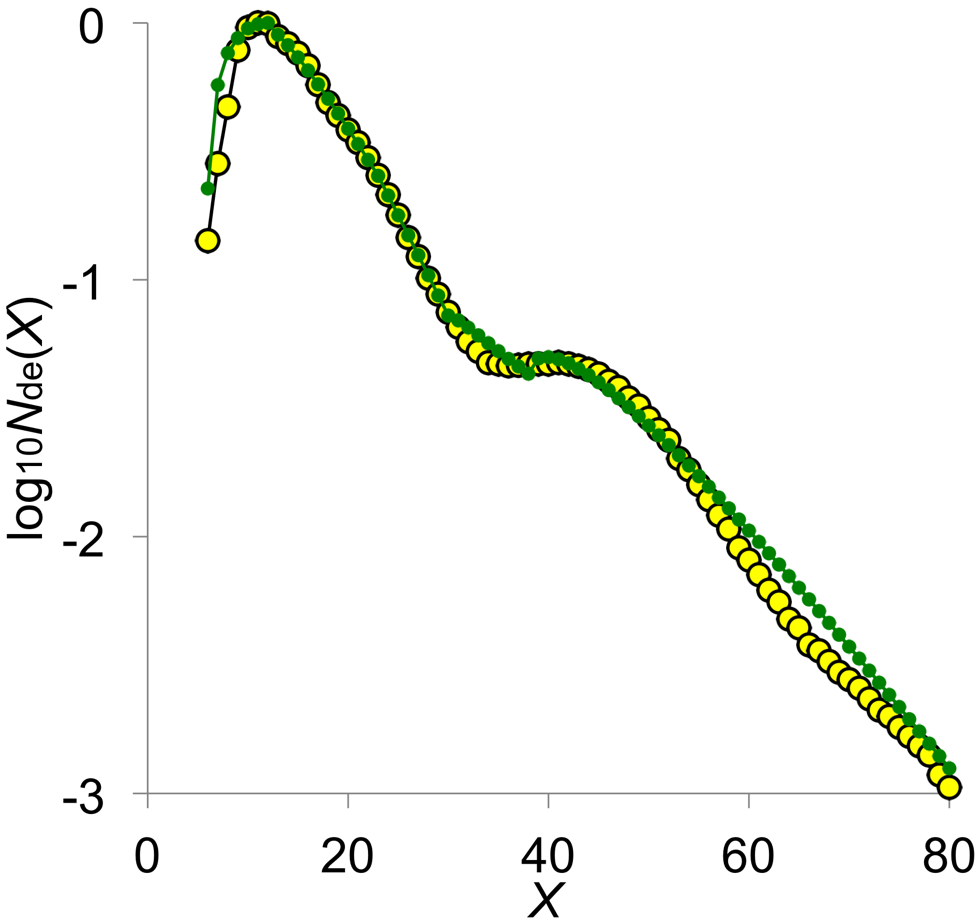

Supplement: Figure S9 — Optimized fitting to the single-lamella range (DP≤30) of the CLD in Figure S8. (TIF) [file pone.0065768.s009.tif]

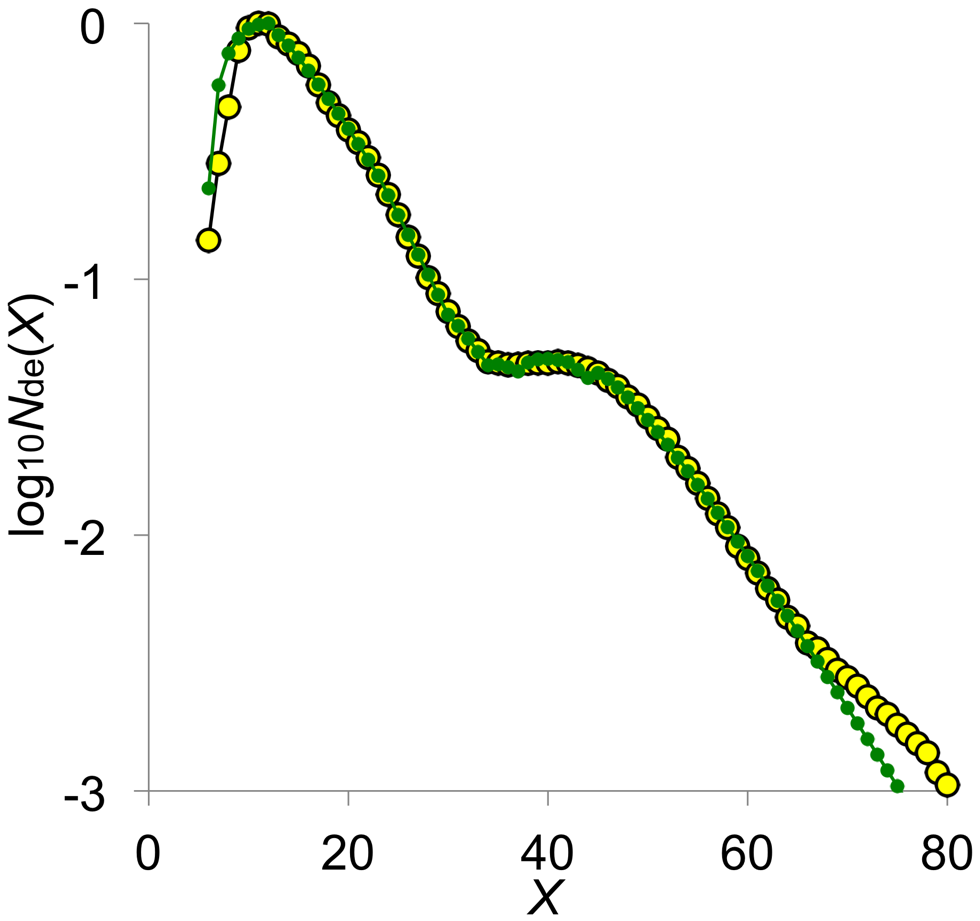

Supplement: Figure S10 — Optimized fitting to the trans-lamella range (DP>30) of the CLD in Figure S9. (TIF) [file pone.0065768.s010.tif]

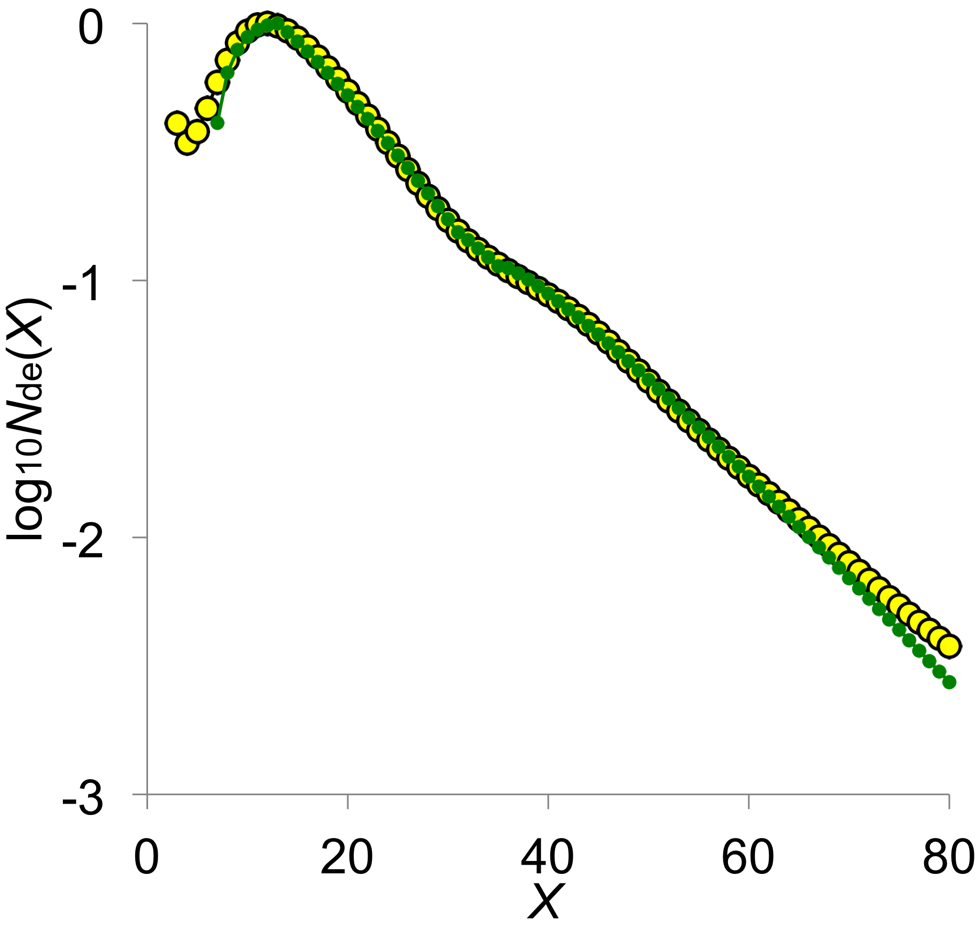

Supplement: Figure S11 — Optimized fitting (green circles) to a typical amylopectin CLD (yellow circles) obtained by SEC. (TIF) [file pone.0065768.s011.tif]
