# Supplementary material for: A Parameterized Model of Amylopectin Synthesis Provides Key Insights into the Synthesis of Granular Starch
Source: PLoS One. 2013 Jun 7;8(6):e65768. doi: 10.1371/journal.pone.0065768 (PMC3676345; doi:10.1371/journal.pone.0065768)
Supplement: Text S1 — Derivation of the time-evolution equation for the solution of CLD. (PDF) [file pone.0065768.s012.pdf]

Derivation of the time-evolution equation for the solution of CLD

The advance on our treatment is a Michaelis-Menten approach for enzyme kinetics compared to our previous model [1]. Chain growth by the action of SS in enzyme set α, SS(α), as given in the main body of text where α = i, ii, iii, iv. The rates of change in the number of individual chain lengths in the non-lamellar phase are given in (Eqn S1–S3). For convenience, $N_{\text{de}} = N_{\text{de.NL}}(X, t)$.

$$N_{\text{de}}(X) + g(t) + \text{SS}(\alpha) \underset{k_{2\text{SS}(\alpha)}}{\overset{k_{1\text{SS}(\alpha)}}{\rightleftarrows}} N_{\text{de.SS}(\alpha)\text{ complex}}(X) \xrightarrow{k_{3\text{SS}(\alpha)}} N_{\text{de}}(X+1) + \text{SS}(\alpha)$$

$$\left.\frac{\partial N_{\text{de}}}{\partial t}\right|_{\text{SS}(\alpha)} = -k_{1\text{SS}(\alpha)}[\text{SS}(\alpha)]g(t)N_{\text{de}}(X) + k_{2\text{SS}(\alpha)}N_{\text{de.SS}(\alpha)\text{ complex}}(X)$$

$$+k_{3\text{SS}(\alpha)}N_{\text{de.SS}(\alpha)\text{ complex}}(X-1)H(X-2) \qquad \text{Eqn S1}$$

$$\left.\frac{\partial N_{\text{de.SS}(\alpha)\text{ complex}}}{\partial t}\right|_{\text{SS}(\alpha)} = k_{1\text{SS}(\alpha)}[\text{SS}(\alpha)]g(t)N_{\text{de}}(X) - k_{2\text{SS}(\alpha)}N_{\text{de.SS}(\alpha)\text{ complex}}(X)$$

$$-k_{3\text{SS}(\alpha)}N_{\text{de.SS}(\alpha)\text{ complex}}(X) \qquad \text{Eqn S2}$$

$$\left.\frac{d[\text{SS}(\alpha)]}{dt}\right|_{\text{SS}(\alpha)} = \sum_{X=2}^{\infty}\{-k_{1\text{SS}(\alpha)}[\text{SS}(\alpha)]g(t)N_{\text{de}}(X) + k_{2\text{SS}(\alpha)}N_{\text{de.SS}(\alpha)\text{ complex}}(X)$$

$$+k_{3\text{SS}(\alpha)}N_{\text{de.SS}(\alpha)\text{ complex}}(X)\} \qquad \text{Eqn S3}$$

$H(y)$ is a step function: $H(y) = 0$ for $y < 0$, $= 1$ for $y \geq 0$, and appears in (Eqn S1) because X is synthesized from $X - 1$ by the action of SS (i.e. DP X gives $X + 1$ by propagation); the smallest physically possible X is 1, and the smallest possible product from SS is $X = 2$. The concentration of ADP-glucose at a given time is denoted $g(t)$.

The same development is applied to other SSs in other enzyme sets. α = i, ii, iii, iv for different enzyme sets.

Branching (SBE) as given in the main body of text. The notations given below are generalized for all SBEs. The enzymatic process is:

$$N_{\text{de}}(X)+\text{SBE}(\alpha)\underset{k_{2\text{SBE}(\alpha)}}{\overset{k_{1\text{SBE}(\alpha)}}{\rightleftarrows}}N_{\text{de.SBE}(\alpha)\text{ complex}}(X)\xrightarrow{k_{3\text{SBE}(\alpha)}}N_{\text{de}}(X_i)+N_{\text{de}}(X_j)+\text{SBE}(\alpha), X=X_i+X_j$$

$$\left.\frac{\partial N_{\text{de}}}{\partial t}\right|_{\text{SBE}(\alpha)}=-k_{1\text{SBE}(\alpha)}[\text{SBE}(\alpha)]N_{\text{de}}(X)+k_{2\text{SBE}(\alpha)}N_{\text{de.SBE}(\alpha)\text{ complex}}(X)$$

$$+k_{3\text{SBE}(\alpha)}\sum_{k=X}^{\infty}\frac{N_{\text{de.SBE}(\alpha)\text{ complex}}(k+X_0)}{k-X_{\min}+1}H(X-X_{\min})$$

$$+k_{3\text{SBE}(\alpha)}\sum_{k=X}^{\infty}\frac{N_{\text{de.SBE}(\alpha)\text{ complex}}(k+X_{\min})}{k-X_0+1}H(X-X_0) \qquad \text{Eqn S4}$$

$$\left.\frac{\partial N_{\text{de.SBE}(\alpha)\text{ complex}}}{\partial t}\right|_{\text{SBE}(\alpha)}=k_{1\text{SBE}(\alpha)}[\text{SBE}(\alpha)]N_{\text{de}}(X)-k_{2\text{SBE}(\alpha)}N_{\text{de.SBE}(\alpha)\text{ complex}}(X)$$

$$-k_{3\text{SBE}(\alpha)}N_{\text{de.SBE}(\alpha)\text{ complex}}(X)H\left(X-\left(X_{\min}+X_0\right)\right) \qquad \text{Eqn S5}$$

$$\left.\frac{d[\text{SBE}(\alpha)]}{dt}\right|_{\text{SBE}(\alpha)}=\sum_{X=2}^{\infty}\{-k_{1\text{SBE}(\alpha)}[\text{SBE}(\alpha)]N_{\text{de}}(X)+k_{2\text{SBE}(\alpha)}N_{\text{de.SBE}(\alpha)\text{ complex}}(X)$$

$$+k_{3\text{SBE}(\alpha)}N_{\text{de.SBE}(\alpha)\text{ complex}}(X)H\left(X-\left(X_0+X_{\min}\right)\right)\} \qquad \text{Eqn S6}$$

Debranching (DBE).

$$N_{\text{de}}(X)+\text{DBE}(\alpha)\underset{k_{2\text{DBE}(\alpha)}}{\overset{k_{1\text{DBE}(\alpha)}}{\rightleftarrows}}N_{\text{de.DBE}(\alpha)\text{ complex}}(X)\xrightarrow{k_{3\text{DBE}(\alpha)}}\text{DBE}(\alpha)$$

$$\left.\frac{\partial N_{\text{de}}}{\partial t}\right|_{\text{DBE}(\alpha)}=-k_{1\text{DBE}(\alpha)}[\text{DBE}(\alpha)]N_{\text{de}}(X)+k_{2\text{DBE}(\alpha)}N_{\text{de.DBE}(\alpha)\text{ complex}}(X) \qquad \text{Eqn S7}$$

$$\left.\frac{\partial N_{\text{de.DBE}(\alpha)\text{ complex}}}{\partial t}\right|_{\text{DBE}(\alpha)}=k_{1\text{DBE}(\alpha)}[\text{DBE}(\alpha)]N_{\text{de}}(X)-k_{2\text{DBE}(\alpha)}N_{\text{de.DBE}(\alpha)\text{ complex}}(X)$$

$$-k_{3\text{DBE}(\alpha)}N_{\text{de.DBE}(\alpha)\text{ complex}}(X) \qquad \text{Eqn S8}$$

$$\left.\frac{d[\text{DBE}(\alpha)]}{dt}\right|_{\text{DBE}(\alpha)}=\sum_{X=1}^{\infty}\{-k_{1\text{DBE}(\alpha)}[\text{DBE}(\alpha)]N_{\text{de}}(X)+k_{2\text{DBE}(\alpha)}N_{\text{de.DBE}(\alpha)\text{ complex}}(X)$$

$$+k_{3\text{DBE}(\alpha)}N_{\text{de.DBE}(\alpha)\text{ complex}}(X)\} \qquad \text{Eqn S9}$$

The time evolution of the non-lamellar CLD confined to the SL space is then given by:

$$\frac{\partial N_{\text{de}}}{\partial t}=\left.\frac{\partial N_{\text{de}}}{\partial t}\right|_{\text{SS(i)}}+\left.\frac{\partial N_{\text{de}}}{\partial t}\right|_{\text{SS(ii)}}+\left.\frac{\partial N_{\text{de}}}{\partial t}\right|_{\text{SBE(i)}}+\left.\frac{\partial N_{\text{de}}}{\partial t}\right|_{\text{SBE(ii)}}+\left.\frac{\partial N_{\text{de}}}{\partial t}\right|_{\text{DBE(i)}}+\left.\frac{\partial N_{\text{de}}}{\partial t}\right|_{\text{DBE(ii)}}$$

$$+\left.\frac{\partial N_{\text{de.SS(i) complex}}}{\partial t}\right|_{\text{SS(i)}}+\left.\frac{\partial N_{\text{de.SS(ii) complex}}}{\partial t}\right|_{\text{SS(ii)}}+\left.\frac{\partial N_{\text{de.SBE(i) complex}}}{\partial t}\right|_{\text{SBE(i)}}+\left.\frac{\partial N_{\text{de.SBE(ii) complex}}}{\partial t}\right|_{\text{SBE(ii)}}$$

$$+\left.\frac{\partial N_{\text{de.DBE(i) complex}}}{\partial t}\right|_{\text{DBE(i)}}+\left.\frac{\partial N_{\text{de.DBE(ii) complex}}}{\partial t}\right|_{\text{DBE(ii)}} \qquad \text{Eqn S10}$$

Two steady-state approximations can be made: the enzyme-substrate complex, $N_{\text{de.E complex}}$, reaches an equilibrium rapidly, also the amount of free enzymes, [E], is in an equilibrium with the complex. I.e.:

$$\left.\frac{\partial N_{\text{de.E complex}}}{\partial t}\right|_{\text{E}} = \left.\frac{\partial [\text{E}]}{\partial t}\right|_{\text{E}} = 0$$

Applying the steady-state approximation to (Eqn S2):

$$k_{1\text{SS}(\alpha)}[\text{SS}(\alpha)]g(t)N_{\text{de}}(X) = k_{2\text{SS}(\alpha)}N_{\text{de.SS}(\alpha)\text{ complex}}(X) + k_{3\text{SS}(\alpha)}N_{\text{de.SS}(\alpha)\text{ complex}}(X)$$

$$k_{1\text{SS}(\alpha)}[\text{SS}(\alpha)]g(t)N_{\text{de}}(X) = \left(k_{2\text{SS}(\alpha)} + k_{3\text{SS}(\alpha)}\right)N_{\text{de.SS}(\alpha)\text{ complex}}(X)$$

$$\frac{k_{1\text{SS}(\alpha)}}{k_{2\text{SS}(\alpha)} + k_{3\text{SS}(\alpha)}}[\text{SS}(\alpha)]g(t)N_{\text{de}}(X) = \left[N_{\text{de.SS}(\alpha)\text{ complex}}(X)\right]$$

The same treatment is applied to (Eqn S5 and S8). Making this steady-state approximation for the complex terms ($N_{\text{de.SS}(\alpha)\text{ complex}}$, etc., and in each enzyme) reduces the various equations in all quantities to ones in N_{de} alone:

$$\frac{\partial N_{\text{de}}(X,t)}{\partial t} = \left(\hat{a}_{\text{SS(i)}}g(t) + \hat{a}_{\text{SS(ii)}}g(t)\right)\left[N_{\text{de}}(X-1) - N_{\text{de}}(X)\right]$$

$$-a_{\text{SBE(i)}}N_{\text{de}}(X)H\left(X - \left(X_{0\text{(i)}} + X_{\min\text{(i)}}\right)\right) + a_{\text{SBE(i)}}\sum_{k=X}^{\infty}\frac{N_{\text{de}}\left(k + X_{0\text{(i)}}\right)}{k - X_{\min\text{(i)}} + 1}H\left(X - X_{\min\text{(i)}}\right)$$

$$+a_{\text{SBE(i)}}\sum_{k=X}^{\infty}\frac{N_{\text{de}}\left(k + X_{\min\text{(i)}}\right)}{k - X_{0\text{(i)}} + 1}H\left(X - X_{0\text{(i)}}\right)$$

$$-a_{\text{SBE(ii)}}N_{\text{de}}(X)H\left(X - \left(X_{0\text{(ii)}} + X_{\min\text{(ii)}}\right)\right) + a_{\text{SBE(ii)}}\sum_{k=X}^{\infty}\frac{N_{\text{de}}\left(k + X_{0\text{(ii)}}\right)}{k - X_{\min\text{(ii)}} + 1}H\left(X - X_{\min\text{(ii)}}\right)$$

$$+a_{\text{SBE(ii)}}\sum_{k=X}^{\infty}\frac{N_{\text{de}}\left(k + X_{\min\text{(ii)}}\right)}{k - X_{0\text{(ii)}} + 1}H\left(X - X_{0\text{(ii)}}\right)$$

$$-\left(a_{\mathrm{DBE(i)}}+a_{\mathrm{DBE(ii)}}\right)N_{\mathrm{de}}(X)-f_{\mathrm{cryst}}N_{\mathrm{de}}(X)+r(X) \qquad \text{Eqn S11}$$

where

$$\hat{a}_{\mathrm{SS(i)}}=\frac{k_{\mathrm{1SS(i)}}k_{\mathrm{3SS(i)}}}{k_{\mathrm{2SS(i)}}+k_{\mathrm{3SS(i)}}}[\mathrm{SS(i)}];a_{\mathrm{SS(i)}}(t)=\hat{a}_{\mathrm{SS(i)}}g(t) \qquad \text{Eqn S12}$$

$$a_{\mathrm{SBE(i)}}=\frac{k_{\mathrm{1SBE}(\alpha)}k_{\mathrm{3SBE}(\alpha)}H(X-(X_{\mathrm{0(i)}}+X_{\mathrm{min(i)}}))}{k_{\mathrm{2SBE}(\alpha)}+k_{\mathrm{3SBE}(\alpha)}H(X-(X_{\mathrm{0(i)}}+X_{\mathrm{min(i)}}))}[\mathrm{SBE(i)}] \qquad \text{Eqn S13}$$

$$a_{\mathrm{DBE(i)}}=\frac{k_{\mathrm{1DBE}(\alpha)}k_{\mathrm{3DBE(a)}}}{k_{\mathrm{2DBE}(\alpha)}+k_{\mathrm{3DBE(a)}}}[\mathrm{DBE(i)}] \qquad \text{Eqn S14}$$

and analogous for enzyme set (i) where, (i) is replaced with (ii)

References

1. Wu AC, Gilbert RG (2010) Molecular Weight Distributions of Starch Branches Reveal Genetic Constraints on Biosynthesis. Biomacromolecules 11: 3539-3547.
