# Supplementary material for: A Parameterized Model of Amylopectin Synthesis Provides Key Insights into the Synthesis of Granular Starch
Source: PLoS One. 2013 Jun 7;8(6):e65768. doi: 10.1371/journal.pone.0065768 (PMC3676345; doi:10.1371/journal.pone.0065768)
Supplement: Text S2 — Type-2 TL chains are independent from the SL chains. (PDF) [file pone.0065768.s013.pdf]

Type-2 TL chains are independent from the SL chains

Fitting (Figure 3A) shows that the features in the trans-lamella (TL) CLD are not reproduced from single-lamella (SL) kinetics alone. We test the possibility of invoking extra enzyme sets. The features in the TL CLD (Figure 3B) are less distinguishable; a simpler treatment is that one enzyme set dominates the TL range. This supposed enzyme set, then, incorporates the contributions of two sets: substrate-competing model. Therefore a total of three enzyme sets is modeled. This extension involves putting in the contributions from an extra of each of SS, SBE and DBE in Eqn 1 and derive to obtain Eqn 5 to solve for $N_{\mathrm{de}}(X)$. However, the calculated three-enzyme-sets CLD do not yield a quantitative fit to TL CLD of Nipponbare amylopectin CLD (Figure S1). This result implies that the type-2 TL chains have to form independently of the SL chains.
