# Supplementary material for: A Parameterized Model of Amylopectin Synthesis Provides Key Insights into the Synthesis of Granular Starch
Source: PLoS One. 2013 Jun 7;8(6):e65768. doi: 10.1371/journal.pone.0065768 (PMC3676345; doi:10.1371/journal.pone.0065768)
Supplement: Text S3 — Program “APCLDFIT” manual. (PDF) [file pone.0065768.s014.pdf]

PROGRAM “APCLDFIT”

Least-squares fit of experimental amylopectin chain-length distributions to parameterized model for biosynthesis

Authors: Alex Chi Wu, Robert G Gilbert

The University of Queensland, Centre for Nutrition and Food Sciences, Queensland Alliance for Agricultural and Food Innovation, Brisbane, QLD 4072, Australia

Referencing: this program should be referenced as:

“Fortran program APCLDFIT, Alex C Wu and Robert G Gilbert, The University of Queensland, 2013.”

Always check for updates which can be downloaded from https://sourceforge.net/projects/starchcldfit/?source=directory

Or alternatively search for “starchcldfit” at http://sourceforge.net/ and link on the search result “Starch CLD fit”.

Questions and comments can be posted and viewed in the Discussion (a sourceforge account is required to post in the Troubleshoot forum). By subscribing to Starch CLD fit, users will be automatically notified for updates and posts.

S1 S2 S3 S4 S5 S6

Overview

This FORTRAN program package finds the parameters involved in amylopectin chain-length distribution (CLD) biosynthesis by fitting experimental CLD data to a

model which incorporates the underlying starch biosynthesis. The CLD data are obtained by first debranching the starch with a debranching enzyme, then characterizing the resulting linear starch branches using any of FACE (fluorophore-assisted carbohydrate electrophoresis), HPAEC (high performance anion exchange chromatography) or SEC (size-exclusion chromatography, sometimes termed GPC, gel-permeation chromatography). The CLD is denoted by $N_{\mathrm{de}}(X)$: the number distribution of chains with a degree of polymerization (DP) of X glucose residues. Note that FACE and HPAEC give $N_{\mathrm{de}}(X)$ directly, whereas SEC gives $X^2N_{\mathrm{de}}(X)$ [1]. The amylopectin CLD biosynthesis theory used by this program is explained in the article "A Parameterized Model of Amylopectin Synthesis provides key insights into the Synthesis of Granular Starch", by Alex Chi Wu, Matthew K. Morell and Robert G. Gilbert, and in [2]. The following gives a summary of the model; the papers should be consulted before use of this program.

The *amylopectin CLD biosynthesis model* assumes that the synthesis of the amylopectin CLD involves only three classes of enzymes and their various isoforms: starch synthases (SSs), starch branching enzymes (SBEs) and debranching enzymes (DBE). One of each enzyme from different classes (e.g. SSIIA, SBEIA, isoamylase [a type of DBE]) comprise an *enzyme set*. Two enzyme sets govern the chains confined to a single lamella (SL); another two enzyme sets govern the chains that reside in the amorphous lamella, the type-2 trans-lamella (TL) chains. The overall CLD is formed from the actions of several enzyme sets acting simultaneously.

A sample of amylopectin CLD from FACE is given in Figure S7, with descriptions of the various features, which are taken from the article. These features come from

particular enzyme sets and restrictions on the biosynthetic enzymes (e.g. X_0 and X_{min} on SBEs), which have been described in our earlier model [2] and in the article.

Using this program, one can fit the CLD of amylopectin with this model. The fitting parameters comprise: (1) the relative branching activities, β, which is the activity of SBE divided by that of SS, for each enzyme set ($\beta_{(i)}$, $\beta_{(ii)}$, …), where (i), (ii) etc. denote each enzyme set; (2) the relative debranching activities acting on the SL CLD, $\gamma_{(i,ii)}$, which is the total activity of DBEs from enzyme set (i) and (ii) divided by that of SS from enzyme set (i) and (ii), $\gamma_{(iii,iv)}$ for that of the type-2 TL CLD (which is not an independent variable and is calculated in the code from other parameters); (3) the two minimum chain-length constraints on the action of SBEs, X_0 and X_{min} (also one of each per enzyme set: $X_{0(i)}$, $X_{min(i)}$, $X_{0(ii)}$, $X_{min(ii)}$ …); (4) the contribution to the overall CLD of the type-2 TL CLD relative to that of the SL CLD, $h_{(iii/i)}$.

Program structure

The program package contains a main program APCLDFIT.f and the data files as described in the subsection below.

Data files

It is recommended that users input their own data by editing the sample data files (except for DF_EXPNde_F_H.txt or DF_EXPNde_S.txt; see data file number 3 of the following section) with a text editor such as “TextEdit” or “WordPad”. **Do not use Microsoft Excel**, as this can change things such as line break characters to make the file unreadable by the code.

1. Data file **DF_tolerance.txt**

Contains the tolerance parameters for the non-linear least squares fitting (explained in input parameter numbers 21–29 in the following section).

2. Data file **DF_paras.txt**

Contains the input parameters (explained in input parameter numbers 1–20 in the following section).

3. Data file **DF_EXPNde_F_H.txt** or **DF_EXPNde_S.txt**

DF_EXPNde_F_H.txt contains a $N_{de}(X)$ from either FACE or HPAEC to be fitted by APCLDFIT; the equivalent for SEC data is stored in DF_EXPNde_S.txt. A sample of both is supplied in the program package. Only one of the files is needed for fitting at any one time; however, ensure that both files exist and only make changes to the appropriate file (e.g. if fitting $N_{de}(X)$ from FACE, prepare only DF_EXPNde_F_H.txt, while leaving DF_EXPNde_S.txt unchanged). File preparation for both is explained below and must be followed to ensure correct format.

FACE and HPAEC data give $N_{de}(X)$ directly as relative percentage for a series of integers X.

To prepare a new DF_EXPNde_F_H.txt for FACE or HPAEC data: (1) create an Excel spread sheet which contains the experimental CLD; (2) save with the name “DF_EXPNde_F_H.txt” as a Windows Formatted Text in Unix or Tab delimited Text in Windows.

SEC generates the SEC distribution, denoted $w(\log X)$. $N_{de}(X)$ is calculated from this with the relationship $N_{de}(X) = X^{-2}\ w(\log X)$. The $N_{de}(X)$ from SEC will be finely spaced from a continuous distribution (e.g. $X = 1.00, 1.01, 1.02 \ldots$); the program performs linear interpolation to find the approximate $N_{de}(X)$ at integer values of X ($X = 1, 2, 3, \ldots, 100$).

To prepare a new DF_EXPNde_S.txt for SEC data: (1) create an Excel spread sheet which contains the experimental CLD. Note that SEC gives a continuous DP. All data points within a particular range should be supplied in ascending DP. The range starts from the DP closest to 1 until the DP which is just above 100); (2) save with the name "DF_EXPNde_S.txt" as a Windows Formatted Text in Unix or Tab delimited Text in Windows.

4. Data file **DF_wSL.txt**

Specifies weightings for each data point in the SL range (i.e. SLrS – SLrE). The default is to treat all data points equally (i.e. weight = 1 for all DPs). If the user wishes to obtain better agreement for particular DP(s) between the calculated and experimental CLD, this can be achieved by increasing the weighting for the corresponding DP(s).

To prepare DF_wSL.txt: (1) create an Excel spread sheet which contains the weighting required; (2) save with the name "DF_wSL.txt" as a Windows Formatted Text.

5. Data file **DF_wTL.txt**

The same as DF_wSL.txt, for designating the weighting used in the TL range.

Input parameters

The following parameters are in **DF_paras.txt**

1. **CT**

Integer; the characterization technique used for obtaining the experimental amylopectin CLD to be fitted by the program. If CT = 1, FACE or HPAEC; if CT = 2, SEC.

2. **SLrS**

Integer; the starting X for the fitting range considered in the non-linear least squares fitting in the SL (single-lamella) range.

3. **SLrE**

Integer; the finishing X for the fitting range considered in the non-linear least squares fitting in the SL range.

4. **TLrE**

Integer; the finishing X for the fitting range considered in the non-linear least squares fitting in the TL (trans-lamella) range.

5. **beta(i)**

Floating point; the initial guess of $\beta_{(\mathrm{i})}$ for enzyme set (i).

6. **X0(i)**

Integer; the initial guess of $X_{0(\mathrm{i})}$ from enzyme set (i).

7. **Xmin(i)**

Integer; the initial guess of $X_{\min(i)}$ for enzyme set (i).

8. **By_pass(i)**

Integer; if By_pass(i) = 1, optimization of $X_{0(i)}$ and $X_{\min(i)}$ from enzyme set (i) will not be considered and the program uses the initial values supplied; if By_pass(i) = 0, optimization will be carried out based on the initial values supplied.

If CT = 2, input numbers 9–12 will not be used, but dummy input must be supplied (e.g. 0 for each).

9. **beta(ii)**

Floating point; the initial guess of $\beta_{(ii)}$ for enzyme set (ii).

10. **X0(ii)**

Integer; the initial guess of $X_{0(ii)}$ for enzyme set (ii).

11. **Xmin(ii)**

Integer; the initial guess of $X_{\min(ii)}$ from enzyme set (ii).

12. **By_pass(ii)**

Integer; if By_pass(ii) = 1, optimization of $X_{0(ii)}$, $X_{\min(ii)}$ for enzyme set (ii) will not be considered and the program uses the initial values supplied; if By_pass(ii) = 0, optimization will be carried out based on the initial values supplied.

13. **beta(iii)**

Floating point; the initial guess of $\beta_{(iii)}$ for enzyme set (iii).

14. **X0(iii)**

Integer; the initial guess of $X_{0(iii)}$ for enzyme set (iii).

15. **Xmin(iii)**

Integer; the initial guess of $X_{min(iii)}$ for enzyme set (iii).

16. **By_pass(iii)**

Integer; if By_pass(iii) = 1, optimization of $X_{0(iii)}$, $X_{min(iii)}$ for enzyme set (iii) will not be considered and the program uses the initial values supplied; if By_pass(iii) = 0, optimization will be carried out based on the initial values supplied.

If CT = 2, input numbers 17–20 will not be used, but dummy input must be supplied (e.g. 0 for each).

17. **beta(iv)**

Floating point; the initial guess of $\beta_{(iv)}$ for enzyme set (iv).

18. **X0(iv)**

Integer; the initial guess of $X_{0(iv)}$ for enzyme set (iv).

19. **Xmin(iv)**

Integer; the initial guess of $X_{min(iv)}$ for enzyme set (iv).

20. **By_pass(iv)**

Integer; if By_pass(iv) = 1, optimization of $X_{0(iv)}$, $X_{min(iv)}$ for enzyme set (iv) will not be considered and the program uses the initial values supplied; if By_pass(iv) = 0, optimization will be carried out based on the initial values supplied.

The following parameters are in **DF_tolerance.txt**

21. **Xmax**

Integer; numerical parameter to ensure convergence. The total number of DPs X in the calculation (the order of **Ω** in Eqn 2 of the paper) is truncated at Xmax, which must be a sufficiently large value. The test of this is when the calculated $N_{de}(X)$ converges to the desired precision (supplementary information of [2]). Xmax = 110 is recommended for fitting amylopectin CLDs.

If CT = 2, input numbers 23, 25, 27 and 29 will not be used, but dummy input must be supplied (e.g. put a 0 in place).

22. **DX(i)**

Floating point up to 4 decimal places; initial increment of beta(i) employed in the non-linear least squares fitting.

23. **DX(ii)**

Same as DX(i), but for beta(ii).

24. **DX(iii)**

Same as DX(i), but for beta(iii).

25. **DX(iv)**

Same as DX(i), but for beta(iv).

26. **ACCX(i)**

Floating point; the desired accuracy of beta(i) employed in the non-linear least squares fitting.

27. **ACCX(ii)**

Same as ACCX(i), but for beta(ii).

28. **ACCX(iii)**

Same as ACCX(i), but for beta(iii).

29. **ACCX(iv)**

Same as ACCX(i), but for beta(iv).

Program output

The program generates three output files: (1) The file "Input_data.txt" gives all inputs in the data files: DF_EXPNde_F_H.txt or DF_EXPNde_S.txt, DF_paras.txt, DF_tolerance.txt, DF_wSL.txt and DF_wTL.txt; (2) the file "Final_result.txt" gives the overall experimental CLD and the overall calculated CLD; and (3) the file "Fitting_refinement_data.txt" gives the fine details of fitting.

Input_data.txt. This file lists the input parameters and the experimental CLD read in by the program. Users are encouraged to check this file to ensure the desired parameters and experiment CLD are entered correctly. The program will overwrite an existing file of the same name or create one if it does not exist.

Final_result.txt. For most users, this is all that is needed, which gives the overall experimental CLD (either from FACE, HPAEC or SEC) and the overall calculated CLD fitted to the experiment. The best way of plotting the CLDs is with a logarithmic Y axis, so that one has a plot of $\log_{10}N_{de}(X)$ against X [1,2]. This is exemplified in Figure S7.

Final_result.txt also gives the fitted parameters for the overall calculated CLD. Most of the fitted parameters are described in the previous section. Extra fitted parameters are:

1. The relative debranching activities for the SL and the type-2 TL CLDs:
    a. If CT = 1, gamma(i,ii) and gamma(iii,iv), which are the fitted value of $\gamma_{(i,ii)}$ and $\gamma_{(iii,iv)}$, respectively or;
    b. If CT = 2, gamma(i) and gamma(iii), which are the fitted value of $\gamma_{(i)}$ and $\gamma_{(iii)}$, respectively;
2. TLstart = the starting DP of the type-2 TL chains (the program determines TLstart = SLrE + 1);
3. h(iii/i) = the ratio of the maximum of the type–2 TL CLD to that of the SL CLD, $h_{(iii/i)}$.

The default output for Final_result.txt is displayed directly in Terminal if running in Unix or Command Prompt in Windows. In Unix the result can be transferred to a new Microsoft Excel spreadsheet and named “Final_result” by copy and paste. This is ready for plotting. For Windows, the following instruction is recommended, which is also applicable in Unix.

Generate Final_result.txt directly via Terminal or Command Prompt as follows. In Unix, if the compiled file is called “fit”, one would run the program with the command line

```
./fit > Final_result.txt
```

In Windows, the command line is:

```
fit > Final_result.txt
```

Final_result.txt can then be opened directly from Microsoft Excel for plotting.

Fitting_refinement_data.txt. This provides the fine details of fitting, which can be plotted on a logarithmic Y axis to determine if refinement is needed. The program will overwrite an existing file of the same name or create one if it does not exist. Fitting_refinement_data.txt can be opened directly from Microsoft Excel for plotting.

If CT = 1, output file 3 will contain 4 blocks of data, as appropriate for FACE or HPAEC experimental CLD. The first block of data contains the fitting results to the SL chains of the experimental CLD from all possible combinations of $X_{0(i)}$ and $X_{min(i)}$, while holding $X_{0(ii)}$ and $X_{min(ii)}$ constant. The first column gives the DP, followed by the overall experimental CLD, then the possible fits to the SL CLD with different combinations of $X_{0(i)}$ and $X_{min(i)}$ determined by the program. The best possible fit is placed first. The optimal fit is determined by the smallest least-squares residual of the fit. The second block of data is analogous to the first, but for optimizing $X_{0(ii)}$ and $X_{min(ii)}$ for enzyme set (ii), while employing the optimal $X_{0(i)}$ and $X_{min(i)}$ from enzyme set (i) optimization. The third block of data contains the fitting results to the type-2 TL chains of the experimental CLD from all possible

combinations of $X_{0(iii)}$ and $X_{min(iii)}$ for enzyme set (iii), while holding $X_{0(iv)}$ and $X_{min(iv)}$ constant. This time the first column gives the DP, followed by the overall experimental type-2 TL CLD, then the possible fits to the SL CLD determined by the program. The fourth block is analogous to the third except is for optimizing $X_{0(iii)}$ and $X_{min(iii)}$ for enzyme set (iv), while holding $X_{0(iii)}$ and $X_{min(iii)}$ constant.

The best fit from data blocks 2 and 4 can be plotted (e.g. Figure 2a,b in the article) to determine if fittings require refinements; they show more detail. The fitting parameters are reported for all fittings. If either input numbers 8, 12, 16 or 20 = 1 (i.e. by-pass enzyme set (i), (ii), (iii) or (iv) optimization), a fit based on the initial input parameters for the corresponding enzyme set will be generated.

If CT = 2, the output of the program contains 2 blocks of data, as appropriate for SEC experimental data. The structure of output is similar to CT = 1 except there is no enzyme set (ii) and (iv). In this case, the best fit from data blocks 1 and 2 can be plotted (e.g. Figure 2a,b in the article) to determine if fittings require refinements.

Examples of fitting amylopectin CLD with the program package

This section gives an example each for fitting CLD data obtained by FACE or HPAEC and SEC with the program package. Users are recommended to examine the example thoroughly before treating their own CLDs.

Fitting CLDs obtained by either FACE or HPAEC

This example uses the CLD in Figure S7. Ensure all data files are prepared as appropriate for fitting CLD obtained by FACE or HPAEC. The fitting procedures can be divided into three main sections as follows.

1. Preliminary fitting of the CLD. Make a guess of the SL range, which is between SLrS and SLrE, in the overall CLD. It is recommended to set SLrS = 10. Users can employ the values of beta(i) – (iv) supplied in the program package. Make a guess of the parameters: X0(i), Xmin(i), X0(ii) and Xmin(ii) for the SL range by examining the CLD. X0(i) is usually the starting DP of the CLD (= 6); Xmin(i) is roughly the DP at which the global maximum starts (≈ 8). The same principle is applied to X0(ii) and Xmin(ii), where their values may be 10 and 15, respectively. X0(iii), Xmin(iii), X0(iv) and Xmin(iv) for the TL range can be set to the following. Set both X0(iii) and X0(iv) to 1 and set Xmin(iii) and Xmin(iv) equal to Xmin(i) and Xmin(ii), respectively. Make a guess of TLrE. Set parameter numbers 8, 12, 16 and 20 = 1 to by pass optimizations for enzyme set (i) – (iv) in this preliminary fitting. Figure S8 shows a typical preliminary fit.
2. Optimize fitting to the SL range. It is worthwhile refining the preliminary fitting (Figure S8) manually for the SL range, if the fitting is significantly different to the experimental CLD, prior to optimization. The preliminary fitting can be manually refined in the following way. In the example given here, a smaller Xmin(i) is needed since the calculated global maximum is slightly higher than the experimental CLD. In the contrary, a larger Xmin(i) will be needed if calculated CLD gives the global maximum at a lower DP.

The same principle is applied to X0(ii) and Xmin(ii). Make appropriate changes to X0(i), Xmin(i), X0(ii) and Xmin(ii) to generate a refined fit. If user is satisfied with the changes, set input numbers 8 and 12 = 0 to optimize X0(i), Xmin(i), X0(ii) and Xmin(ii). User input is needed to refine SLrE if required. The goal is to fit as many data points as possible on the near-linear slope after Feature C (Figure S7). One way to refine SLrE, given that the experimental CLD is sufficiently accurate, is by comparing the experimental type-2 TL CLD to that of the calculated CLD (the last block of data in Fitting_refinement_data.txt). For example, if the experiment is significantly lower than the calculated in the first few DPs, this usually indicates that a larger SLrE is needed. A fit with optimal SL range is shown in Figure S9.

3. Optimize fitting to the TL range. It may be difficult to manually refine the input parameters for the TL range as described above for the SL range. The features in the TL range are less prominent. It is recommended to go straight into optimization by setting input number 16 and 20 = 0 to determine the best X0(iii), Xmin(iii), X0(iv) and Xmin(iv). During this, input numbers 8 and 12 are set to 1, since enzyme set (i) and (ii) are already optimized. User input is needed to refine TLrE if required: fit as many data points as possible on the near-linear slope after Feature E (Figure S7). Feature F is ascribed by additional enzyme sets not included in the present model and should not be fitted. The optimal fitting for the TL range is shown in Figure S10. This shows a typical optimized fit to the overall amylopectin CLD obtained by FACE or HPAEC. Note that the imperfect behavior at DP $\gtrsim 65$ is ascribed to the presence of additional enzyme sets at these higher DPs.

Fitting CLDs obtained by SEC

Ensure all data files are prepared as appropriate for fitting CLD obtained by SEC. Fitting CLDs obtained by SEC is analogous to that described for FACE or HPAEC except that beta, X0 and Xmin for enzyme set (ii) and (iv) (i.e. input numbers 9–12 and 17–20) are not required. A typical optimized fit to the overall amylopectin CLD obtained by SEC is shown in Figure S11. The behavior at the very short DP (< 6) may or may not be present, but may be due to band broadening of SEC and should be ignored in the non-linear least squares fitting, which is simply achieved with the default setting SLrS = 10 (above). The calculated CLD often has a sharper Feature B compared to experimental CLD obtained by SEC (Figure S11). This is because only enzyme set (i) is present as opposed to enzyme set (i) and (ii) in the SL range. The imperfect behavior at DP $\gtrsim$ 60 is ascribed to the presence of additional enzyme sets at these higher DPs.

References

1. Castro JV, Dumas C, Chiou H, Fitzgerald MA, Gilbert RG (2005) Mechanistic information from analysis of molecular weight distributions of starch. Biomacromolecules 6: 2248-2259.
2. Wu AC, Gilbert RG (2010) Molecular Weight Distributions of Starch Branches Reveal Genetic Constraints on Biosynthesis. Biomacromolecules 11: 3539-3547.
