# Supplementary material for: A Parameterized Model of Amylopectin Synthesis Provides Key Insights into the Synthesis of Granular Starch
Source: PLoS One. 2013 Jun 7;8(6):e65768. doi: 10.1371/journal.pone.0065768 (PMC3676345; doi:10.1371/journal.pone.0065768)
Supplement: Text S4 — APCLDFIT code. (PDF) [file pone.0065768.s015.pdf]

APCLDFIT program

```
      Implicit real*8 (a-h, o-z)
      DOUBLE PRECISION A(1000,1000),b(1000)

      DOUBLE PRECISION Bmask(1000),EXPX(1000),q
      DOUBLE PRECISION AA(1000,1000),AAA(1000)
      DOUBLE PRECISION b1(1000),b2(1000),b3(1000)

      INTEGER DP(1000),EXPmax,EXPTLmax
      DOUBLE PRECISION EXPNde(1000)

      DOUBLE PRECISION EXPN(1000),EXPN1(1000),EXPN1d(1000)

      DOUBLE PRECISION wSL(1000),wTL(1000)

      DOUBLE PRECISION beta1,beta2
      DOUBLE PRECISION betai,betaii,beteiii,betaiv
      DOUBLE PRECISION gammai_ii,gammaiii_iv
      DOUBLE PRECISION gamma

      INTEGER Xmin1,Xmin2,X01,X02
      INTEGER Xmini,Xminii,Xminiii,Xminiv,X0i,X0ii,X0iii,X0iv

      INTEGER XminiA(1000),X0iA(1000),XminiiA(1000),X0iiA(1000)
      INTEGER XminiiiA(1000),X0iiiA(1000),XminivA(1000),X0ivA(1000)

      INTEGER Xmax,Norm
      INTEGER icount,CT,op0,opi,opii,opiii,opiv

      INTEGER opri,oprii,opriii,opriv
      INTEGER mingap,enzi_ii

      INTEGER SLrS,SLrE,TLrS,TLrE,TLstart

      INTEGER N,COUNT
      DOUBLE PRECISION X(10),DX(10),ACCX(10),DXp(10),ACCXp(10)

      INTEGER XmaxSEC,xpo
      DOUBLE PRECISION xa(1000),ya(1000),ypo,mypo

      DOUBLE PRECISION nmaxva

      DOUBLE PRECISION resSL, resTL

      INTEGER mx

      CHARACTER label(16)
      character DPNAM(3),EXPNAM(40)

      common /tran1/ NP,N1,MP
      common /tran2/ XMAX,Norm
      common /tran6/ SLrS,SLrE,TLrS,TLrE,TLstart
      common /tran5/ EXPN,EXPN1,EXPN1d
      common /tran9/ wSL,wTL
      common /tran8/ Xmini,Xminii,Xminiii,Xminiv,X0i,X0ii,X0iii,X0iv
      common /tran4/ gamma
      common /tran7/ b
      common /tran10/ resSL,resTL
      common /tran11/ EXPNAM
      common /tran12/ iTL,CT,mingap

      LOGICAL CONV
      EXTERNAL FUN1
      EXTERNAL FUN2

!     Available 307


      NP = 1000
      MP = 1
      N1 = 1

      OPEN(unit=13,file='DF_tolerance.txt',status='old')
      OPEN(unit=14,file='DF_paras.txt',status='old')
      OPEN(15,file='DF_EXPNde_F_H.txt',status='old')
```

```
      OPEN(11,file='DF_EXPNde_S.txt',status='old')
      OPEN(17,file='DF_wSL.txt',status='old')
      OPEN(18,file='DF_wTL.txt',status='old')

      OPEN(unit=12,file='Input_data.txt',status='replace')

      OPEN(unit=16,file='Fitting_refinement_data.txt',
     1 status='replace')

      WRITE(12,*) 'Input parameters:'
!       READING PARAMETERS IN FILE 'tolerance.txt'-------------
      WRITE(12,*) ''
      WRITE(12,*) 'tolerance.txt'
      READ(13,157) XMAX
      WRITE(12,178) Xmax

      Norm=XMAX

      READ(13,156) DXp(1)
      WRITE(12,170) DXp(1)
      READ(13,156) DXp(2)
      WRITE(12,171) DXp(2)
      READ(13,156) DXp(3)
      WRITE(12,172) DXp(3)
      READ(13,156) DXp(4)
      WRITE(12,173) DXp(4)

      READ(13,156) ACCXp(1)
      WRITE(12,174) ACCXp(1)
      READ(13,156) ACCXp(2)
      WRITE(12,175) ACCXp(2)
      READ(13,156) ACCXp(3)
      WRITE(12,176) ACCXp(3)
      READ(13,156) ACCXp(4)
      WRITE(12,177) ACCXp(4)

 157  FORMAT('                ',I8)
 156  FORMAT('                ',F8.4)

 178  FORMAT('Xmax =   ',I4)
 170  FORMAT('DX(i) =  ',F8.4)
 171  FORMAT('DX(ii) = ',F8.4)
 172  FORMAT('DX(iii) =       ',F8.4)
 173  FORMAT('DX(iv) = ',F8.4)
 174  FORMAT('ACCX(i) =       ',F8.4)
 175  FORMAT('ACCX(ii) =      ',F8.4)
 176  FORMAT('ACCX(iii) =     ',F8.4)
 177  FORMAT('ACCX(iv) =      ',F8.4)

!       READING PARAMETERS IN FILE 'paras.txt'-----------------
      WRITE(12,*) ''
      WRITE(12,*) 'paras.txt'
      READ(14,157) CT
      WRITE(12,179) CT

      READ(14,157) SLrS
      WRITE(12,180) SLrS
      READ(14,157) SLrE
      WRITE(12,181) SLrE
      TLstart=SLrE+1
      READ(14,157) TLrE
      WRITE(12,182) TLrE

      READ(14,156) betai
      WRITE(12,183) betai
      READ(14,157) X0i
      WRITE(12,184) X0i
      READ(14,157) Xmini
      WRITE(12,185) Xmini
      READ(14,157) opi
      WRITE(12,186) opi

      READ(14,156) betaii
      WRITE(12,187) betaii
      READ(14,157) X0ii
      WRITE(12,188) X0ii
      READ(14,157) Xminii
      WRITE(12,189) Xminii
      READ(14,157) opii
```

```
      WRITE(12,190) opii

      READ(14,156) betaiii
      WRITE(12,191) betaiii
      READ(14,157) X0iii
      WRITE(12,192) X0iii
      READ(14,157) Xminiii
      WRITE(12,193) Xminiii
      READ(14,157) opiii
      WRITE(12,194) opiii

      READ(14,156) betaiv
      WRITE(12,195) betaiv
      READ(14,157) X0iv
      WRITE(12,196) X0iv
      READ(14,157) Xminiv
      WRITE(12,197) Xminiv
      READ(14,157) opiv
      WRITE(12,198) opiv

 179  FORMAT('CT =     ',I1)
 180  FORMAT('SLrS =   ',I2)
 181  FORMAT('SLrE =   ',I2)
 182  FORMAT('TLrE =   ',I2)
 183  FORMAT('beta(i) =       ',F8.4)
 184  FORMAT('X0(i) =  ',I2)
 185  FORMAT('Xmin(i) =       ',I2)
 186  FORMAT('By_pass(i) =    ',I1)

 187  FORMAT('beta(ii) =      ',F8.4)
 188  FORMAT('X0(ii) = ',I2)
 189  FORMAT('Xmin(ii) =      ',I2)
 190  FORMAT('By_pass(ii) =   ',I1)

 191  FORMAT('beta(iii) =     ',F8.4)
 192  FORMAT('X0(iii) =       ',I2)
 193  FORMAT('Xmin(iii) =     ',I2)
 194  FORMAT('By_pass(iii) =  ',I1)

 195  FORMAT('beta(iv) =      ',F8.4)
 196  FORMAT('X0(iv) = ',I2)
 197  FORMAT('Xmin(iv) =      ',I2)
 198  FORMAT('By_pass(iv) =   ',I1)

!       READING PARAMETERS IN FILE 'EXPNde.txt'----------------
!   IF CT = 1 (FACE/HPAEC)
      IF (CT .EQ. 1) THEN
        READ(15,20) DPNAM,EXPNAM
  20    FORMAT(3A1,40A1)
        DO 21 i=1,1000
        READ(15,*,END=19) DP(i), EXPNde(i)
        EXPmax=DP(i)
  21    CONTINUE

  19    CALL maxv(EXPNde,EXPmax,nmaxva)
!         WRITE(6,*) nmaxva

        DO 14 i=1,EXPmax
        EXPN(i)=EXPNde(i)/nmaxva
  14    CONTINUE

      ELSE
! converting SEC Nde(X) with continuous X to discrete values of X by linear
interpolation
!   IF CT NE 1 (SEC)
        XmaxSEC = 0
        READ(11,20) DPNAM,EXPNAM
        DO 26 i=1,1000
        READ(11,*,END=27) xa(i), ya(i)
!         WRITE(6,999) xa(i),ya(i)
        XmaxSEC = XmaxSEC + 1
  26    CONTINUE

! 999  FORMAT(1p,E16.8,'      ',1p,E16.8)

  27    EXPmax=100
!         WRITE(6,*) XmaxSEC

        DO 146 i=1,XmaxSEC
```

```
        IF(i .GE. xa(1)) GO TO 147
 146    CONTINUE

 147    DO 148 k=1,i-2
        DP(k)=k
 148    CONTINUE

        DO 154 xpo=i-1,EXPmax
        DO 152 j=1,XmaxSEC

        DP(xpo)=xpo

        IF (DBLE(xpo) .EQ. xa(j)) THEN
          EXPNde(xpo)=ya(j)
        ELSE IF (DBLE(xpo) .GT. xa(j) .AND. DBLE(xpo) .LT. xa(j+1)) THEN
          mypo= (ya(j+1)-ya(j))/(xa(j+1)-xa(j))
          EXPNde(xpo)=mypo*(DBLE(xpo)-xa(j))+ya(j)
        END IF

 152    CONTINUE
 154    CONTINUE

        CALL maxv(EXPNde,EXPmax,nmaxva)

        DO 153 i=1,EXPmax
        EXPN(i)=EXPNde(i)/nmaxva
 153    CONTINUE

      END IF

      DO 29 i=1,EXPmax
      AA(10+i,1)=i
      AA(10+i,2)=EXPN(i)
  29  CONTINUE

      WRITE(12,*) ''
      WRITE(12,*) 'EXPNde.txt'
      IF(CT .EQ. 1) THEN
      WRITE(12,*) 'DP  EXPNde (FACE/HPAEC)'
      ELSE
      WRITE(12,*) 'DP  EXPNde (SEC)'
      END IF
      DO 155 i=1,EXPmax
      WRITE(12,10) DP(i),EXPNde(i)
 155  CONTINUE

      WRITE(12,*) ''
      IF(CT .EQ. 1) THEN
      WRITE(12,*) 'DP  EXPNde (FACE/HPAEC) normalized'
      ELSE
      WRITE(12,*) 'DP  EXPNde (SEC) normalized'
      END IF
      DO 22 i=1,EXPmax
      WRITE(12,10) DP(i),EXPN(i)
  22  CONTINUE

  10  FORMAT(I3,'      ',1p,E16.8)

!       READING PARAMETERS IN FILE 'wSL.txt and wTL.txt'-------
      READ(17,145) label
      DO 23 i=1,Xmax
      READ(17,*) DP(i), wSL(i)
  23  CONTINUE
 145  FORMAT(40A1)

      READ(18,145) label
      DO 30 i=1,Xmax
      READ(18,*) DP(i), wTL(i)
  30  CONTINUE

      WRITE(12,*) ''
      WRITE(12,*) 'wSL & wTL.txt'
      WRITE(12,*) 'DP  SL Weighting   TL Weighting'
      DO 24 i=1,Xmax
      WRITE(12,199) DP(i), wSL(i), wTL(i)
  24  CONTINUE

 199  FORMAT(I3,'      ',F8.4,'       ',F8.4)
```

```
!       CALCULATING SL CLD=====================================================
      enzi_ii=1
      IF (CT .EQ. 1) THEN
      N=2
      ELSE
      N=1
      END IF

!   By-pass enzyme set (i) optimization--------------------
      IF (opi .EQ. 1) GO TO 140

      DO 201 opri=1,20
      mingap=1
!   FUNMIN SPECS------------------------------------------
      icount=0
 112  IF (icount .EQ. 9) GO TO 102
      icount=icount+1

      IF (icount .EQ. 2) Xmini=Xmini-1
      IF (icount .EQ. 3) Xmini=Xmini+2
      IF (icount .EQ. 4) Xmini=Xmini-2
      IF (icount .EQ. 4) X0i=X0i-1
      IF (icount .EQ. 5) Xmini=Xmini+1
      IF (icount .EQ. 6) Xmini=Xmini+1
      IF (icount .EQ. 7) Xmini=Xmini-2
      IF (icount .EQ. 7) X0i=X0i+2
      IF (icount .EQ. 8) Xmini=Xmini+1
      IF (icount .EQ. 9) Xmini=Xmini+1

      X(1)=betai
      X(2)=betaii
      DX(1)=DXp(1)
      DX(2)=DXp(2)

      ACCX(1)=ACCXp(1)
      ACCX(2)=ACCXp(1)

      COUNT=1000

c      WRITE(6,200) icount,X0i,Xmini
c 200  FORMAT('icount =       'I2,/,'X0i =   'I2,/,'Xmini = 'I2)

!   CALL FUNMIN-------------------------------------------
      CALL FUNMIN(FUN1,X,DX,N,ACCX,CONV,COUNT)

!   STORE SOLUTIONS---------------------------------------
      AA(1,2+icount)=X(1)
      AA(2,2+icount)=X(2)
      AA(3,2+icount)=gamma

      AA(4,2+icount)=Xmini
      AA(5,2+icount)=X0i
      AA(6,2+icount)=Xminii
      AA(7,2+icount)=X0ii

      AA(8,2+icount)=SLrS
      AA(9,2+icount)=SLrE

      IF(CT .EQ. 1) THEN
        IF(AA(4,2+icount) .LT. 6 .OR. AA(5,2+icount) .LT. 6) THEN
        AA(10,2+icount)=1000*resSL
        ELSE
        AA(10,2+icount)=resSL
        END IF
      ELSE
      AA(10,2+icount)=resSL
      END IF

      DO 64 i=1,EXPmax
      AA(10+i,2+icount)=b(i)
  64  CONTINUE

!   Enzyme set (i) optimization----------------------------
      GO TO 112

 102  CALL rearrange_AA(AA,icount,EXPmax,enzi_ii)

      gammai_ii=AA(3,3)
      Xmini=AA(4,3)
```

```
      X0i=AA(5,3)

      XminiA(opri)=AA(4,3)
      X0iA(opri)=AA(5,3)

c      WRITE(6,202) opri,X0iA(opri),XminiA(opri)
c 202  FORMAT(/,'opri =       'I2,/,'X0i =   'I2,/,'Xmini = 'I2,/)

      IF(opri .EQ. 1) GO TO 201

!   DETERMINE & STORE OPTIMISED SOLUTION
      IF (XminiA(opri) .EQ. XminiA(opri-1) .AND.
     1 X0iA(opri) .EQ. X0iA(opri-1)) THEN
      DO 204 i=1,Xmax*2
      DO 205 j=4,Xmax*2
      AA(i,j)=0
 205  CONTINUE
 204  CONTINUE
      GO TO 140
      END IF

 201  CONTINUE

 140  mingap=2
      icount=1

      X(1)=betai
      X(2)=betaii
      DX(1)=DXp(1)
      DX(2)=DXp(2)

      ACCX(1)=ACCXp(1)
      ACCX(2)=ACCXp(1)

      COUNT=1000

!      WRITE(6,206) icount,X0i,Xmini
! 206  FORMAT('Calculaing optimized solution',/,
!     1 'icount =      'I2,/,'X0i =   'I2,/,'Xmini = 'I2)

      CALL FUNMIN(FUN1,X,DX,N,ACCX,CONV,COUNT)

      AA(1,2+icount)=X(1)
      AA(2,2+icount)=X(2)
      AA(3,2+icount)=gamma

      AA(4,2+icount)=Xmini
      AA(5,2+icount)=X0i
      AA(6,2+icount)=Xminii
      AA(7,2+icount)=X0ii

      AA(8,2+icount)=SLrS
      AA(9,2+icount)=SLrE

      AA(10,2+icount)=resSL

      DO 203 i=1,EXPmax
      AA(10+i,2+icount)=b(i)
 203  CONTINUE

!      WRITE(6,*) 'Optimized solution stored'

!   Print optimized Xmin(i) & X0(i) guess------------------
      WRITE(16,*) 'AC Wu, RG Gilbert, Biomacromolecules 11 3539 2010'
      WRITE(16,*) 'AC Wu, MK Morell, RG Gilbert submitted 2013'
      WRITE(16,*) ''
      WRITE(16,31) EXPNAM
  31  FORMAT('Fitting_single-lamella_CLD_of: ',40A1)
      WRITE(16,*) 'Trying_different_combinations_of_Xmin(i)_&_X0(i)'
      CALL WRITE_SLsolution_mx(AA,icount,EXPmax)


      enzi_ii=2
      IF(CT .NE. 1) GO TO 251
!   By-pass Enzyme set (ii) (SEC)--------------------------
      IF (opii .EQ. 1) THEN
!   By-pass Enzyme set (ii) optimization-------------------
      icount=1
      GO TO 250
```

```
      END IF

      DO 252 oprii=1,20
      mingap=1
!   FUNMIN SPECS------------------------------------------
      icount=0
 254  IF (icount .EQ. 9) GO TO 255
      icount=icount+1

      IF (icount .EQ. 2) Xminii=Xminii-1
      IF (icount .EQ. 3) Xminii=Xminii+2
      IF (icount .EQ. 4) Xminii=Xminii-2
      IF (icount .EQ. 4) X0ii=X0ii-1
      IF (icount .EQ. 5) Xminii=Xminii+1
      IF (icount .EQ. 6) Xminii=Xminii+1
      IF (icount .EQ. 7) Xminii=Xminii-2
      IF (icount .EQ. 7) X0ii=X0ii+2
      IF (icount .EQ. 8) Xminii=Xminii+1
      IF (icount .EQ. 9) Xminii=Xminii+1

      X(1)=betai
      X(2)=betaii
      DX(1)=DXp(1)
      DX(2)=DXp(2)

      ACCX(1)=ACCXp(1)
      ACCX(2)=ACCXp(1)

      COUNT=1000

!      WRITE(6,263) icount,X0ii,Xminii
! 263  FORMAT('icount =       'I2,/,'X0ii =  'I2,/,'Xminii =        'I2)

!   CALL FUNMIN-------------------------------------------
      CALL FUNMIN(FUN1,X,DX,N,ACCX,CONV,COUNT)

!   STORE SOLUTIONS---------------------------------------
      AA(1,2+icount)=X(1)
      AA(2,2+icount)=X(2)
      AA(3,2+icount)=gamma

      AA(4,2+icount)=Xmini
      AA(5,2+icount)=X0i
      AA(6,2+icount)=Xminii
      AA(7,2+icount)=X0ii

      AA(8,2+icount)=SLrS
      AA(9,2+icount)=SLrE

      IF(CT .EQ. 1) THEN
        IF(AA(6,2+icount) .LT. 6 .OR. AA(7,2+icount) .LT. 6) THEN
        AA(10,2+icount)=1000*resSL
        ELSE
        AA(10,2+icount)=resSL
        END IF
      ELSE
      AA(10,2+icount)=resSL
      END IF

!      WRITE(6,265) resSL
! 265  FORMAT('resSL = ',E16.8)

!      DO 267 i=1,EXPmax
!      WRITE(6,266) i,EXPN(i),b(i)
! 266  FORMAT(I4,'     ',1p,E16.8,'   ',1p,E16.8)
! 267  CONTINUE

      DO 253 i=1,EXPmax
      AA(10+i,2+icount)=b(i)
 253  CONTINUE

!   Enzyme set (ii) optimization---------------------------
      GO TO 254

 255  CALL rearrange_AA(AA,icount,EXPmax,enzi_ii)

      gammai_ii=AA(3,3)
      Xminii=AA(6,3)
      X0ii=AA(7,3)
```

```
      XminiiA(oprii)=AA(6,3)
      X0iiA(oprii)=AA(7,3)

!       WRITE(6,256) oprii,X0iiA(oprii),XminiiA(oprii)
! 256  FORMAT(/,'oprii =      'I2,/,'X0ii =  'I2,/,'Xminii =        'I2,/)

      IF(oprii .EQ. 1) GO TO 252

!   DETERMINE & STORE OPTIMISED SOLUTION
      IF (XminiiA(oprii) .EQ. XminiiA(oprii-1) .AND.
     1 X0iiA(oprii) .EQ. X0iiA(oprii-1)) THEN
      DO 258 i=1,Xmax*2
      DO 257 j=4,Xmax*2
      AA(i,j)=0
 257  CONTINUE
 258  CONTINUE
      GO TO 264
      END IF

 252  CONTINUE

 264  mingap=2
      icount=1

      X(1)=betai
      X(2)=betaii
      DX(1)=DXp(1)
      DX(2)=DXp(2)

      ACCX(1)=ACCXp(1)
      ACCX(2)=ACCXp(1)

      COUNT=1000

!       WRITE(6,259) icount,X0ii,Xminii
! 259  FORMAT('Calculaing optimized solution',/,
!      1 'icount =      'I2,/,'X0ii =  'I2,/,'Xminii =        'I2)

      CALL FUNMIN(FUN1,X,DX,N,ACCX,CONV,COUNT)

      AA(1,2+icount)=X(1)
      AA(2,2+icount)=X(2)
      AA(3,2+icount)=gamma

      AA(4,2+icount)=Xmini
      AA(5,2+icount)=X0i
      AA(6,2+icount)=Xminii
      AA(7,2+icount)=X0ii

      AA(8,2+icount)=SLrS
      AA(9,2+icount)=SLrE

      AA(10,2+icount)=resSL

      DO 260 i=1,EXPmax
      AA(10+i,2+icount)=b(i)
 260  CONTINUE

!       WRITE(6,*) 'Optimized solution stored'

!   Print optimized Xmin(i) & X0(i) guess------------------
 250  WRITE(16,*) ''
      WRITE(16,262) EXPNAM
 262  FORMAT('Fitting_single-lamella_CLD_of: ',40A1)
      WRITE(16,*) 'Trying_different_combinations_of_Xmin(ii)_&_X0(ii)'
      CALL WRITE_SLsolution_mx(AA,icount,EXPmax)

      betai=X(1)
      betaii=X(2)
      gammai_ii=gamma

 251  DO 268 i=1,EXPmax
      b1(i)=b(i)
 268  CONTINUE

      DO 304 i=1,Xmax*2
      DO 305 j=1,Xmax*2
      AA(i,j)=0
```

```
 305  CONTINUE
 304  CONTINUE




!       CALCULATING TL CLD============================================================
      enzi_ii=1
!   Prepare EXPN - SL CLD---------------------------------
      DO 269 i=1,EXPmax
      EXPN1(i)=EXPN(i)-b1(i)
 269  CONTINUE

!      WRITE(6,*) ''
!      WRITE(6,*) 'X   EXPN1'
!      DO 270 i=1,EXPmax
!      WRITE(6,10) DP(i),EXPN1(i)
! 270  CONTINUE

      EXPTLmax=EXPmax-TLstart+1
      iTL=EXPTLmax

      DO 271 i=1,EXPTLmax
      EXPN1d(i)=EXPN1(TLstart+i-1)
 271  CONTINUE

      CALL maxv(EXPN1d,EXPTLmax,nmaxva)
      cc=nmaxva

!      WRITE(6,*) ''
!      WRITE(6,*) 'X   EXPN1d'
      DO 272 i=1,EXPTLmax
      EXPN1d(i)=EXPN1d(i)/nmaxva
!      WRITE(6,10) DP(i),EXPN1d(i)
 272  CONTINUE

      DO 273 i=1,EXPTLmax
      AA(10+i,1)=i
      AA(10+i,2)=EXPN1d(i)
 273  CONTINUE

      TLrS=1
      DO 274 i=1,15
      IF(EXPN1d(i) .LT. 0.) TLrS=TLrS+1
 274  CONTINUE

      IF (CT .EQ. 1) THEN
      N=2
      ELSE
      N=1
      END IF

!   By-pass enzyme set (iii) optimization--------------------
      IF (opiii .EQ. 1) GO TO 275

      DO 276 opriii=1,20
      mingap=1
!   FUNMIN SPECS------------------------------------------
      icount=0
 277  IF (icount .EQ. 9) GO TO 278
      icount=icount+1

      IF (icount .EQ. 2) Xminiii=Xminiii-1
      IF (icount .EQ. 3) Xminiii=Xminiii+2
      IF (icount .EQ. 4) Xminiii=Xminiii-2
      IF (icount .EQ. 4) X0iii=X0iii-1
      IF (icount .EQ. 5) Xminiii=Xminiii+1
      IF (icount .EQ. 6) Xminiii=Xminiii+1
      IF (icount .EQ. 7) Xminiii=Xminiii-2
      IF (icount .EQ. 7) X0iii=X0iii+2
      IF (icount .EQ. 8) Xminiii=Xminiii+1
      IF (icount .EQ. 9) Xminiii=Xminiii+1

      IF (Xminiii .LT. 1 .OR. X0iii .LT. 1) THEN
      resTL = 1000
      GO TO 286
      END IF

      X(1)=betaiii
```

```
      X(2)=betaiv
      DX(1)=DXp(3)
      DX(2)=DXp(4)

      ACCX(1)=ACCXp(3)
      ACCX(2)=ACCXp(4)

      COUNT=1000

!   CALL FUNMIN-------------------------------------------
      CALL FUNMIN(FUN2,X,DX,N,ACCX,CONV,COUNT)

!   STORE SOLUTIONS---------------------------------------
      AA(1,2+icount)=X(1)
      AA(2,2+icount)=X(2)
      AA(3,2+icount)=gamma

      AA(4,2+icount)=Xminiii
      AA(5,2+icount)=X0iii
      AA(6,2+icount)=Xminiv
      AA(7,2+icount)=X0iv

      AA(8,2+icount)=TLrS
      AA(9,2+icount)=TLrE

      DO 279 i=1,EXPTLmax
      AA(10+i,2+icount)=b(i)
 279  CONTINUE

 286  AA(10,2+icount)=resTL
      WRITE(6,294) icount,X0iii,Xminiii,resTL
 294  FORMAT('icount = 'I2,'  X0iii = ',I2,' Xminiii = ',I2,
     1 '       resTL = ',1p,E18.6)

c      DO 308 i=1,EXPTLmax
c      WRITE(6,307) i,EXPN1d(i),b(i)
c 308  CONTINUE
c 307  FORMAT(I4,'     ',1p,E16.8,'   ',1p,E16.8)

!   Enzyme set (iii) optimization----------------------------
      GO TO 277

 278  CALL rearrange_AA(AA,icount,EXPmax,enzi_ii)

      gammaiii_iv=AA(3,3)
      Xminiii=AA(4,3)
      X0iii=AA(5,3)

      XminiiiA(opriii)=AA(4,3)
      X0iiiA(opriii)=AA(5,3)

      WRITE(6,*) 'opriii      X0iii   Xminiii'
      WRITE(6,280) opriii,X0iiiA(opriii),XminiiiA(opriii)
      WRITE(6,*) ''
 280  FORMAT(I2,'      ',I2,'  ',I2)

      IF(opriii .EQ. 1) GO TO 276

!   DETERMINE & STORE OPTIMISED SOLUTION
      IF (XminiiiA(opriii) .EQ. XminiiiA(opriii-1) .AND.
     1 X0iiiA(opriii) .EQ. X0iiiA(opriii-1)) THEN
      GO TO 275
      END IF

 276  CONTINUE

 275  mingap=2
      icount=1

      X(1)=betaiii
      X(2)=betaiv
      DX(1)=DXp(3)
      DX(2)=DXp(4)

      ACCX(1)=ACCXp(3)
      ACCX(2)=ACCXp(4)

      COUNT=1000
```

```
      WRITE(6,*) 'Calculating final solution'
      WRITE(6,283) icount,X0iii,Xminiii
 283  FORMAT('icount = 'I2,'  X0iii = ',I2,' Xminiii = ',I2)

      CALL FUNMIN(FUN2,X,DX,N,ACCX,CONV,COUNT)

      AA(1,2+icount)=X(1)
      AA(2,2+icount)=X(2)
      AA(3,2+icount)=gamma

      AA(4,2+icount)=Xminiii
      AA(5,2+icount)=X0iii
      AA(6,2+icount)=Xminiv
      AA(7,2+icount)=X0iv

      AA(8,2+icount)=TLrS
      AA(9,2+icount)=TLrE

      AA(10,2+icount)=resTL

      DO 284 i=1,EXPTLmax
      AA(10+i,2+icount)=b(i)
 284  CONTINUE

!      WRITE(6,*) 'Optimized solution stored'

!   Print optimized Xmin(iii) & X0(iii) guess------------------
      WRITE(16,*) ''
      WRITE(16,285) EXPNAM
 285  FORMAT('Fitting_type-2_trans-lamella_CLD_of:   ',40A1)
      WRITE(16,*) 'Trying_different_combinations_of_Xmin(iii)_&_X0(iii)'
      CALL WRITE_TLsolution_mx(AA,icount,EXPmax)
!-==-=-=-=-=-=-=-=-=-=-=-=-=-=-=-=-=-=-=-=-=-=-=-=-=-=-=-=-=-=-=-=-=-=-=-=-
=+++++++++++++

      DO 281 i=1,Xmax*2
      DO 282 j=4,Xmax*2
      AA(i,j)=0
 282  CONTINUE
 281  CONTINUE

      enzi_ii=2
      IF(CT .NE. 1) GO TO 287
!   By-pass Enzyme set (iv) (SEC)--------------------------
      IF (opiv .EQ. 1) THEN
!   By-pass Enzyme set (iv) optimization-------------------
      icount=1
      GO TO 288
      END IF

      DO 289 opriv=1,20
      mingap=1
!   FUNMIN SPECS-------------------------------------------
      icount=0
 291  IF (icount .EQ. 9) GO TO 290
      icount=icount+1

      IF (icount .EQ. 2) Xminiv=Xminiv-1
      IF (icount .EQ. 3) Xminiv=Xminiv+2
      IF (icount .EQ. 4) Xminiv=Xminiv-2
      IF (icount .EQ. 4) X0iv=X0iv-1
      IF (icount .EQ. 5) Xminiv=Xminiv+1
      IF (icount .EQ. 6) Xminiv=Xminiv+1
      IF (icount .EQ. 7) Xminiv=Xminiv-2
      IF (icount .EQ. 7) X0iv=X0iv+2
      IF (icount .EQ. 8) Xminiv=Xminiv+1
      IF (icount .EQ. 9) Xminiv=Xminiv+1

      IF (Xminiv .LT. 1 .OR. X0iv .LT. 1) THEN
      resTL = 1000
      GO TO 296
      END IF

      X(1)=betaiii
      X(2)=betaiv
      DX(1)=DXp(3)
      DX(2)=DXp(4)

      ACCX(1)=ACCXp(3)
```

```
      ACCX(2)=ACCXp(4)

      COUNT=1000

!   CALL FUNMIN-------------------------------------------
      CALL FUNMIN(FUN2,X,DX,N,ACCX,CONV,COUNT)

!   STORE SOLUTIONS---------------------------------------
      AA(1,2+icount)=X(1)
      AA(2,2+icount)=X(2)
      AA(3,2+icount)=gamma

      AA(4,2+icount)=Xminiii
      AA(5,2+icount)=X0iii
      AA(6,2+icount)=Xminiv
      AA(7,2+icount)=X0iv

      AA(8,2+icount)=TLrS
      AA(9,2+icount)=TLrE

      DO 292 i=1,EXPmax
      AA(10+i,2+icount)=b(i)
 292  CONTINUE

 296  AA(10,2+icount)=resTL
      WRITE(6,301) icount,X0iv,Xminiv,resTL
 301  FORMAT('icount = 'I2,'  X0iv = ',I2,'  Xminiv = ',I2,
     1 '       resTL = ',1p,E18.6)

!   Enzyme set (iv) optimization----------------------------
      GO TO 291

 290  CALL rearrange_AA(AA,icount,EXPmax,enzi_ii)

      gammaiii_iv=AA(3,3)
      Xminiv=AA(6,3)
      X0iv=AA(7,3)

      XminivA(opriv)=AA(6,3)
      X0ivA(opriv)=AA(7,3)

      WRITE(6,*) 'opriv       X0iv    Xminiv'
      WRITE(6,302) opriv,X0ivA(opriv),XminivA(opriv)
      WRITE(6,*) ''
 302  FORMAT(I2,'      ',I2,'  ','     ',I2)

      IF(opriv .EQ. 1) GO TO 289

!   DETERMINE & STORE OPTIMISED SOLUTION
      IF (XminivA(opriv) .EQ. XminivA(opriv-1) .AND.
     1 X0ivA(opriv) .EQ. X0ivA(opriv-1)) THEN
      DO 297 i=1,Xmax*2
      DO 298 j=4,Xmax*2
      AA(i,j)=0
 298  CONTINUE
 297  CONTINUE
      GO TO 293
      END IF

 289  CONTINUE

 293  mingap=2
      icount=1

      X(1)=betaiii
      X(2)=betaiv
      DX(1)=DXp(3)
      DX(2)=DXp(4)

      ACCX(1)=ACCXp(3)
      ACCX(2)=ACCXp(4)

      COUNT=1000

      WRITE(6,*) 'Calculating final solution'
      WRITE(6,303) icount,X0iv,Xminiv
 303  FORMAT('icount = 'I2,'  X0iv = ',I2,'  Xminiv = ',I2)

      CALL FUNMIN(FUN2,X,DX,N,ACCX,CONV,COUNT)
```

```
      AA(1,2+icount)=X(1)
      AA(2,2+icount)=X(2)
      AA(3,2+icount)=gamma

      AA(4,2+icount)=Xminiii
      AA(5,2+icount)=X0iii
      AA(6,2+icount)=Xminiv
      AA(7,2+icount)=X0iv

      AA(8,2+icount)=TLrS
      AA(9,2+icount)=TLrE

      AA(10,2+icount)=resTL

      DO 299 i=1,EXPTLmax
      AA(10+i,2+icount)=b(i)
 299  CONTINUE

!      WRITE(6,*) 'Optimized solution stored'

!   Print optimized Xmin(i) & X0(i) guess------------------
 288  WRITE(16,*) ''
      WRITE(16,295) EXPNAM
 295  FORMAT('Fitting_trans-lamella_CLD_of:  ',40A1)
      WRITE(16,*) 'Trying_different_combinations_of_Xmin(iv)_&_X0(iv)'
      CALL WRITE_SLsolution_mx(AA,icount,EXPmax)

      betaiii=X(1)
      betaiv=X(2)
      gammaiii_iv=gamma

 287  DO 300 i=1,EXPmax
      b2(TLstart+i-1)=b(i)
 300  CONTINUE

      DO 306 i=1,EXPmax
      AA(10+i,1)=i
 306  CONTINUE


      !   Print final solution-----------------------------------
      WRITE(6,*) 'AC Wu, RG Gilbert, Biomacromolecules 11 3539 2010'
      WRITE(6,*) 'AC Wu, MK Morell, RG Gilbert submitted 2013'
      WRITE(6,*) ''
      IF(CT .EQ. 1) THEN
      WRITE(6,141) EXPNAM
 141  FORMAT('Fitting_to_the_overall_experimental_CLD_(FACE/HPAEC)_of:
     1         ',40A1)
      WRITE(6,13)
     1 betai,X0i,Xmini,
     1 betaii,X0ii,Xminii,
     1 gammai_ii,
     1 betaiii,X0iii,Xminiii,
     1 betaiv,X0iv,Xminiv,
     1 gammaiii_iv,
     1 TLstart,cc

  13  FORMAT(
     1 'beta(i)                ',1p,E16.8,/,'X0i(i)          ',I2,/,
     1 'Xmin(i)                ',I2,/,
     1 'beta(ii)               ',1p,E16.8,/,'X0(ii)          ',I2,/,
     1 'Xmin(ii)               ',I2,/,
     1 'gamma(i,ii)            ',1p,E16.8,/,
     1 'beta(iii)              ',1p,E16.8,/,'X0i(iii)        ',I2,/,
     1 'Xmin(iii)              ',I2,/,
     1 'beta(iv)               ',1p,E16.8,/,'X0(iv)          ',I2,/,
     1 'Xmin(iv)               ',I2,/,
     1 'gamma(iii,iv)          ',1p,E16.8,/,
     1 'TLstart                ',I2,/,'h(iii/i)              ',1p,E16.8)

      ELSE
      WRITE(6,144) EXPNAM
 144  FORMAT('Fitting_to_the_overall_experimental_CLD_(SEC)_of:
     1         ',40A1)
      WRITE(6,15)
     1 betai,X0i,Xmini,
     1 gammai_ii,
     1 betaiii,X0iii,Xminiii,
```

```
     1 gammaiii_iv,
     1 TLstart,cc

  15  FORMAT(
     1 'beta(i)               ',1p,E16.8,/,'X0i(i)          ',I2,/,
     1 'Xmin(i)               ',I2,/,
     1 'gamma(i)              ',1p,E16.8,/,
     1 'beta(iii)             ',1p,E16.8,/,'X0i(iii)        ',I2,/,
     1 'Xmin(iii)             ',I2,/,
     1 'gamma(iii)            ',1p,E16.8,/,
     1 'TLstart               ',I2,/,'h(iii/i)              ',1p,E16.8)
      END IF

      WRITE(6,*) 'DP   Overall_experimental_CLD
     1  Overall_calculated_CLD'
      DO 136 i=1,Xmax-10
      b3(i)=b1(i)+cc*b2(i)
      IF(INT(AA(10+i,1)) .EQ. 0) THEN
      WRITE(6,*) ''
      GO TO 136
      END IF
      WRITE(6,12) INT(AA(10+i,1)),EXPN(i),b3(i)
 136  CONTINUE
  12  FORMAT(I4,'      ',1p,E16.8,'   ',1p,E16.8)


      CLOSE(13)
      CLOSE(14)
      CLOSE(15)
      CLOSE(11)
      CLOSE(17)
      CLOSE(18)

      CLOSE(12)

      CLOSE(16)

      STOP
      END




!     -1
      Subroutine rearrange_AA(AA,icount,EXPmax,enzi_ii)
      Implicit real*8 (a-h, o-z)
      DOUBLE PRECISION A(1000,1000),b(1000)

      DOUBLE PRECISION Bmask(1000),EXPX(1000),q
      DOUBLE PRECISION AA(1000,1000),AAA(1000)
      DOUBLE PRECISION b1(1000),b2(1000),b3(1000)

      INTEGER DP(1000),EXPmax,EXPTLmax
      DOUBLE PRECISION EXPNde(1000)

      DOUBLE PRECISION EXPN(1000),EXPN1(1000),EXPN1d(1000)

      DOUBLE PRECISION wSL(1000),wTL(1000)

      DOUBLE PRECISION beta1,beta2
      DOUBLE PRECISION betai,betaii,beteiii,betaiv
      DOUBLE PRECISION gammai_ii,gammaiii_iv
      DOUBLE PRECISION gamma

      INTEGER Xmin1,Xmin2,X01,X02
      INTEGER Xmini,Xminii,Xminiii,Xminiv,X0i,X0ii,X0iii,X0iv

      INTEGER XminiA(1000),X0iA(1000)

      INTEGER Xmax,Norm
      INTEGER icount,CT,op0,opi,opii,opiii,opiv

      INTEGER opri,oprii,opriii,opriv
      INTEGER mingap,enzi_ii

      INTEGER SLrS,SLrE,TLrS,TLrE,TLstart

      INTEGER N,COUNT
      DOUBLE PRECISION X(10),DX(10),ACCX(10),DXp(10),ACCXp(10)
```

```
      INTEGER XmaxSEC,xpo
      DOUBLE PRECISION xa(1000),ya(1000),ypo,mypo

      DOUBLE PRECISION nmaxva

      DOUBLE PRECISION resSL, resTL

      CHARACTER label(16)
      character DPNAM(3),EXPNAM(40)

      common /tran1/ NP,N1,MP
      common /tran2/ XMAX,Norm
      common /tran6/ SLrS,SLrE,TLrS,TLrE,TLstart
      common /tran5/ EXPN,EXPN1,EXPN1d
      common /tran9/ wSL,wTL
      common /tran8/ Xmini,Xminii,Xminiii,Xminiv,X0i,X0ii,X0iii,X0iv
      common /tran4/ gamma
      common /tran7/ b
      common /tran10/ resSL,resTL
      common /tran11/ EXPNAM
      common /tran12/ iTL,CT,mingap

      DO 621 i=3,icount+3-2
      DO 620 j=i,icount+3-2
      IF(AA(10,i) .LT. AA(10,j+1)) GO TO 620
      DO 622 k=1,10+EXPmax
      AAA(k)=AA(k,i)
      AA(k,i)=AA(k,j+1)
      AA(k,j+1)=AAA(k)
 622  CONTINUE
 620  CONTINUE
 621  CONTINUE

      IF(enzi_ii .EQ. 1) THEN
        IF(AA(4,3).LT.AA(5,3) .AND. (AA(4,3)+AA(5,3)).EQ.
     1   (AA(4,4)+AA(5,4)) .AND. AA(10,3).EQ.AA(10,4)) THEN
        DO 623 k=1,10+EXPmax
        AAA(k)=AA(k,3)
        AA(k,3)=AA(k,4)
        AA(k,4)=AAA(k)
 623    CONTINUE
        END IF
      ELSE
        IF(AA(6,3).LT.AA(7,3) .AND. (AA(6,3)+AA(7,3)).EQ.
     1   (AA(6,4)+AA(7,4)) .AND. AA(10,3).EQ.AA(10,4)) THEN
        DO 624 k=1,10+EXPmax
        AAA(k)=AA(k,3)
        AA(k,3)=AA(k,4)
        AA(k,4)=AAA(k)
 624    CONTINUE
        END IF

      END IF

      RETURN
      END


!     0
      Subroutine WRITE_SLsolution_mx(AA,icount,EXPmax)
      Implicit real*8 (a-h, o-z)
      DOUBLE PRECISION A(1000,1000),b(1000)

      DOUBLE PRECISION Bmask(1000),EXPX(1000),q
      DOUBLE PRECISION AA(1000,1000),AAA(1000)
      DOUBLE PRECISION b1(1000),b2(1000),b3(1000)

      INTEGER DP(1000),EXPmax,EXPTLmax
      DOUBLE PRECISION EXPNde(1000)

      DOUBLE PRECISION EXPN(1000),EXPN1(1000),EXPN1d(1000)

      DOUBLE PRECISION wSL(1000),wTL(1000)

      DOUBLE PRECISION beta1,beta2
      DOUBLE PRECISION betai,betaii,beteiii,betaiv
      DOUBLE PRECISION gammai_ii,gammaiii_iv
      DOUBLE PRECISION gamma
```

```fortran
      INTEGER Xmin1,Xmin2,X01,X02
      INTEGER Xmini,Xminii,Xminiii,Xminiv,X0i,X0ii,X0iii,X0iv

      INTEGER XminiA(1000),X0iA(1000)

      INTEGER Xmax,Norm
      INTEGER icount,CT,op0,opi,opii,opiii,opiv

      INTEGER opri,oprii,opriii,opriv
      INTEGER mingap

      INTEGER SLrS,SLrE,TLrS,TLrE,TLstart

      INTEGER N,COUNT
      DOUBLE PRECISION X(10),DX(10),ACCX(10),DXp(10),ACCXp(10)

      INTEGER XmaxSEC,xpo
      DOUBLE PRECISION xa(1000),ya(1000),ypo,mypo

      DOUBLE PRECISION nmaxva

      DOUBLE PRECISION resSL, resTL

      CHARACTER label(16)
      character DPNAM(3),EXPNAM(40)

      common /tran1/ NP,N1,MP
      common /tran2/ XMAX,Norm
      common /tran6/ SLrS,SLrE,TLrS,TLrE,TLstart
      common /tran5/ EXPN,EXPN1,EXPN1d
      common /tran9/ wSL,wTL
      common /tran8/ Xmini,Xminii,Xminiii,Xminiv,X0i,X0ii,X0iii,X0iv
      common /tran4/ gamma
      common /tran7/ b
      common /tran10/ resSL,resTL
      common /tran11/ EXPNAM
      common /tran12/ iTL,CT,mingap

c       OPEN(unit=16,file='Data_for_fitting_refinement.txt',
c      1 status='unknown')

      WRITE(16,510) (AA(1,j), j=3,icount+2)
 510  FORMAT('beta(i)         ',30(1p,E16.8,'        '))
      WRITE(16,513) (INT(AA(5,j)), j=3,icount+2)
 513  FORMAT('X0(i)           ',30(I2,'      '))
      WRITE(16,507) (INT(AA(4,j)), j=3,icount+2)
 507  FORMAT('Xmin(i)         ',30(I2,'      '))

      IF (CT .EQ. 1) THEN
      WRITE(16,511) (AA(2,j), j=3,icount+2)
 511  FORMAT('beta(ii)        ',30(1p,E16.8,'        '))
      WRITE(16,515) (INT(AA(7,j)), j=3,icount+2)
 515  FORMAT('X0(ii)          ',30(I2,'      '))
      WRITE(16,514) (INT(AA(6,j)), j=3,icount+2)
 514  FORMAT('Xmin(ii)        ',30(I2,'      '))
      END IF

      IF (CT .EQ. 1) THEN
      WRITE(16,512) (AA(3,j), j=3,icount+2)
      ELSE
      WRITE(16,518) (AA(3,j), j=3,icount+2)
      END IF
 512  FORMAT('gamma(i,ii)             ',30(1p,E16.8,'        '))
 518  FORMAT('gamma(i)        ',30(1p,E16.8,'        '))

      WRITE(16,516) (INT(AA(8,j)), j=3,icount+2)
 516  FORMAT('SLrS            ',30(I2,'      '))
      WRITE(16,517) (INT(AA(9,j)), j=3,icount+2)
 517  FORMAT('SLrE            ',30(I2,'      '))
      WRITE(16,508) (AA(10,j), j=3,icount+2)
 508  FORMAT('Residual        ',30(1p,E16.8,'        '))

      WRITE(16,551) (i, i=1,icount)
 551  FORMAT('DP      EXP     ',30('Fit',I1,'        '))

      DO 537 i=1,Xmax-10
        IF(INT(AA(10+i,1)) .EQ. 0) THEN
        WRITE(16,*) ''
```

```
        GO TO 537
        END IF
      WRITE(16,503) INT(AA(10+i,1)),(AA(10+i,j), j=2,icount+2)
 503  FORMAT(I4,'      ',30(1p,E16.8,'        '))
 537  CONTINUE


c      CLOSE(16)

      RETURN
      END

!     0.1
      Subroutine WRITE_TLsolution_mx(AA,icount,EXPmax)
      Implicit real*8 (a-h, o-z)
      DOUBLE PRECISION A(1000,1000),b(1000)

      DOUBLE PRECISION Bmask(1000),EXPX(1000),q
      DOUBLE PRECISION AA(1000,1000),AAA(1000)
      DOUBLE PRECISION b1(1000),b2(1000),b3(1000)

      INTEGER DP(1000),EXPmax,EXPTLmax
      DOUBLE PRECISION EXPNde(1000)

      DOUBLE PRECISION EXPN(1000),EXPN1(1000),EXPN1d(1000)

      DOUBLE PRECISION wSL(1000),wTL(1000)

      DOUBLE PRECISION beta1,beta2
      DOUBLE PRECISION betai,betaii,beteiii,betaiv
      DOUBLE PRECISION gammai_ii,gammaiii_iv
      DOUBLE PRECISION gamma

      INTEGER Xmin1,Xmin2,X01,X02
      INTEGER Xmini,Xminii,Xminiii,Xminiv,X0i,X0ii,X0iii,X0iv

      INTEGER XminiA(1000),X0iA(1000)

      INTEGER Xmax,Norm
      INTEGER icount,CT,op0,opi,opii,opiii,opiv

      INTEGER opri,oprii,opriii,opriv
      INTEGER mingap

      INTEGER SLrS,SLrE,TLrS,TLrE,TLstart

      INTEGER N,COUNT
      DOUBLE PRECISION X(10),DX(10),ACCX(10),DXp(10),ACCXp(10)

      INTEGER XmaxSEC,xpo
      DOUBLE PRECISION xa(1000),ya(1000),ypo,mypo

      DOUBLE PRECISION nmaxva

      DOUBLE PRECISION resSL, resTL

      CHARACTER label(16)
      character DPNAM(3),EXPNAM(40)

      common /tran1/ NP,N1,MP
      common /tran2/ XMAX,Norm
      common /tran6/ SLrS,SLrE,TLrS,TLrE,TLstart
      common /tran5/ EXPN,EXPN1,EXPN1d
      common /tran9/ wSL,wTL
      common /tran8/ Xmini,Xminii,Xminiii,Xminiv,X0i,X0ii,X0iii,X0iv
      common /tran4/ gamma
      common /tran7/ b
      common /tran10/ resSL,resTL
      common /tran11/ EXPNAM
      common /tran12/ iTL,CT,mingap

      WRITE(16,510) (AA(1,j), j=3,icount+2)
 510  FORMAT('beta(iii)                ',30(1p,E16.8,'        '))
      WRITE(16,513) (INT(AA(5,j)), j=3,icount+2)
 513  FORMAT('X0(iii)          ',30(I2,'       '))
      WRITE(16,507) (INT(AA(4,j)), j=3,icount+2)
 507  FORMAT('Xmin(iii)                ',30(I2,'       '))

      IF(CT .EQ. 1) THEN
```

```
      WRITE(16,511) (AA(2,j), j=3,icount+2)
 511  FORMAT('beta(iv)        ',30(1p,E16.8,'       '))
      WRITE(16,515) (INT(AA(7,j)), j=3,icount+2)
 515  FORMAT('X0(iv)          ',30(I2,'      '))
      WRITE(16,514) (INT(AA(6,j)), j=3,icount+2)
 514  FORMAT('Xmin(iv)        ',30(I2,'      '))
      END IF

      IF (CT .EQ. 1) THEN
      WRITE(16,512) (AA(3,j), j=3,icount+2)
      ELSE
      WRITE(16,518) (AA(3,j), j=3,icount+2)
      END IF
 512  FORMAT('gamma(iii,iv)          ',30(1p,E16.8,'       '))
 518  FORMAT('gamma(iii)             ',30(1p,E16.8,'       '))

      WRITE(16,516) (INT(AA(8,j)), j=3,icount+2)
 516  FORMAT('TLrS            ',30(I2,'      '))
      WRITE(16,517) (INT(AA(9,j)), j=3,icount+2)
 517  FORMAT('TLrE            ',30(I2,'      '))
      WRITE(16,508) (AA(10,j), j=3,icount+2)
 508  FORMAT('Residual        ',30(1p,E16.8,'       '))

      WRITE(16,551) (i, i=1,icount)
 551  FORMAT('DP      Type-2_TL_CLD_(displaced)     ',30('Fit',I1,'       '))

      DO 537 i=1,Xmax-10
        IF(i .GT. iTL) THEN
        WRITE(16,*) ''
        GO TO 537
        END IF
      WRITE(16,503) INT(AA(10+i,1)),(AA(10+i,j), j=2,icount+2)
 503  FORMAT(I4,'      ',30(1p,E16.8,'       '))
 537  CONTINUE

      RETURN
      END


!     1
      DOUBLE PRECISION FUNCTION FUN1(X)
      Implicit real*8 (a-h, o-z)
      DOUBLE PRECISION A(1000,1000),b(1000)

      DOUBLE PRECISION Bmask(1000),EXPX(1000),q
      DOUBLE PRECISION AA(1000,1000),AAA(1000)
      DOUBLE PRECISION b1(1000),b2(1000),b3(1000)

      INTEGER DP(1000),EXPmax,EXPTLmax
      DOUBLE PRECISION EXPNde(1000)

      DOUBLE PRECISION EXPN(1000),EXPN1(1000),EXPN1d(1000)

      DOUBLE PRECISION wSL(1000),wTL(1000)

      DOUBLE PRECISION beta1,beta2
      DOUBLE PRECISION betai,betaii,beteiii,betaiv
      DOUBLE PRECISION gammai_ii,gammaiii_iv
      DOUBLE PRECISION gamma

      INTEGER Xmin1,Xmin2,X01,X02
      INTEGER Xmini,Xminii,Xminiii,Xminiv,X0i,X0ii,X0iii,X0iv

      INTEGER XminiA(1000),X0iA(1000)

      INTEGER Xmax,Norm
      INTEGER icount,CT,op0,opi,opii,opiii,opiv

      INTEGER opri,oprii,opriii,opriv
      INTEGER mingap

      INTEGER SLrS,SLrE,TLrS,TLrE,TLstart

      INTEGER N,COUNT
      DOUBLE PRECISION X(10),DX(10),ACCX(10),DXp(10),ACCXp(10)

      INTEGER XmaxSEC,xpo
      DOUBLE PRECISION xa(1000),ya(1000),ypo,mypo
```

```
      DOUBLE PRECISION nmaxva

      DOUBLE PRECISION resSL, resTL

      CHARACTER label(16)
      character DPNAM(3),EXPNAM(40)

      common /tran1/ NP,N1,MP
      common /tran2/ XMAX,Norm
      common /tran6/ SLrS,SLrE,TLrS,TLrE,TLstart
      common /tran5/ EXPN,EXPN1,EXPN1d
      common /tran9/ wSL,wTL
      common /tran8/ Xmini,Xminii,Xminiii,Xminiv,X0i,X0ii,X0iii,X0iv
      common /tran4/ gamma
      common /tran7/ b
      common /tran10/ resSL,resTL
      common /tran11/ EXPNAM
      common /tran12/ iTL,CT,mingap

      FUN1=0.
      resSL=0.

!      WRITE(6,*) ''
!      WRITE(6,7) X(1),X(2),Xmini,X0i,Xminii,X0ii,SLrS,SLrE
!   7  FORMAT('beta(i) =      ',1p,E16.8,/,'beta(ii) =      ',1p,E16.8,/,
!     1 'Xmin(i) =    ',I2,/,'X0(i) =       ',I2,/,'Xmin(ii) =    ',I2,/,
!     1 'X0(ii) =     ',I2,/,'SLrS = ',I2,/,'SLrE = ',I2)

!      WRITE(6,*) ''
!      WRITE(6,*) 'X   EXPN'
!      DO 22 i=1,100
!      WRITE(6,10) i,EXPN(i)
!  22  CONTINUE

  10  FORMAT(I3,'      ',1p,E16.8)

      call minga(X(1),X(2),gamma,Xmini,Xminii,X0i,X0ii,b)

      CALL maxv(b,80,nmaxva)

      DO 2 i=1,XMAX
      b(i)=b(i)/nmaxva
   2  CONTINUE

      CALL maxv(b,80,nmaxva)

      DO 3 i=SLrS,SLrE
      FUN1=FUN1+wSL(i)*((1.-(b(i)/EXPN(i)))**2.)

      resSL=resSL+((1.-(b(i)/EXPN(i)))**2.)

      IF (X(1) .LT. 0.) FUN1=FUN1+EXP(ABS(X(1)))
      IF (X(2) .LT. 0.) FUN1=FUN1+EXP(ABS(X(2)))

!      IF (b(SLrE) .GT .EXPN(SLrE))
!     1 FUN1=FUN1+EXP(b(SLrE)-EXPN(SLrE))
   3  CONTINUE

!      WRITE(6,5) X(1),X(2),gamma
!   5  FORMAT(E16.8,'  ',1p,E16.8,'   '1p,E16.8)
!      WRITE(6,*) 'DP  EXP     CAL'
!      DO 110 ,j=1,EXPmax
!        WRITE(6,111) j,EXPN(j),b(j)
! 111    FORMAT(I3,'   ',1p,e16.8,'   ',1p,e16.8)
! 110  CONTINUE

      RETURN
      END

!     2
      DOUBLE PRECISION FUNCTION FUN2(X)
      Implicit real*8 (a-h, o-z)
      DOUBLE PRECISION A(1000,1000),b(1000)

      DOUBLE PRECISION Bmask(1000),EXPX(1000),q
      DOUBLE PRECISION AA(1000,1000),AAA(1000)
      DOUBLE PRECISION b1(1000),b2(1000),b3(1000)

      INTEGER DP(1000),EXPmax,EXPTLmax
```

```
      DOUBLE PRECISION EXPNde(1000)

      DOUBLE PRECISION EXPN(1000),EXPN1(1000),EXPN1d(1000)

      DOUBLE PRECISION wSL(1000),wTL(1000)

      DOUBLE PRECISION beta1,beta2
      DOUBLE PRECISION betai,betaii,beteiii,betaiv
      DOUBLE PRECISION gammai_ii,gammaiii_iv
      DOUBLE PRECISION gamma

      INTEGER Xmin1,Xmin2,X01,X02
      INTEGER Xmini,Xminii,Xminiii,Xminiv,X0i,X0ii,X0iii,X0iv

      INTEGER XminiA(1000),X0iA(1000)

      INTEGER Xmax,Norm
      INTEGER icount,CT,op0,opi,opii,opiii,opiv

      INTEGER opri,oprii,opriii,opriv
      INTEGER mingap

      INTEGER SLrS,SLrE,TLrS,TLrE,TLstart

      INTEGER N,COUNT
      DOUBLE PRECISION X(10),DX(10),ACCX(10),DXp(10),ACCXp(10)

      INTEGER XmaxSEC,xpo
      DOUBLE PRECISION xa(1000),ya(1000),ypo,mypo

      DOUBLE PRECISION nmaxva

      DOUBLE PRECISION resSL, resTL

      CHARACTER label(16)
      character DPNAM(3),EXPNAM(40)

      common /tran1/ NP,N1,MP
      common /tran2/ XMAX,Norm
      common /tran6/ SLrS,SLrE,TLrS,TLrE,TLstart
      common /tran5/ EXPN,EXPN1,EXPN1d
      common /tran9/ wSL,wTL
      common /tran8/ Xmini,Xminii,Xminiii,Xminiv,X0i,X0ii,X0iii,X0iv
      common /tran4/ gamma
      common /tran7/ b
      common /tran10/ resSL,resTL
      common /tran11/ EXPNAM
      common /tran12/ iTL,CT,mingap

      FUN2=0.
      resTL=0.

!      WRITE(6,*) ''
!      WRITE(6,7) X(1),X(2),Xminiii,X0iii,Xminiv,X0iv,TLrS,TLrE
!   7  FORMAT('beta(iii) =    ',1p,E16.8,/,'beta(iv) =      ',1p,E16.8,/,
!     1 'Xmin(iii) =   ',I2,/,'X0(iii) =      ',I2,/,'Xmin(iv) =     ',I2,/,
!     1 'X0(iv) =      ',I2,/,'TLrS = ',I2,/,'TLrE = ',I2)

!      WRITE(6,*) ''
!      WRITE(6,*) 'X   EXPN1d'
!      DO 22 i=1,100
!      WRITE(6,10) i,EXPN1d(i)
!  22  CONTINUE

  10  FORMAT(I3,'      ',1p,E16.8)

      call minga(X(1),X(2),gamma,Xminiii,Xminiv,X0iii,X0iv,b)

      CALL maxv(b,80,nmaxva)

      DO 2 i=1,XMAX
      b(i)=b(i)/nmaxva
   2  CONTINUE

!      WRITE(6,*) ''
!      WRITE(6,*) 'X   b'
!      DO 23 i=1,100
!      WRITE(6,10) i,b(i)
!  23  CONTINUE
```

```
      DO 3 i=TLrS,TLrE
      IF (i.LE.Xminiv+2 .AND .b(i) .GT. EXPN1d(i)) THEN
      FUN2=FUN2+wTL(i)*15*((1.-(b(i)/EXPN1d(i)))**2.)
      ELSE
      FUN2=FUN2+wTL(i)*((1.-(b(i)/EXPN1d(i)))**2.)
      END IF

      resTL=resTL+((1.-(b(i)/EXPN1d(i)))**2.)

      IF (X(1) .LT. 0.) FUN2=FUN2+EXP(ABS(X(1)))
      IF (X(2) .LT. 0.) FUN2=FUN2+EXP(ABS(X(2)))

!      IF (b(TLrE) .GT .EXPN1d(TLrE))
!     1 FUN2=FUN2+EXP(b(TLrE)-EXPN1d(TLrE))

   3  CONTINUE

!      WRITE(6,5) X(1),X(2),gamma
!   5  FORMAT(E16.8,'  ',1p,E16.8,'   '1p,E16.8)
!      WRITE(6,*) 'X   EXP     CAL'
!      DO 110 ,j=1,EXPmax
!        WRITE(6,111) j,EXPN1d(j),b(j)
! 111    FORMAT(I3,'   ',1p,e16.8,'   ',1p,e16.8)
! 110  CONTINUE

      RETURN
      END

!     2.1
      Subroutine maxv(b,nmax,nmaxva)
      Implicit real*8 (a-h, o-z)
      DOUBLE PRECISION b(1000),bb(1000),c,nmaxva
      INTEGER nmax

      DO 3 i=1,nmax
      bb(i)=b(i)
   3  CONTINUE

      DO 1 i=1,nmax-1
      DO 2 j=1,nmax-1
      IF(bb(i) .GT. bb(j+1)) GO TO 2
      c=bb(i)
      bb(i)=bb(j+1)
      bb(j+1)=c
   2  CONTINUE
   1  CONTINUE

      nmaxva=bb(1)

      RETURN
      END

!     3
      Subroutine minga(beta1,beta2,gamma,Xmin1,Xmin2,X01,X02,b)
      Implicit real*8 (a-h, o-z)
      DOUBLE PRECISION A(1000,1000),b(1000)

      DOUBLE PRECISION Bmask(1000),EXPX(1000),q
      DOUBLE PRECISION AA(1000,1000),AAA(1000)
      DOUBLE PRECISION b1(1000),b2(1000),b3(1000)

      INTEGER DP(1000),EXPmax,EXPTLmax
      DOUBLE PRECISION EXPNde(1000)

      DOUBLE PRECISION EXPN(1000),EXPN1(1000),EXPN1d(1000)

      DOUBLE PRECISION wSL(1000),wTL(1000)

      DOUBLE PRECISION beta1,beta2
      DOUBLE PRECISION betai,betaii,beteiii,betaiv
      DOUBLE PRECISION gammai_ii,gammaiii_iv
      DOUBLE PRECISION gamma

      INTEGER Xmin1,Xmin2,X01,X02
      INTEGER Xmini,Xminii,Xminiii,Xminiv,X0i,X0ii,X0iii,X0iv

      INTEGER XminiA(1000),X0iA(1000)
```

```
      INTEGER Xmax,Norm
      INTEGER icount,CT,op0,opi,opii,opiii,opiv

      INTEGER opri,oprii,opriii,opriv
      INTEGER mingap

      INTEGER SLrS,SLrE,TLrS,TLrE,TLstart

      INTEGER N,COUNT
      DOUBLE PRECISION X(10),DX(10),ACCX(10),DXp(10),ACCXp(10)

      INTEGER XmaxSEC,xpo
      DOUBLE PRECISION xa(1000),ya(1000),ypo,mypo

      DOUBLE PRECISION nmaxva

      DOUBLE PRECISION resSL, resTL

      INTEGER mx

      CHARACTER label(16)
      character DPNAM(3),EXPNAM(40)

      common /tran1/ NP,N1,MP
      common /tran2/ XMAX,Norm
      common /tran6/ SLrS,SLrE,TLrS,TLrE,TLstart
      common /tran5/ EXPN,EXPN1,EXPN1d
      common /tran9/ wSL,wTL
!      common /tran8/ Xmini,Xminii,Xminiii,Xminiv,X0i,X0ii,X0iii,X0iv
!      common /tran4/ gamma
!      common /tran7/ b
      common /tran10/ resSL,resTL
      common /tran11/ EXPNAM
      common /tran12/ iTL,CT,mingap

      IF (mingap .EQ. 1) THEN
      mx=3
      ELSE IF (mingap .EQ. 3) THEN
      mx=5
      ELSE
      mx=11
      END IF

      algga = -1.5

      DO 70 i=1,mx
        IF(i .EQ. 1)  dlgga  = .1
        IF(i .EQ. 2)  dlgga  = .03
        IF(i .EQ. 3)  dlgga  = .01
        IF(i .EQ. 4)  dlgga  = .003
        IF(i .EQ. 5)  dlgga  = .001
        IF(i .EQ. 6)  dlgga  = .0003
        IF(i .EQ. 7)  dlgga  = .0001
        IF(i .EQ. 8)  dlgga  = .00003
        IF(i .EQ. 9)  dlgga  = .00001
        IF(i .EQ. 10) dlgga  = .000003
        IF(i .EQ. 11) dlgga  = .000001

  80    DO 71 j=1,500
          gamma=10.**algga
!          WRITE(6,*) algga

          call NofX(beta1,beta2,gamma,Xmin1,Xmin2,X01,X02,b)
          IF (b(Norm) .LT. 0.) GO TO 72
          GO TO 73
  72      algga=algga+dlgga
          GO TO 80
  71    CONTINUE
  73      algga=algga-dlgga
  70  CONTINUE

      gamma=10.**algga

      RETURN
      END

!     4
      Subroutine NofX(beta1,beta2,gamma,Xmin1,Xmin2,X01,X02,b)
      Implicit real*8 (a-h, o-z)
```

```
      DOUBLE PRECISION A(1000,1000),b(1000)

      DOUBLE PRECISION Bmask(1000),EXPX(1000),q
      DOUBLE PRECISION AA(1000,1000),AAA(1000)
      DOUBLE PRECISION b1(1000),b2(1000),b3(1000)

      INTEGER DP(1000),EXPmax,EXPTLmax
      DOUBLE PRECISION EXPNde(1000)

      DOUBLE PRECISION EXPN(1000),EXPN1(1000),EXPN1d(1000)

      DOUBLE PRECISION wSL(1000),wTL(1000)

      DOUBLE PRECISION beta1,beta2
      DOUBLE PRECISION betai,betaii,beteiii,betaiv
      DOUBLE PRECISION gammai_ii,gammaiii_iv
      DOUBLE PRECISION gamma

      INTEGER Xmin1,Xmin2,X01,X02
      INTEGER Xmini,Xminii,Xminiii,Xminiv,X0i,X0ii,X0iii,X0iv

      INTEGER XminiA(1000),X0iA(1000)

      INTEGER Xmax,Norm
      INTEGER icount,CT,op0,opi,opii,opiii,opiv

      INTEGER opri,oprii,opriii,opriv
      INTEGER mingap

      INTEGER SLrS,SLrE,TLrS,TLrE,TLstart

      INTEGER N,COUNT
      DOUBLE PRECISION X(10),DX(10),ACCX(10),DXp(10),ACCXp(10)

      INTEGER XmaxSEC,xpo
      DOUBLE PRECISION xa(1000),ya(1000),ypo,mypo

      DOUBLE PRECISION nmaxva

      DOUBLE PRECISION resSL, resTL

      CHARACTER label(16)
      character DPNAM(3),EXPNAM(40)

      common /tran1/ NP,N1,MP
      common /tran2/ XMAX,Norm
      common /tran6/ SLrS,SLrE,TLrS,TLrE,TLstart
      common /tran5/ EXPN,EXPN1,EXPN1d
      common /tran9/ wSL,wTL
!      common /tran8/ Xmini,Xminii,Xminiii,Xminiv,X0i,X0ii,X0iii,X0iv
!      common /tran4/ gamma
!      common /tran7/ b
      common /tran10/ resSL,resTL
      common /tran11/ EXPNAM
      common /tran12/ iTL,CT,mingap

!      WRITE(6,*) 'In NofX'
!      WRITE(6,*) 'i   beta(i) Xmin(i) X0(i)'
!      WRITE(6,40) 1,beta1,Xmin1,X01
!  40  FORMAT(I1,'     ',F11.8,'      ',I2,'  ',I2)
!
!      WRITE(6,41) 2,beta2,Xmin2,X02
!  41  FORMAT(I1,'     ',F11.8,'      ',I2,'  ',I2)
!
!      WRITE(6,43) XMAX,Norm
!  43  FORMAT('XMAX  = 'I4,/,'Norm  = 'I4)

!     Set all elements in all arrays to zero
      DO 100 i=1,XMAX
        b(i) =0.
        DO 101 j=1,XMAX
          A(i,j)=0.
101     continue
100   continue

!     Setting up arrays
      DO 52 i=1,XMAX

!     SS
```

```
        IF (i.GT.1) A(i,i-1) = 1.
        A(i,i) = -1.

!     SBE1
        IF (i.GE.XMIN1+X01) A(i,i)=A(i,i)-beta1

        DO 53 j=i+Xmin1,XMAX
         IF (i.GE.X01) A(i,j)=A(i,j)+(beta1/(DBLE(j-Xmin1-X01+1)))
  53    CONTINUE

        DO 54 j=i+X01,XMAX
          IF (i.GE.Xmin1) A(i,j)=A(i,j)+(beta1/(DBLE(j-XMIN1-X01+1)))
  54    CONTINUE

!     SBE2
        IF (i.GE.XMIN2+X02) A(i,i)=A(i,i)-beta2

        DO 55 j=i+Xmin2,XMAX
         IF (i.GE.X02) A(i,j)=A(i,j)+(beta2/(DBLE(j-Xmin2-X02+1)))
  55    CONTINUE

        DO 56 j=i+X02,XMAX
          IF (i.GE.Xmin2) A(i,j)=A(i,j)+(beta2/(DBLE(j-XMIN2-X02+1)))
  56    CONTINUE

!     DBE12
        A(i,i) =A(i,i)-gamma
  52  CONTINUE

!     Replace row Norm with normalization
        DO 80 j=1,XMAX
          A(Norm,j)=1.
  80    CONTINUE
        b(Norm)=1.

      CALL GAUSSJ(A,XMAX,NP,B,N1,MP)
      RETURN
      END



!-----------------------------------------------------------------
      SUBROUTINE GAUSSJ(A,N,NP,B,M,MP)
!  Lin Eqn solver - gauss-Jordan elimination
!  Numerical Recipes (C) ch 2.
! on Input:  A= matrix (NP x NP); B = vector (M=1)
! on Output: A = inverse;         B = solution
      PARAMETER (NMAX=1000)
      Implicit real*8 (a-h, o-z)
!      Real*8 A(NP,NP),B(NP,MP),IPIV(NMAX),INDXR(NMAX),INDXC(NMAX)
      Real*8 A(1000,1000),B(1000,1)
      dimension IPIV(1000),INDXR(1000),INDXC(1000)
      DO 11 J=1,N
        IPIV(J)=0
11    CONTINUE
      DO 22 I=1,N
        BIG=0.
        DO 13 J=1,N
          IF(IPIV(J).NE.1)THEN
            DO 12 K=1,N
              IF (IPIV(K).EQ.0) THEN
                IF (ABS(A(J,K)).GE.BIG)THEN
                  BIG=ABS(A(J,K))
                  IROW=J
                  ICOL=K
                ENDIF
              ELSE IF (IPIV(K).GT.1) THEN
                PAUSE 'Singular matrix'
              ENDIF
12          CONTINUE
          ENDIF
13      CONTINUE
        IPIV(ICOL)=IPIV(ICOL)+1
        IF (IROW.NE.ICOL) THEN
          DO 14 L=1,N
            DUM=A(IROW,L)
            A(IROW,L)=A(ICOL,L)
            A(ICOL,L)=DUM
14        CONTINUE
```

```
      DO 15 L=1,M
        DUM=B(IROW,L)
        B(IROW,L)=B(ICOL,L)
        B(ICOL,L)=DUM
15      CONTINUE
      ENDIF
      INDXR(I)=IROW
      INDXC(I)=ICOL
      IF (A(ICOL,ICOL).EQ.0.) PAUSE 'Singular matrix.'
      PIVINV=1./A(ICOL,ICOL)
      A(ICOL,ICOL)=1.
      DO 16 L=1,N
        A(ICOL,L)=A(ICOL,L)*PIVINV
16    CONTINUE
      DO 17 L=1,M
        B(ICOL,L)=B(ICOL,L)*PIVINV
17    CONTINUE
      DO 21 LL=1,N
        IF(LL.NE.ICOL)THEN
          DUM=A(LL,ICOL)
          A(LL,ICOL)=0.
          DO 18 L=1,N
            A(LL,L)=A(LL,L)-A(ICOL,L)*DUM
18        CONTINUE
          DO 19 L=1,M
            B(LL,L)=B(LL,L)-B(ICOL,L)*DUM
19        CONTINUE
        ENDIF
21    CONTINUE
22  CONTINUE
    DO 24 L=N,1,-1
      IF(INDXR(L).NE.INDXC(L))THEN
        DO 23 K=1,N
          DUM=A(K,INDXR(L))
          A(K,INDXR(L))=A(K,INDXC(L))
          A(K,INDXC(L))=DUM
23      CONTINUE
      ENDIF
24  CONTINUE
    RETURN
    END

    SUBROUTINE FUNMIN(FUN,X,DX,N,ACCX,CONV,COUNT)
    IMPLICIT DOUBLE PRECISION (A-H,O-Z)
    INTEGER N,int1,int2
    DOUBLE PRECISION X(20),DX(20),ACCX(20)
     LOGICAL CONV
!**********************************************************************
!
!      SUBROUTINE FUNMIN
!
!      PURPOSE
!           MINIMISE A FUNCTION OF N VARIABLES
!
!      USAGE
!         CALL FUNMIN(FUN,X,DX,N,ACCX,CONV,COUNT)
!
!      DESCRIPTION OF PARAMETERS
!         FUN  - NAME OF FUNCTION FUN(X) TO BE MINIMIZED
!                (requires EXTERNAL statement in main program)
!         X    - INPUT VECTOR OF N INITIAL VALUES OF VARIABLES
!                OUTPUT VECTOR OF VARIABLES CORRESPONDING TO THE
!                MINIMUM VALUE OF FUN
!         DX   - INPUT VECTOR OF N INITIAL INCREMENTS OF VARIABLES.
!                OUTPUT VECTOR OF SUITABLY ALTERED STEP SIZES FOR RE-ENTRY.
!         N    - THE NUMBER OF VARIABLES  (TYPE INTEGER)
!         ACCX - INPUT VECTOR SPECIFYING MAX TOLERABLE RANGE OF EACH VARIABLE.
!                WHEN THE RANGES ALL FALL TO LESS THAN THESE LIMITS, THE
!                MINIMISATION IS ASSUMED TO HAVE CONVERGED.
!         CONV - LOGICAL OUTPUT VARIABLE WHOSE VALUE IS ONLY .TRUE. IF
!                RANGE OF EACH VARIABLE IS LESS THAN CORRESPONDING COMPONENT
!                OF "ACCX".
!         COUNT- INTEGER INPUT VARIABLE CONTAINING THE MAXIMUM NUMBER OF
!                FUNCTION EVALUATIONS ALLOWED.
!
!      REMARKS
!         CALLING PROGRAM MUST SPECIFY
!                EXTERNAL FUNCTION NAME
!
```

```
!      SUBROUTINES REQUIRED
!         REPLAS - SUPPLIED
!         NEW    - SUPPLIED
!
!      METHOD
!         DESCRIBED IN "A SIMPLEX METHOD FOR FUNCTION MINIMISATION"
!                BY J.A. NELDER AND R. MEAD, COMPUTER JOURNAL VOL 7
!                1965, P. 308. A FLOW CHART IS SUPPLIED FROM WHICH THIS
!                SUBROUTINE IS CONSTRUCTED.
!
!      MODIFIED
!         CONVERGENCE CRITERION ALTERED TO USE "ACCX" VECTOR,
!         BY D.HERBISON-EVANS    4 DEC 1972
!
!
!
!   LATEST UPDATE:  14 march 1989 (A R WHYTE) - elimination of
!   numerical arguments
!                                             in subroutine & function calls
!
      DOUBLE PRECISION XS(20),XSS(20)
      COMMON /BLOK1/ YS,YSS
      COMMON /BLOK2/ Y(20),VHI
      COMMON/BLOK4/ XX(21,20)
      COMMON /LOT/ J
       INTEGER V, COUNT, VLO, VNHI  ,VHI
      INTEGER FUNEV
      int1=1
      int2=2
!
!   IF MORE THAN 4 VARIABLES ARE TO BE MINIMISED THEN /BLOK4/
!   WILL HAVE TO BE ALTERED.
!
      IF (N.GT. 20) WRITE(6,122)
  122 FORMAT(//15H1"N" IS TOO BIG)
      IF (N.GT. 20) STOP
       J = N
!
!  FIND (N+1) INITIAL SIMPLEX VERTICES, GIVING (N+1) ESTIMATES OF FUN
!
      FUNEV=0
      DO 110 V = 1,N
       X(V) = X(V) + DX(V)
      DO 100 I = 1,N
  100  XX(V,I) = X(I)
      Y(V) = FUN(X)
      FUNEV=FUNEV+1
      X(V) = X(V) - DX(V)
  110  CONTINUE
      DO 115 I = 1,N
  115  XX(N+1,I) = X(I)
      Y(N+1) = FUN(X)
      FUNEV=FUNEV+1
       NONG = N + 1
!
!  FIND VERTICES GIVING HIGHEST(YHI), NEXT HIGHEST (YNHI) AND LOWEST
!  (YLO) ESTIMATES
!
  120  YLO = 1.0E20
       YHI = -YLO
      DO 140 V = 1,NONG
       YV = Y(V)
       IF (YV.GE. YLO) GOTO 130
       YLO=YV
       VLO=V
  130  IF (YV.LE.YHI) GOTO 140
       YHI=YV
       VHI=V
  140 CONTINUE
       YNHI=YLO
      DO 150 V=1,NONG
      YV=Y(V)
       IF (V .EQ. VHI .OR. YNHI .GE. YV) GOTO 150
      YNHI = YV
       VNHI = V
  150  CONTINUE
!
!  REFLECT HIGHEST ESTIMATE IN CENTROID OF REMAINING VERTICES TO
!  OBTAIN A NEW ESTIMATE YS AT VERTEX PS
```

```
!
      DO 160 I = 1,N
  160  XSS(I) = XX(VHI,I)
      arw1=2.0d0
      arw2=-1.0d0
      CALL NEW(XS,VHI,arw1,arw2,XSS)
      YS = FUN(XS)
      FUNEV=FUNEV+1
!
!  IF YS IS L5SS THAN YLO, EXPAND FURTHER FOR ANOTHER ESTIMATE YSS
!
       IF (YS .GE. YLO) GOTO 190
      arw1=-2.0d0
      arw2=3.0d0
      CALL NEW(XSS,VHI,arw1,arw2,XS)
      YSS = FUN(XSS)
      FUNEV=FUNEV+1
       IF (YSS - YLO) 240, 250, 250
!
!  EXPANDED ESTIMATE IS UNSATISFACTORY. CONTRACT FOR A NEW ESTIMATE
!
  190  IF (YS .LE. YNHI) GOTO 250
       IF (YS .GE. YHI) GOTO 200
      CALL REPLAS(int1,XS)
       YHI = YS
  200  DO 210 I = 1,N
  210  XS(I) = XX(VHI,I)
      arw1=0.5d0
      arw2=0.5d0
      CALL NEW(XSS,VHI,arw1,arw2,XS)
      YSS = FUN(XSS)
      FUNEV=FUNEV+1
!
!  IF YSS IS STILL NOT LESS THAN YLO, REPLACE ALL VALUES BY AN
!  "AVERAGE"
!
       IF (YSS .LE.YHI) GOTO 240
      DO 230 V = 1,NONG
      DO 220 I = 1,N
       DUM = 0.5*(XX(V,I) + XX(VLO,I))
       XX(V,I) = DUM
       XS(I) = DUM
  220  CONTINUE
       IF (V .EQ. VLO) GOTO 230
      Y(V) = FUN(XS)
      FUNEV=FUNEV+1
  230  CONTINUE
       GOTO 260
  240 CALL REPLAS (int2,XSS)
       GOTO 260
  250 CALL REPLAS(int1,XS)
!
!  INCREASE REPLACEMENT COUNT AND CHECK STANDARD DEVIATION FOR
!  CONVERGENCE
!
  260 YS=0
       YSS = 0.0
      DO 270 V = 1, NONG
       YV = Y(V)
       YS = YS + YV
       YSS = YSS + YV*YV
  270  CONTINUE
       ANON = NONG
!
!  FIND STANDARD DEVIATION OF VARIABLES SPECIFYING THE BEST (N+1)
!  ESTIMATES OF FUN AND STORE IN DX
!
      DO 290 I = 1,N
       YS = 0.0
       YSS = 0.0
      DO 280 V = 1,NONG
       XXVI = XX(V,I)
       YS = YS + XXVI
       YSS = YSS + XXVI*XXVI
  280  CONTINUE
       DX(I) = DSQRT(ABS(YSS/ANON -(YS/ANON)**2))
  290  CONTINUE
      CONV=.TRUE.
      DO 129 I=1,N
```

```
      CONV= CONV .AND. (DX(I).LT.ACCX(I))
  129 CONTINUE
!
!   IF COUNT NOT EXCEEDED AND NOT CONVERGERD - ITERATE.
      IF (.NOT.CONV.AND.(FUNEV.LT.COUNT)) GOTO 120
      IF(.NOT. CONV) WRITE(6,10)
   10 FORMAT(14H0NOT CONVERGED)
!       IF (CONV) WRITE(6,11)
   11 FORMAT(10H0CONVERGED)
!
!  PLACE VARIABLES FOR MINIMUN FUN IN X
!
      YLO=1.0E20
      DO 310 V = 1,NONG
       YV = Y(V)
       IF (YLO .LE.YV) GOTO 310
       YLO = YV
      DO 300 I = 1,N
  300  X(I) = XX(V,I)
  310  CONTINUE
!
!  EXIT WITH ARRAY X CONTAINING THE PARAMETERS FOR MINIMUM FUN, AND
!  ARRAY DX CONTAINING SUITABLE STEP SIZES FOR RE-ENTRY TO FUNMIN IF REQD.
!
      RETURN
      END
      SUBROUTINE NEW(ANEWX,NDUDV,CBAR,CI,XI)
      IMPLICIT DOUBLE PRECISION (A-H,O-Z)
      COMMON/BLOK4/XX(21,20)
      COMMON /LOT/ N
      DIMENSION ANEWX(20),XI(20)
       INTEGER V
       NUT = N  + 1
       AN = N
      DO 30 I = 1,N
       XBAR = 0.0
      DO 20 V = 1,NUT
   20  IF (V .NE. NDUDV) XBAR = XBAR + XX(V,I)
       XBAR = XBAR/AN
       ANEWX(I) = XBAR*CBAR + XI(I)*CI
   30  CONTINUE
      RETURN
      END
      SUBROUTINE REPLAS(NSTAR,ANEWX)
      IMPLICIT DOUBLE PRECISION (A-H,O-Z)
      COMMON /BLOK1/ YS,YSS
      COMMON/BLOK2/ Y(20),VHI
      COMMON/BLOK4/ XX(21,20)
      COMMON /LOT/ N
      DIMENSION ANEWX(20)
       INTEGER VHI
      DO 10 I = 1,N
   10  XX(VHI,I) =ANEWX(I)
       IF (NSTAR .EQ. 1) GOTO 20
       Y(VHI) = YSS
       GO TO 30
   20  Y(VHI) = YS
```
